# Supplementary material for: What is the financial burden to patients of accessing surgical care in Sierra Leone? A cross-sectional survey of catastrophic and impoverishing expenditure
Source: BMJ Open. 2021 Mar 8;11(3):e039049. doi: 10.1136/bmjopen-2020-039049 (PMC7942261; doi:10.1136/bmjopen-2020-039049)
Supplement: Supplementary data [file bmjopen-2020-039049supp001.pdf]

Appendix

Additional information on recruitment and training of research assistants

Research Assistants (RAs) were recruited through a competitive process and trained to administer the questionnaire. Training for all RAs was standardised and formally ran over 2 days. This involved; a formal presentation introducing the study, a review of all study processes and associated documents, a role play interview between the RAs using the questionnaire, a walk through the hospital to ensure the RAs gained an insight in to the surgical patients' journey and points at which OOP payments may be made or cost incurred and a review of clinical notes, ward admission books and theatre log books to ensure that all demographic and diagnostic information was accurately captured.

Appendix 1: Study questionnaire

Section 1: Demographics and admission questions

These questions are to be answered in conjunction with the patients notes and screening/recruitment sheet

Code	Question	Response
A1	Participant **unique ID**:	
A2	**Discharge** details	Care episode complete and discharged by Dr ☐<br>Transfer to another healthcare facility ☐<br>Self-discharging ☐ If ticked state reason__________________
A3	Where was the patient **admitted from**?	Connaught
		Direct to trauma ward / A&E / OPD ☐<br>Referral ☐ specify _________________<br>SOP (specialist outpatients) ☐<br>Through direct contact ☐<br>Transfer from the medical ward ☐<br>Other ☐ specify _______________
A4	Where is the patient being **discharged from**?	Connaught
		Trauma ward / A&E / OPD ☐<br>Surgical ward ☐<br>Annexe ☐<br>Other ☐ specify ___________
A5	**Age** of patient	_________________ / Don't know ☐ / Adult (AD) ☐
A6	**Sex**	Male ☐ Female ☐
A7	Usual **residential location** (address including district and from that state if urban or rural)	Address: ____<br>Urban ☐ Rural ☐
A8	Does the patient meet any exemption criteria for **free treatment**?	Yes ☐ No ☐<br>If yes, state which criteria (tick as applicable)<br>Aged under 5 years ☐<br>Pregnant ☐<br>Lactating ☐<br>Ebola survivor ☐<br>Destitute ☐<br>Disabled ☐
A9	**Type of admission**	Emergency (e.g trauma ward) ☐ Elective ☐
A10	**Primary diagnosis**	____
A11	What **treatment** did the patient receive? *(Note: Procedures are often done in the Trauma ward (minors procedure room) e.g skin traction/POP/suturing)*	**Operation/ procedure** ☐ (specify and go to A14)<br>Specify ____<br>**Non-operative** surgical care ☐ (go to A13)
A12	If patient received **non-operative care** state reason	Clinically appropriate / not recommended by Dr ☐<br>Patient chose not to have an operation / procedure ☐<br>Financial (unable to pay) ☐<br>Other ☐ (please give a brief statement)

Phull M, *et al. BMJ Open* 2021; 11:e039049. doi: 10.1136/bmjopen-2020-039049

		____
A13	**Length of hospital stay** (admission and discharge date)	**Admission date: _ _/_ _/_ _ _ _Discharge date:_ _/_ _/_ _ _ _**

Section 2a: Household structure and typical household income and expenditure

All questions are to be answered by the patient, parent, guardian or household member or head. ***Encourage patient to invite household head / member / breadwinner or the person who deals with household expenditure and or has made the payments for the care received - to help answer the questions.***

"Firstly are a few questions to understand the structure of your household (the people that eat food from the same pot and take instructions from the same head (excluding lodgers / individuals that pay to live in your house) and the average household income"

Code	Question	Response
B1	**Who** is being interviewed? Tick all that are applicable<br><br>If questions are being answered by someone other than the patient (e.g. household member), what is their relationship to the patient?	Patient ☐<br>Parent / Guardian ☐<br>Household head ☐<br>Other ☐ (State relationship to patient)<br>______________________________
B2	What is the **size of your household**, including yourself how many people normally eat food from the same pot and take instructions from the same head (exclude lodgers / individuals that pay to live in your house)	____________________
B3	Does **anyone in your household (household head/members) generate income**?	Yes ☐ (go to B4) No ☐ (go to B7)
B4	**How many people** in your household **generate income** that is used to support the household?	____________________<br>Don't know ☐
B5	What is the **occupation** of the person **who contributes the most** to your household expenses?	____________________<br>Salaried ☐ Non-salaried ☐<br>Don't know ☐
B6	**How much income does your household generate** (in total) to support the household in a typical month?	__________Leones Don't know ☐
B7	What is the **highest level of education of the main breadwinner (i.e the individual identified in B5)?**	No formal education ☐<br>Primary ☐<br>Secondary ☐<br>College ☐<br>University ☐<br>Other ☐ specify ________________
B8	Does your household **have any of the following**?	Electricity / Light Yes ☐ No ☐<br>Mobile phone Yes ☐ No ☐<br>Radio Yes ☐ No ☐<br>Television (TV) Yes ☐ No ☐<br>Computer Yes ☐ No ☐<br>Refrigerator Yes ☐ No ☐<br>Generator Yes ☐ No ☐<br>Bicycle Yes ☐ No ☐<br>Honda/ Motorcycle Yes ☐ No ☐<br>Car or truck Yes ☐ No ☐
B9	What material is used for the **roof of your house**?	Natural roofing (thatch) ☐<br>Basic roofing (tarpoline, metal, zinc) ☐<br>Finished roof (concrete / tiled) ☐<br>Other ☐ specify ______________
B10	What material is used for the **floor of your house**?	Natural floor (mud/earth/wattle) ☐<br>Basic floor (wood/cement) ☐<br>Finished floor (concrete/tile/carpet) ☐<br>Other ☐ specify ______________
B11	What material is used for the **walls of your house**?	Natural walls (mud/earth/wattle) ☐<br>Basic walls (stone/mud bricks/zinc) ☐<br>Finished walls (concrete) ☐<br>Other ☐ specify ______________

Section 2b: Typical household expenditure

"I would now like to ask you some questions about your household expenses starting with how much your household spends on food and consumption in a typical week"

Code	Question	Response
C1	Does the month of **Ramadhan** effect your household spending/expenses?	Yes ☐ No ☐<br>**If yes, say: please answer all the questions on expenses based on a week / month outside of the Ramadhan period**
C2	How much does your household spend on **food (including chop money)**?	________________Leones Don't know ☐
C3	How much does your household spend **drinks** (non-alcoholic drinks such as water, tea, coffee, milk and soft drinks?	________________Leones Don't know ☐
C4	**Tobacco and alcoholic beverages** (including beer, wine, spirits, poyo) – had at home / outside	________________Leones Don't know ☐
C5	Food eaten outside the dwelling (for example, at vendors, **cookery**, kiosks or restaurants)	________________Leones Don't know ☐
C6	**Other food** items (e.g kola nut, food not included in chop money)	________________Leones Don't know ☐
C7	Communication fees, including megabites (internet), mobile phone (**credit /top up)** and others?	________________Leones Don't know ☐
C8	**Transportation** (to work place, market, school etc)? (For example, petrol, taxis, motorbike taxis)	________________Leones Don't know ☐

"I would now like to ask you about other expenses your household might have had in the last month or a typical month"

Code	Question	Response
C9	**Utilities, such as water, light, electricity (NPA), waste disposal,** etc.?	________________Leones Don't know ☐
C10	**Fuel (e.g cooking / generator** - gas, coal, kerosene, firewood, petrol, diesel, etc.)?	________________Leones Don't know ☐
C11	**Personal toiletries** and personal care (e.g.soap, toothpaste, toothbrush, toilet roll, cosmetics, beauty salon, getting hair done etc.)?	________________Leones Don't know ☐
C12	**Clothing** and bedding?	________________Leones Don't know ☐
C13	**Loan repayments** (e.g on your house) / other **debt** or **microcredit** for business or other purposes?	________________Leones Don't know ☐
C14	**Entertainment**, including; cinemas/video centres to watch football matches, games, stadium for shows, soccer matches, or hangouts / chilling?	________________Leones Don't know ☐
C15	Payments for **household help**/servants, including cook, maid, driver, security, gardener, etc.?	________________Leones Don't know ☐
C16	How much does your household pay in **remittance** to family living away from home (**money, airtel/Africell money**, food etc)?	________________Leones Don't know ☐
C17	Excluding this hospital stay, how much money does your household usually spend on health care related to **"white man medicine",** including medicines, fees for doctors' appointments and hospital visits?	________________Leones Don't know ☐
C18	Excluding this hospital stay, how much money does your household usually spend on health care related to **"country/black man medicine**"?	________________Leones Don't know ☐
C19	Do you have any **other monthly expenditures**?	Yes ☐ Specify ____________________________<br>No ☐ (go to C21)
C20	**How much do these other expenditures** amount to?	________________Leones Don't know ☐
"I know these questions may be difficult to answer but try to give me the best estimate of expenses. I would now like you to focus on household expenses **over the last 12 months**. These are expenses that may be more periodic or "big purchases"		
C21	**Rent** (per year)	________________Leones Don't know ☐
C22	**Education fees and school supplies** (tuition, course fees, books)?	________________Leones Don't know ☐
C23	**Electrical goods** (mobile phones, televisions, DVD, radio, refrigerators,)?	________________Leones Don't know ☐
C24	**Big purchases** such as;<br>**Furniture** (tables, chairs, beds, sleeping mats)<br>**Vehicles** (trucks, cars, motorcycles, keke, bicycles) and **upkeep/repairs**?<br>**Tools** (brooms, nails, hammer, paint, shovel, gardening equipment etc)	 ________________Leones Don't know ☐<br>________________Leones Don't know ☐<br>________________Leones Don't know ☐
C25	**Hosting rituals or ceremonies** (funerals, birthdays, wedding, naming ceremony, pray de close, Christmas, the Haj)?	________________Leones Don't know ☐
C26	**Gifts for ceremonies** (funerals, birthdays, wedding, naming ceremony, pray de close, Christmas) if invited?	________________Leones Don't know ☐
C27	**Donations or contributions** to religious organisations (e.g. church/mosque)	________________Leones Don't know ☐
C28	**Deposit / upfront payments for property or land** (excluding monthly loans)?	________________Leones Don't know ☐
C29	Cleaning services or repair service (**house maintenance**)?	________________Leones Don't know ☐
C30	**Livestock** (chicken, goat, sheep etc)?	________________Leones Don't know ☐

C31	**Taxes** (city rate for home owner, vehicle tax, city council tax, NASSIT (national social security insurance trust)?	________________Leones Don't know ☐
C32	**Health insurance** premiums (including, community health insurance schemes) or pre-paid health plans?	________________Leones Don't know ☐

Section 3: Out-of-pocket payments for care sought prior to admission (from the day they fell ill with this illness)
"I would now like to ask you about all the health costs to your household before coming to hospital from when you fell sick with this illness / problem"

Code	Question	Response
D1	**When did you fall sick with the illness** that meant you had to be admitted to hospital?	______days before admission<br>______weeks before admission<br>______months before admission<br>Don't know ☐ (go to section 3 (C1)
"I would now like you to think about that period of time; **from falling sick to getting to the point of decision to come to Connaught / PCMH.** The following questions will be related to that time. I know that it may be difficult to think that far back but please give the best estimate you can of the costs"		
D2	Did you seek care for this illness elsewhere **before coming to this hospital**?	Yes ☐ (go to D3) No ☐ (go to section 4 (E1))
D3	**Where did you go** to get care for your illness before coming to this hospital? Tick all that are applicable<br><br>For each type of care provider ticked from the list below ask about the **number of visits** and a **cost break down**. If the patient cannot give a breakdown of costs ask for an **estimated total**<br>***Guidance notes;***<br>***Consultation** - fees to the provider ("doctors' fees"), fees excluding investigations and tests*<br>***Medications***<br>***Medical supplies** - Consumables, gloves, bandages, dressings etc*<br>***Investigations** – Includes labs, scans, x-rays and other imaging*<br>***Transportation** - transport for getting to and from the facilities, subsequent investigations/ getting treatments / medicines*	
	**Private clinic** ☐ no. of visits ___<br>How much was spent on;<br>Consultation _________________Leones Don't know ☐<br>Medications _________________Leones Don't know ☐<br>Medical supplies _______________Leones Don't know ☐<br>Investigations ________________Leones Don't know ☐<br>Transportation _______________Leones Don't know ☐<br>Other ______________________Leones Don't know ☐<br>**OR** Estimated total ____________Leones Don't know ☐<br><br>**Charity or religious clinic** ☐ no. of visits ___<br>How much was spent on;<br>Consultation _________________Leones Don't know ☐<br>Medications _________________Leones Don't know ☐<br>Medical supplies _______________Leones Don't know ☐<br>Investigations ________________Leones Don't know ☐<br>Transportation _______________Leones Don't know ☐<br>Other ______________________Leones Don't know ☐<br>**OR** Estimated total ____________Leones Don't know ☐<br><br>**Pharmacy, drug peddler, pepper Dr.** ☐ no. of visits ___<br>How much was spent on;<br>Consultation _________________Leones Don't know ☐<br>Medications _________________Leones Don't know ☐<br>Medical supplies _______________Leones Don't know ☐<br>Investigations ________________Leones Don't know ☐<br>Transportation _______________Leones Don't know ☐<br>Other ______________________Leones Don't know ☐<br>**OR** Estimated total ____________Leones Don't know ☐<br><br>**Traditional Healer / Herbalist** ☐ no. of visits ___	**Other government hospital** ☐ no. of visits ___<br>How much was spent on;<br>Consultation _________________Leones Don't know ☐<br>Medications _________________Leones Don't know ☐<br>Medical supplies _______________Leones Don't know ☐<br>Investigations ________________Leones Don't know ☐<br>Transportation _______________Leones Don't know ☐<br>Other ______________________Leones Don't know ☐<br>**OR** Estimated total ____________Leones Don't know ☐<br><br>**Private hospital** ☐ no. of visits ___<br>How much was spent on;<br>Consultation _________________Leones Don't know ☐<br>Medications _________________Leones Don't know ☐<br>Medical supplies _______________Leones Don't know ☐<br>Investigations ________________Leones Don't know ☐<br>Transportation _______________Leones Don't know ☐<br>Other ______________________Leones Don't know ☐<br>**OR** Estimated total ____________Leones Don't know ☐<br><br>**Community health clinic (Cheifdom level)** ☐<br>no. of visits ___<br>How much was spent on;<br>Consultation _________________Leones Don't know ☐<br>Medications _________________Leones Don't know ☐<br>Medical supplies _______________Leones Don't know ☐<br>Investigations ________________Leones Don't know ☐<br>Transportation _______________Leones Don't know ☐<br>Other ______________________Leones Don't know ☐<br>**OR** Estimated total ____________Leones Don't know ☐

	How much was spent on;<br>Consultation ________________Leones Don't know ☐<br>Medications ________________Leones Don't know ☐<br>Medical supplies ______________Leones Don't know ☐<br>Investigations _______________Leones Don't know ☐<br>Transportation ______________Leones Don't know ☐<br>Other _____________________Leones Don't know ☐<br>**OR** Estimated total ___________Leones Don't know ☐<br><br>**Traditional Birth Attendant (TBA) /Community Health Worker came home** ☐ no. of visits ___<br>How much was spent on;<br>Consultation ________________Leones Don't know ☐<br>Medications ________________Leones Don't know ☐<br>Medical supplies ______________Leones Don't know ☐<br>Investigations _______________Leones Don't know ☐<br>Transportation ______________Leones Don't know ☐<br>Other _____________________Leones Don't know ☐<br>**OR** Estimated total ___________Leones Don't know ☐<br><br>**Home delivery** ☐ no. of visits _____<br>How much was spent on;<br>Consultation ________________Leones Don't know ☐<br>Medications ________________Leones Don't know ☐<br>Medical supplies ______________Leones Don't know ☐<br>Investigations _______________Leones Don't know ☐<br>Transportation ______________Leones Don't know ☐<br>Other _____________________Leones Don't know ☐<br>**OR** Estimated total ___________Leones Don't know ☐	**Community health post** ☐ no. of visits ___<br>How much was spent on;<br>Consultation ________________Leones Don't know ☐<br>Medications ________________Leones Don't know ☐<br>Medical supplies ______________Leones Don't know ☐<br>Investigations _______________Leones Don't know ☐<br>Transportation ______________Leones Don't know ☐<br>Other _____________________Leones Don't know ☐<br>**OR** Estimated total ___________Leones Don't know ☐<br><br>**Maternity Child Health Post (MCHP)** ☐ no. of visits ___<br>How much was spent on;<br>Consultation ________________Leones Don't know ☐<br>Medications ________________Leones Don't know ☐<br>Medical supplies ______________Leones Don't know ☐<br>Investigations _______________Leones Don't know ☐<br>Transportation ______________Leones Don't know ☐<br>Other _____________________Leones Don't know ☐<br>**OR** Estimated total ___________Leones Don't know ☐<br><br>**Other** ☐ specify ____________________no. of visits ___<br>How much was spent on;<br>Consultation ________________Leones Don't know ☐<br>Medications ________________Leones Don't know ☐<br>Medical supplies ______________Leones Don't know ☐<br>Investigations _______________Leones Don't know ☐<br>Transportation ______________Leones Don't know ☐<br>Other _____________________Leones Don't know ☐<br>**OR** Estimated total ___________Leones Don't know ☐

Section 4: Out-of-pocket payments related to hospital admission

"I would now like to ask you about all the costs to your household from travel to the hospital to admission, your whole hospital stay and treatment through to discharge"

Please reiterate that as with all questions any questions related to informal payments / payments directly to staff are completely anonymous and no names are recorded or required. The purpose is to understand how much patients have to pay for all types of costs associated with their hospital admission so the following questions should be answered without undue concern.

If the household did not incur any of the expenses listed please state 0 Leones (this applies throughout the questionnaire)

Code	Question	Response
E1	Once the referral or decision was made to come to Connaught/PCMH, **how did you and all the attendants come / travel**?	Ambulance ☐<br>Public transport - Taxi / KK /Okada ☐<br>Private transport ☐<br>Other ☐ specify ________________________
E2	How much did this **transport cost?**	________________Leones Don't know ☐
E3	Did you have to pay any money for **registration**? If yes, ask how much the household paid for registration fees?<br><br>Tick all that are applicable	________________Leones Don't know ☐<br>Who or where was that paid to;<br>- Bank in Connaught ☐ ______________Leones<br>- Directly to hospital staff ☐ ______________Leones<br>- Other ☐ specify ________________ ________________Leones
E4	Did you have to pay any money for **admission** (fees / booklet)? If yes, ask how much the household paid for admission?<br><br>Tick all that are applicable	________________Leones Don't know ☐<br>Who or where was that paid to;<br>- Bank in Connaught ☐ ______________Leones<br>- Directly to hospital staff ☐ ______________Leones

		- Other ☐ specify ___________________<br>_______________Leones
E5	Did you have to pay any money at or for **triage (e.g for blood sugar)**? If yes, ask how much the household paid?<br><br>Tick all that are applicable	_______________Leones Don't know ☐<br>Who or where was that paid to;<br>- Bank in Connaught ☐<br>_______________Leones<br>- Directly to hospital staff ☐<br>_______________Leones<br>- Other ☐ specify ___________________<br>- _______________Leones
E6	Did you have to pay any money for any **helpers** (e.g porters, wheelchair, hospital staff to help you get around etc). If yes, ask how much the household paid?	_______________Leones Don't know ☐
E7	Did you have to pay any **doctors' fees / consultation fees**? If yes, ask how much the household paid in doctors' fees / consultation fees?<br><br>Tick all that are applicable	_______________Leones Don't know ☐<br>Who or where was that paid to;<br>- Bank in Connaught ☐<br>_______________Leones<br>- Directly to healthcare workers ☐<br>_______________Leones<br>- Other ☐ specify ___________________<br>_______________Leones
E8	Did you have any **laboratory (lab) tests**? If yes, ask how much money the household spent on these tests?<br><br>Tick all that are applicable	_________________Leones Don't know ☐<br>Who or where was that paid to;<br>- Bank in Connaught ☐<br>_______________Leones<br>- External lab ☐<br>_______________Leones<br>- Internal lab ☐<br>_______________Leones<br>- Directly to healthcare workers ☐<br>_______________Leones<br>- Other ☐ specify ___________________<br>_______________Leones
E9	Did you have any **x-rays, scans or imaging**? If yes, ask how much money your household spent on scans, imaging and x-rays?<br><br>Tick all that are applicable	_________________Leones Don't know ☐<br>Who or where was that paid to;<br>- Bank in Connaught ☐<br>_______________Leones<br>- External facility ☐<br>_______________Leones<br>- Internal facility ☐<br>_______________Leones<br>- Directly to healthcare workers ☐<br>_______________Leones<br>- Other ☐ specify ___________________<br>_______________Leones
E10	Did the patient have an **operation or procedure** (e.g skin traction/POP/suturing) in hospital?	Yes ☐ No ☐ (go to E15)
E11	How much did you pay for this **operation or procedure**?<br><br>Tick all that are applicable	_________________Leones. Don't know ☐<br>Who or where was that paid to;<br>- Bank in Connaught ☐<br>_______________Leones<br>- Directly to healthcare workers ☐<br>_______________Leones<br>- Other ☐ specify ___________________<br>_______________Leones
E12	Did you have to pay for **medications or medical supplies** (e.g. cannulas, IV lines, gauze, bandages, dressings, gloves, catheters and other consumables) **for theatre / your operation/procedure**? If yes, how much money did your household spend on these medicines and medical supplies?	_________________Leones. Don't know ☐<br>Who or where was that paid to;<br>- Bank in Connaught ☐<br>_______________Leones<br>- External pharmacy ☐<br>_______________Leones

	Tick all that are applicable	- Internal pharmacy / cost recovery ☐ ________________Leones<br>- Directly to healthcare workers ☐ ________________Leones<br>- Other ☐ specify ______________________ ________________Leones
E13	Did you have any **extra charges once in the operating room /area**? Please specify what this was for.	Yes ☐ (specify) No ☐ (go to E15)<br>**Specify what this was for?**<br>______________________________________
E14	How much money did your household spend on these **extra-charges in the operating room?**<br><br>Tick all that are applicable	____________________Leones. Don't know ☐<br>Who or where was that paid to;<br>- Bank in Connaught ☐ ________________Leones<br>- External pharmacy ☐ ________________Leones<br>- Internal pharmacy / cost recovery ☐ ________________Leones<br>- Directly to healthcare workers ☐ ________________Leones<br>- Other ☐ specify ______________________ ________________Leones
E15	Did your household have to arrange and get **blood for transfusion**?	Yes ☐ No ☐ (go to E18)
E17	How much money did your household spend on **blood transfusion related costs** (including blood bank fees, supplies, paying a donor etc.)<br><br>Tick all that are applicable	____________________Leones Don't know ☐<br>Who or where was that paid to;<br>- Bank in Connaught ☐ ________________Leones<br>- External facility ☐ ________________Leones<br>- Internal facility ☐ ________________Leones<br>- Directly to healthcare workers ☐ ________________Leones<br>- Other ☐ specify ______________________ ________________Leones
E18	Did you have to pay for any **medications in A&E/OPD or on the ward** (excluding prescriptions specifically for theatre)? If yes, ask how much the household paid for this?<br><br>Tick all that are applicable	____________________Leones Don't know ☐<br>Who or where was that paid to;<br>- Bank in Connaught ☐ ________________Leones<br>- External pharmacy ☐ ________________Leones<br>- Internal pharmacy / cost recovery ☐ ________________Leones<br>- Directly to healthcare workers ☐ ________________Leones<br>- Other ☐ specify ______________________ ________________Leones
E19	Did you have to pay for any **medical supplies in A&E/OPD and the Ward** (not including medications, e.g. cannulas, IV lines, gauze, bandages, dressings, gloves, catheters and other consumables) (excluding the prescription for theatre))? If yes, ask how much was paid?<br><br>Tick all that are applicable	____________________Leones. Don't know ☐<br>Who or where was that paid to;<br>- Bank in Connaught ☐ ________________Leones<br>- External pharmacy / facility ☐ ________________Leones<br>- Internal pharmacy / facility / cost recovery ☐ ________________Leones<br>- Directly to healthcare workers ☐ ________________Leones<br>- Other ☐ specify ______________________ ________________Leones
E20	Did you have to pay any **bed fees** (excluding admission fees / book? If yes, ask how much money the household paid in bed fees?	________________Leones Don't know ☐<br>Who or where was that paid to;<br>- Bank in Connaught ☐

	Tick <u>all</u> that are applicable	________________Leones<br>- Directly to healthcare workers ☐<br>________________Leones<br>- Other ☐ specify ______________________<br>________________Leones
E21	Did your household pay any money for **nursing care** (**requested** by nurses for care such as dressing changes etc, not including medications or medical supplies)? If yes, ask how much was paid?	_________________Leones Don't know ☐<br>Who or where was that paid to;<br>- Bank in Connaught ☐<br>________________Leones<br>- Directly to healthcare workers ☐<br>________________Leones<br>- Other ☐ specify ______________________<br>________________Leones
E22	Did your household give any **tips (ahjo)** i.e. voluntary and not requested money / gifts to healthcare workers / staff? If yes ask how much this amounted to?	____________________Leones Don't know ☐
E23	How many **family members / attendants / persons stayed in hospital** during your hospital stay (excluding visitors)?	_______________________people
E24	**Where did they stay** and **how much** did your household pay towards this?	In hospital ☐<br>Outside accommodation ☐<br>Other ☐ specify ___________________________<br>Total cost ______________Leones Don't know ☐
E25	During your hospital stay **where did you get food from**?<br><br>Tick <u>all</u> that are applicable	Provided by the hospital ☐<br>Bought by the patient / household ☐<br>Brought for the patient by someone else ☐<br>Other ☐ specify __________________________________
E26	How much did you / your household **spend on food** during your hospital stay? If they can't give a break down then ask for a total.	On patient _________________Leones Don't know ☐<br>For attendants ______________Leones Don't know ☐<br>Total _________________Leones Don't know ☐
E27	Did your household spend any money on transport for all the **"running" (transport to get food, hospital supplies, investigations and medications externally, excluding transport for visitors)**? If yes ask how much and state what this was for?<br>Tick <u>all</u> that are applicable	____________________Leones Don't know ☐<br>**What was this for?**<br>Food for the patients / attendants ☐<br>Going for tests / investigations outside ☐<br>Getting medicines / medical supplies ☐<br>Other ☐ specify ____________________
E28	Did your household have to pay any money at the time of **discharge**? If yes, ask how much was paid?	____________________Leones. Don't know ☐<br>Who or where was that paid to;<br>- Bank in Connaught ☐<br>________________Leones<br>- Directly to healthcare workers ☐<br>________________Leones<br>- Other ☐ specify ____________________<br>________________Leones
E29	Did your household have **any other illness related / hospital costs (e.g at SOP / other departments visited)**? If yes, specify what these costs where for.	Yes ☐ (specify and go to E30) No ☐ (go to F1)<br>**Specify**____
E30	How much did these **other costs** amount to?	____________________Leones. Don't know ☐<br>Who or where was that paid to;<br>- Bank in Connaught ☐<br>________________Leones<br>- Directly to healthcare workers ☐<br>________________Leones<br>- Other ☐ specify ____________________<br>________________Leones

Section 5: Indirect non-medical expenditure and how costs are met

"I will now ask you some questions about the broader implications of the costs and how you have met these costs?"

Code	Question	Response
F1	Do you have **health insurance/medical care** to cover any medical costs?	Yes ☐ No ☐ (go to F5)<br>Don't know ☐
F2	What **type of health insurance** do you have?	☐ Private insurance<br>☐ Work-related insurance

		☐ Other (specify)________________________
F3	What is your **monthly contribution towards your insurance** (if applicable)?	_________________Leones Don't know ☐
F4	Does **this insurance cover all (total) medical costs?**	Yes ☐ No ☐ If no, how much was paid in addition _____________Leones Don't know ☐
F5	**How did your household pay** for all the above costs? Tick all that are applicable	
	**Use savings**? Yes ☐ No ☐ Don't know ☐	How much? ________________Leones Don't know ☐
	**Borrow money**? Yes ☐ No ☐ Don't know ☐	How much?_______________Leones<br>Does this need to be paid back? Yes ☐ No ☐ Don't know ☐
	**Arrange family contributions?** Yes ☐ No ☐ Don't know ☐	How much?_______________Leones<br>Does this need to be paid back? Yes ☐ No ☐ Don't know ☐
	**Charity money from family/friends outside the country/church/social clubs?** Yes ☐ No ☐ Don't know ☐	How much?_______________Leones<br>Does this need to be paid back? Yes ☐ No ☐ Don't know ☐
	**Pledge / pawn any possessions** (including livestock, assets including electrical goods, generator, watches, jewellery etc.)?<br>Yes ☐ No ☐ Don't know ☐	How much money did this raise?_________________Leones<br>Don't know ☐
	**Sell any possessions or land?** Yes ☐ No ☐ Don't know ☐	How much money did this raise?_________________Leones<br>Don't know ☐
	**Any other way of meeting the hospital costs?**<br>Yes ☐ No ☐ Don't know ☐	Specify how ____________________________________<br>How much money did this raise?_________________Leones<br>Don't know ☐
F6	Did your household have to **stop sending any children to school, or pay reduced school fees** in order to pay for this hospitalisation?	Yes ☐ No ☐ Don't know ☐
F7	Did your household **loose any wages** due to this hospital stay?<br>If yes, ask how much was lost?	Yes ☐ No ☐ Don't know ☐<br>How much was lost ________________Leones<br>Don't know ☐
F8	Did you or anyone in your household **lose their job or change their role at work or home?**	Yes ☐ No ☐ Don't know ☐<br>If yes please specify<br>Lost job ☐<br>Took up employment / 2nd job ☐<br>Change of duties in household to meet costs ☐<br>Other ☐ specify ___________________________________

Any other comments?
____
____
____
____

Appendix table 2: Household expenditure showing imputed and non-imputed data sets.
Comparison of non-imputed and imputed data on household expenditure using Multiple Imputation Chained Equations to compute missing data-points using predictive mean matching.

Household expense	Non-imputed data mean (SD) (Le and $US)	Imputed data pooled mean (Le and $ US)
**Individual Consumption Expenditure by Households Including all variables collected**	**Le 39,665,597 (53,679,740) $ 4,425 (5,989)**	**Le 47,944,384 $5,349**
**Individual Consumption Expenditure by Households Excluding variables with > 20% missing data i.e. clothing, mobile phone credit and transport**	**Le 28,134,505 (31,539,987) $ 3,139 (3,519)**	**Le 31,988,507 $ 3,569**
Food and non-alcoholic beverages	Le 18,616,404	Le 20,867,118

Phull M, *et al. BMJ Open* 2021; 11:e039049. doi: 10.1136/bmjopen-2020-039049

	(22,364,391)<br>$ 2,077 (2,495)	$ 2,328
Alcoholic Beverages, Tobacco and Narcotics	Le 252,991<br>(1,047,612)<br>$ 28 (117)	Le 314,095<br>$ 35
Rental	Le 884,123<br>(4,670,009)<br>$ 99 (521)	Le 876,940<br>$ 98
Household maintenance	Le 108,092<br>(298,466)<br>$ 12 (33)	Le 130,857<br><br>$ 15
Electricity, gas and other fuels	Le 720,748<br>(1,068,694)<br>$ 80 (119)	Le 747,701<br>$ 83
Furnishings, household equipment and routine household maintenance	Le 632,577<br>(1,065,761)<br>$ 71 (119)	Le 699,188<br>$ 78
Healthcare (traditional and western medicine)	Le 561,770<br>(1,490,207)<br>$ 63 (166)	Le 740,205<br>$ 83
Recreation and cultural services	Le 821,684<br>(1,894,677)<br>$ 92 (211)	Le 1,121,965<br>$ 125
Education	Le 1,338,183<br>(2,295,578)<br>$ 149 (256)	Le 1,614,982<br>$ 180
Personal care / toiletries	Le 609,012<br>(629,656)<br>$ 68 (70)	Le 627,269<br>$ 70
Health insurance	Le 8,333<br>(138,661)<br>$ 1 (15)	Le 13,370<br>$ 1
Remittance	Le 974,408<br>(1,655,020)<br>$ 109 (185)	Le 1,015,363<br>$ 113
Donations	Le 185,233<br>(475,436)<br>$ 21 (53)	Le 203,246<br>$ 23
Livestock	Le 41,400<br>(213,804)<br>$ 5 (24)	Le 45,254<br>$5
Taxes	Le 59,500<br>(261,497)<br>$ 7 (29)	Le 73,723<br>$ 8

Appendix table 3: Household expenditure data. Variables on household expenditure shown here, for broad comparison, with the Economic and Financial survey Sierra Leone 2014 data[21]. Categories were harmonised where possible, however given differences in questions asked between surveys, an exact match of categories was not possible to achieve. Costs from the 2014 Economic and Financial Survey were not adjusted for inflation which needs to be considered when reviewing this data.

**Household consumption expenditure (in Leones (Le) and USD ($))**		
**Household Expense**	**Sierra Leone Economic and Financial Survey data 2014**	**Study data**
**Individual Consumption Expenditure by Households (Total Expenditure)**	**Le 15,414,816<br>$ 1,739**	**Le 31,988,507<br>$ 3,569**

**Food and non-alcoholic beverages**	**Le 6,838,365**<br>**$ 771**	**Le 20,867,118**<br>**$ 2,328**
Food	Le 6,644,019<br>$ 74	Le 17,925,090<br>$ 2,000
Non-alcoholic beverages	Le 194,346<br>$ 22	Le 2,942,028<br>$ 328
**Alcoholic Beverages, Tobacco and Narcotics**	**Le 450,612**<br>**$ 51**	**Le 314,095**<br>**$ 35**
**Housing, water, electricity, gas and other fuels**	**Le 1,058,449**<br>**$119**	**Le 875,672**<br>**$ 98**
**Rental**	**Le 253,948**<br>**$ 29**	**Le 876,940**<br>**$ 98**
**Maintenance and repair of the dwelling**	**Le 32,650**<br>**$ 4**	**Le 876,940**<br>**$ 98**
**Electricity, gas and other fuels**	**Le 595,832**<br>**$ 67**	**Le 747,701**<br>**$ 83**
**Furnishings, household equipment and routine household maintenance**	**Le 413,364**<br>**$ 47**	**Le 699,188**<br>**$ 78**
Furnishings	Le 88,058<br>$ 10	Le 196,217<br>$ 22
Household appliances	Le 41,821<br>$ 5	Le 314,772<br>$ 35
Tools and equipment for house and garden	Le 45,403<br>$ 5	Le 57,344<br>$ 6
Goods and services for routine household maintenance	Le 105,438<br>$ 12	Le 130,857<br>$ 15
Purchase of vehicles	Le 108,710<br>$ 12	Le 160,759<br>$ 18
Educations	Le 794,478<br>$ 90	Le 1,614,982<br>$ 180
Insurance (HI)	Le 38,890<br>$ 4	Le 13,370<br>$ 1

Appendix table 4: Imputed and non-imputed data for out-of-pocket costs for comparison.

Costs	Imputed<br>Mean cost<br>($US (% of subtotal))	Non-imputed<br>Mean cost ($US) (SD)
**Prehospital costs**		
**Direct pre-hospital medical OOP costs (total)**	**21 (88% of 24)**	**14 (65)**
- Consultation	2 (10% of 21)	1 (6)
- Medications	12 (57% of 21)	9 (46)
- Medical supplies	2 (10% of 21)	2 (8)
- Investigations	4 (19% of 21)	4 (25)
- Other miscellaneous	2 (10% of 21)	1 (10)
**Direct (pre-hospital) non-medical OOP costs (total)**	**3 (13% of 24)**	**3 (9)**
- Transport	3 (100% of 3)	3 (9)
Total pre-hospital costs	***24 (10% of 243)***	***25 (75)***
**In hospital costs**		
**Direct medical OOP costs (total)**	**138 (63% of 219)**	**109 (121)**
- Administrative	20 (14% of 138)	16 (24)
- Medications	26 (19% of 138)	25 (61)
- Medical supplies	14 (10% of 138)	11 (33)
- Investigations	15 (11% of 138)	14 (23)
- Blood transfusion	9 (7% of 138)	9 (22)
- Total operation costs	49 (36% of 138)	51 (75)
- Unofficial costs	6 (4% of 138)	5 (9)
- Other / miscellaneous	1 (1% of 138)	1 (8)
**Direct non-medical costs (total)**	**34 (16% of 219)**	**34 (34)**
- Transport to hospital	7 (21% of 34)	7 (17)
- Food	20 (59% of 34)	21 (20)
- Accommodation	0 (0% of 34)	0 (0)
- Other*	7 (21% of 34)	6 (10)
**Indirect costs**		
- Lost wages	46 (100% of 46)	35 (116)
**TOTAL OOP COSTS**	**243**	**176 (165)**

*other relates to travel and other associated costs incurred as a result for needed investigations from and or medication / supplies from an external facility. SPSS calculates only the mean using imputed variables, hence no standard deviation is displayed.

Appendix table 5: Route of payment for OOP costs; percentage of the total OOP costs by cost categories paid to bank / cashier, directly to staff or externally for different services accessed once tertiary level care was reached

Costs	% paid to Hospital bank / cashier	% paid directly to or via staff	% paid externally	% unknown	Total
**TOTAL in-hospital costs**	**32.64%**	**48.16%**	**16.50%**	**2.70%**	**100%**
**Administration**	**52.03%**	**42.53%**	-	5.44%	100%
- Registration fees	90.98%	8.71%	-	0.30%	100%
- Admission fees	66.78%	32.39%	-	0.83%	100%
- Triage fees	8.05%	91.95%	-	0.00%	100%
- Bed fees	19.47%	46.13%	-	34.40%	100%
- Discharge fees	41.19%	57.28%	-	1.53%	100%
**Investigations**	**19.39%**	**44.29%**	**33.73%**	**2.59%**	**100%**
- Laboratory	25.68%	40.29%	30.13%	3.90%	100%
- Imaging	14.43%	47.44%	36.57%	1.56%	100%
**Total operation costs**	**55.40%**	**32.89%**	**9.35%**	**2.35%**	**100%**
- Operation	80.10%	18.91%	-	1.00%	100%
- Medical supplies for the operation	13.42%	56.59%	25.31%	4.68%	100%
- Other / miscellaneous	69.77%	30.23%	-	0.00%	100%
**Blood transfusion**	**16.24%**	**67.68%**	**13.85%**	**2.23%**	**100%**
**Total medications and medical supplies for ward care**	**4.82%**	**61.00%**	**31.73%**	**2.45%**	**100%**
- Medications	2.19%	62.86%	31.91%	3.03%	100%
- Medical supplies	10.81%	56.74%	31.31%	1.14%	100%
**Informal payment**	**2.44%**	**97.56%**	**-**	**0.00%**	**100%**
- Doctors' fees	8.72%	91.28%	-	0.00%	100%
- Nursing care	0%	100%	-	0.00%	100%
- Porters	0%	100%	-	0.00%	100%
- Tips	0%	100%	-	0.00%	100%
**Other / miscellaneous costs**	**0%**	**98.37%**	**0**	**1.63%**	**100%**

Phull M, *et al. BMJ Open* 2021; 11:e039049. doi: 10.1136/bmjopen-2020-039049

Appendix table 6: Linear regression analysis showing odds of increasing in-hospital costs of care for each variable using a generalized linear model with imputed variables using a Tweedie 1.9 function.

Variable		Odds Ratio	95% CI	p-value
Sex	Female	ref		
	Male	1.05	(0.86-1.29)	0.63
Age	Age	1.02	(1.01-1.02)	0.00
Length of stay	Length of stay	1.02	(1.02 -1.03)	0.00
Type of admission	Elective admission	ref		
	Emergency admission	0.96	(0.75-1.24)	0.77
Category of operation	Non-operative	ref		
	Burns	1.33	(0.25-7.00)	0.74
	ENT	0.64	(0.36-1.166)	0.14
	General surgery	1.67	(1.29-2.17)	0.00
	General paediatric surgery	0.84	(0.57-1.24)	0.38
	Trauma and orthopaedic	1.30	(0.98- 1.74)	0.07
	Urology	2.08	(1.22-3.53)	0.01
Area of residence	Rural	ref		
	Urban	0.98	(0.76-1.25)	0.85

Phull M, *et al. BMJ Open* 2021; 11:e039049. doi: 10.1136/bmjopen-2020-039049
