# Supplementary material for: What is the financial burden to patients of accessing surgical care in Sierra Leone? A cross-sectional survey of catastrophic and impoverishing expenditure
Source: BMJ Open. 2021 Mar 8;11(3):e039049. doi: 10.1136/bmjopen-2020-039049 (PMC7942261; doi:10.1136/bmjopen-2020-039049)
Supplement: Author's manuscript [file bmjopen-2020-039049.draft_revisions.pdf]

BMJ Open is committed to open peer review. As part of this commitment we make the peer review history of every article we publish publicly available.

When an article is published we post the peer reviewers' comments and the authors' responses online. We also post the versions of the paper that were used during peer review. These are the versions that the peer review comments apply to.

The versions of the paper that follow are the versions that were submitted during the peer review process. They are not the versions of record or the final published versions. They should not be cited or distributed as the published version of this manuscript.

BMJ Open is an open access journal and the full, final, typeset and author-corrected version of record of the manuscript is available on our site with no access controls, subscription charges or pay-per-view fees (http://bmjopen.bmj.com).

If you have any questions on BMJ Open's open peer review process please email info.bmjopen@bmj.com

BMJ Open

Catastrophic and impoverishing expenditure for surgical care in Sierra Leone

Journal:	*BMJ Open*
Manuscript ID	bmjopen-2020-039049
Article Type:	Original research
Date Submitted by the Author:	02-Apr-2020

Complete List of Authors:	Phull, Manraj ; West Hertfordshire Hospitals NHS Trust<br>Grimes, Caris E.; King's College London Faculty of Life Sciences and Medicine; Medway NHS Foundation Trust<br>Kamara, Thaim; University of Sierra Leone College of Medicine and Allied Health Sciences, Surgery<br>Wurie, Haja ; University of Sierra Leone College of Medicine and Allied Health Sciences<br>Leather, Andy; King's College London Faculty of Life Sciences and Medicine, King's Centre for Global Health<br>Davies, Justine ; King's College London Faculty of Life Sciences and Medicine, Centre for Global Health; University of Birmingham Institute of Applied Health Research

Keywords:	SURGERY, HEALTH ECONOMICS, Health policy < HEALTH SERVICES ADMINISTRATION & MANAGEMENT

SCHOLARONE™ Manuscripts

I, the Submitting Author has the right to grant and does grant on behalf of all authors of the Work (as defined in the below author licence), an exclusive licence and/or a non-exclusive licence for contributions from authors who are: i) UK Crown employees; ii) where BMJ has agreed a CC-BY licence shall apply, and/or iii) in accordance with the terms applicable for US Federal Government officers or employees acting as part of their official duties; on a worldwide, perpetual, irrevocable, royalty-free basis to BMJ Publishing Group Ltd ("BMJ") its licensees and where the relevant Journal is co-owned by BMJ to the co-owners of the Journal, to publish the Work in this journal and any other BMJ products and to exploit all rights, as set out in our licence.

The Submitting Author accepts and understands that any supply made under these terms is made by BMJ to the Submitting Author unless you are acting as an employee on behalf of your employer or a postgraduate student of an affiliated institution which is paying any applicable article publishing charge ("APC") for Open Access articles. Where the Submitting Author wishes to make the Work available on an Open Access basis (and intends to pay the relevant APC), the terms of reuse of such Open Access shall be governed by a Creative Commons licence – details of these licences and which Creative Commons licence will apply to this Work are set out in our licence referred to above.

Other than as permitted in any relevant BMJ Author's Self Archiving Policies, I confirm this Work has not been accepted for publication elsewhere, is not being considered for publication elsewhere and does not duplicate material already published. I confirm all authors consent to publication of this Work and authorise the granting of this licence.

For peer review only

Catastrophic and impoverishing expenditure for surgical care in Sierra Leone

Manraj Phull (1), Caris E Grimes (2, 3), Thaim B Kamara (4), Haja Wurie (5), Andrew JM Leather (2) * and Justine Davies (2, 6, 7) *

*Co-senior authors

Authors

Manraj Phull BSc MBBS MRCS MSc
1) West Hertfordshire Hospitals NHS Trust. Watford General Hospital, Watford, Hertfordshire, UK

Caris E Grimes BSc MBBS MEd FRCS MD
2) King's Centre for Global Health and Health Partnerships, School of Population Health and Environmental Sciences, King's College London, London, SE5 9RJ, UK
3) Medway NHS Foundation Trust, Gillingham, Kent, UK

Thaim B Kamara MBChB, FWACS
4) College of Medicine and Allied Health Sciences, Freetown, Sierra Leone

Haja Wurie
5) College of Medicine and Allied Health Sciences, Freetown, Sierra Leone

Andrew JM Leather MBBS, FRCS, MS
2) King's Centre for Global Health and Health Partnerships, School of Population Health and Environmental Sciences, King's College London, London, SE5 9RJ, UK

Justine Davies (Corresponding Author) MBChB, MD (res), MRCP
2) King's Centre for Global Health and Health Partnerships, School of Population Health and Environmental Sciences, King's College London, London, SE5 9RJ, UK
6) Institute for Applied Health Research, University of Birmingham, Edgbaston, Birmingham B15 2TT UK
7) Medical Research Council/Wits University Rural Public Health and Health Transitions Research Unit, Faculty of Health Sciences, School of Public Health, University of the Witwatersrand, Johannesburg, South Africa

Correspondence to Justine Davies: J.Davies.6@bham.ac.uk

Author Contribution: JD, AL, TBK and HW conceptualised the study. MP, JD, and AL developed the protocol and survey tools; MP, JD, and CG analysed the data; all authors contributed to the interpretation of the results and write up of the manuscript; All authors approved the manuscript for publication.

Competing interests: None declared.

Funding: This research was partly funded by the National Institute of Health Research (NIHR) Global Health Research Unit on Health System Strengthening in Sub-Saharan Africa, King's College London (GHRU 16/136/54) using UK aid from the UK Government to support global health research. The views expressed in this publication are those of the author(s) and not necessarily those of the NIHR or the Department of Health and Social Care.

For peer review only - http://bmjopen.bmj.com/site/about/guidelines.xhtml

Data Sharing: Further data is available on reasonable request from the corresponding author.

Patient and Public Involvement / Cohort Description: The Lancet Commission on Global Surgery has shown that out of pocket expenditure limits patients ability to access surgical care when needed. Accessing care for a surgically treatable disease to reduce mortality or morbidity is a priority for patients. The methodology employed was standard for assessing out of pocket costs, wealth, and healthcare expenditure. Patients were not involved in designing these methods, however, they were involved in testing and refining them to ensure appropriateness to a local setting. No patients were involved in the recruitment to and conduct of the study.

As part of the ethics board approval, we did not collect contact details of the patients involved in this study and hence cannot disseminate the results to them. However, the results are being shared widely amongst policy workers, community leaders, and clinicians in Sierra Leone. The patient advocacy movement in Sierra Leone, like in many low income countries, is nascent, hence there are no patient groups with which to share results. We hope that our work will galvanise greater advocacy and enable sharing more widely.

We did not collect names of the six patients who helped to refine the data collection tool or ask their permission to be named. However, we have added a statement to the acknowledgements to generically acknowledge their contribution.

Acknowledgements: We thank the healthcare workers and patients who were involved in refining the data collection tool to ensure its applicability to a local setting.

For peer review only

Abstract

Background: Integral to providing Universal Health Coverage in lower- and middle-income countries (LMICs) is financial risk protection for the estimated 3.7 billion people facing financial catastrophe each year if they needed surgical care. To achieve this, a greater understanding of financial hardship on surgical patients is required. This study measured the financial burden associated with accessing surgical care in Sierra Leone.
Methods: A cross-sectional survey was conducted with patients at discharge from the main tertiary level hospital in Freetown. This captured demographics, yearly household expenditure, direct medical, direct non-medical, and indirect costs for surgical care, and summary household assets. Missing data were imputed. Rates of catastrophic expenditure (CE) (a cost > 10% of annual expenditure or >40% of capacity to pay), impoverishment (being pushed into, or further into, poverty as a result of surgical care costs), amount of OOP costs, and means used to meet these costs were derived.
Results: Of 335 patients interviewed, 39.4% were female and 80.3% were urban dwellers. Median yearly household expenditure was US$3569. Mean out-of-pocket costs were US$243, of which a mean of US$24 (10%) was spent pre-hospital. Of costs incurred during the hospital admission, direct medical costs were US$138 (63%) and US$34 (16%) were direct non-medical costs. US$46 (21%) were indirect costs. Catastrophic expenditure affected 10-18% of those interviewed, depending on definition. 45% of people were below the national poverty line prior to admission and 50% were pushed below or further into poverty following payment for surgical care. 83.7% of patients used household savings to meet OOP costs. Only 2% (6 patients) had health insurance.
Conclusion:
Obtaining surgical care has substantial economic impacts on households which pushes them into poverty or further into poverty. The much-needed scaling up of surgical care needs to be accompanied by financial risk protection.

Article Summary
Strengths and Limitations
Strengths:
Rigorous methodology to provide in depth data on costs of accessing surgical care.
Well recognised methods to ascertain household expenditure.
Provides accurate estimates of OOP, catastrophic, and impoverishing expenditure calculated as well as sources of financing.

Limitations
Data captured in one hospital only, although that is the major surgical care centre for the country.
Only examines those who accessed care and doesn't allow exploration of costs as a limitation to accessing care.

For peer review only

Introduction

An estimated 33 million individuals globally face financial catastrophe through payment for surgery and anaesthetic care each year. Furthermore 3·7 billion people have been estimated to be at risk of catastrophic expenditure (CE – defined as a total OOP health payment that exceeds a set threshold of the household's annual income or expenditure) due to a lack of financial risk protection (FRP).[1,2] Surgical conditions make up 30% of the global burden of disease and globally an additional 143 million surgical procedures are required annually to meet the current unmet surgical need.[1,3] To ensure universal health coverage, it is therefore essential that Financial Risk Protection (FRP) is prioritised alongside the scaling up of surgical care. The Lancet Commission on Global Surgery (LCoGS) stated a target of 100% financial protection by 2030 for people accessing surgical care, and FRP indicators for surgery are now included within the World Development Indicators (WDI).[4] Despite this, there is little information on financial implications of accessing surgery in the literature beyond modelled studies,[1,2,5] many of which have been based on few real-world data-points.

Worldwide modelled data on CE and impoverishing expenditure (IE – defined as being pushed into or further into poverty) related to surgical care reveals that those most affected are individuals in low- and middle-income countries (LMICs).[1,2,6] Modelling studies from Sierra Leone in West Africa, classed as "least developed" by the UN, and with a population of 7 million reflects these findings; between 84.7% and 49.9% of the population in Sierra Leone is estimated to be at risk of CE if they require surgery. Estimated average out-of-pocket (OOP) costs for major surgery in the country were US$117.60, which put 73.3% to 59.2% of the population at risk of impoverishment.[5,7] However, there are no empirical data to validate these estimates. The estimated unmet surgical burden of disease in SL is huge, at 92%, as a result of the historical neglect of surgical care both nationally and globally.[8-10] SL has recently shown its commitment to improving access to surgical care by embarking on developing a national surgical, obstetric and anaesthesia plan (NSOAP) aimed at scaling up and strengthening surgical services.[11] To enable effective planning, an accurate understanding of the financial implications of accessing surgical services is required.

In Sierra Leone, as in many LMICs, payments for healthcare are upfront, complex, and not immediately apparent from hospital listed service charges. In addition, hospital listed charges – where they exist – may not reflect the total facility-incurred costs that patients pay during their hospitalisation. These include direct medical costs which are charges for the payment of medical care and direct non-medical costs which include items such as transport to the hospital and food. In addition, substantial costs of care may be incurred prior to the hospitalisation episode. For example, there may be direct medical costs at other healthcare facilities visited prior to the definitive admission. Finally, there are indirect costs (e.g. loss of wages whilst receiving care) that patients, and in some cases their caregivers, experience in their illness, which also impact upon ability to access care. Two ways of capturing these costs is the measurement of IE or CE. The two most widely used thresholds for CE are an expense of > 40% of non-subsistence expenditure (i.e. household expenditure net of subsistence costs, as a means of capturing the ability to pay) or > 10% of total annual expenditure.[12-15]

This study aimed to measure the financial burden associated with receiving surgical care in Sierra Leone by using an exit survey to determine a) direct medical, direct non-medical, and indirect OOP costs to pay for a surgical care episode b) the rate of impoverishment and catastrophic expenditure, c) the wealth characteristics of the population accessing surgical care relative to that of the general Sierra Leonean population, and d) the factors associated with higher costs of hospital care. Finally, the in-hospital payment mechanism (i.e. where and to whom the OOP payments are being made), how costs of accessing surgical care are met, and the factors associated with meeting costs of care were explored.

For peer review only - http://bmjopen.bmj.com/site/about/guidelines.xhtml

Methods

Setting

This study was done in the main tertiary referral centre in Sierra Leone, located in the central part of greater Freetown, and where the majority of surgical care in the countries' non charitable sector is done. It is a 400-bed hospital with 150 beds dedicated to surgical care. Surgical care is delivered in 5 of the 10 wards, an accident and emergency department with a trauma ward for short stay (< 24hrs) emergency surgical patients, an intensive care unit, five operating theatres and a surgical outpatient unit. The surgical department is run by 8 surgical and 2 anaesthetic consultants covering six specialities: general surgery, surgical oncology, urology, paediatrics, trauma and orthopaedics, and ENT. The average surgical volume is 80 -100 operations per month.[11]

Participants

Participants were all surgical patients who consented to take part, receiving operative or non-operative surgical care under the care of the hospital surgical team and located on one of the surgical wards. Patients under the care of non-surgical teams; patients under the age of 16 who were without a parent, guardian, or head of the household; and participants unable to consent and/or unwilling to take part in the study were excluded. Participants were recruited consecutively to the study on admission for surgical care from June to August 2018.

Data collection

A structured questionnaire was administered to patients and/or their relatives at the time of formal discharge from surgical care while patients were on the ward. Where patients self-discharged or left against medical advice, where possible they were interviewed when leaving the hospital. Interviews were conducted in a private space and all participants were encouraged to bring a relative, head of the household, or the main breadwinner to allow for expenditure and OOP costs to be captured accurately.

The questionnaire was designed based on tools used in similar studies done in LMIC settings.[16-19] It was co-designed with in-country experts, healthcare professionals, and researchers to ensure that the questions were suitable for the SL context. The questionnaire was pilot-tested for ease of comprehension, clarity of definitions, appropriateness of questions, and manageability of the length of the interview in six patients (who were excluded from the analysis). Minor modifications were made to the wording of the questions based on this, but the meaning of the questions was not changed. The questionnaire was designed and written in English and administered by trained Sierra Leonean research assistants (RAs) in either English or a chosen local dialect (most commonly Krio). Data was captured on paper and later transferred to electronic format.

Definition and construction of variables

Data was collected on the age and gender of the participant, and their address (later used to calculate if they were resident in an urban or rural area). The occupation of the main breadwinner was recorded using both a free text answer stating the occupation, followed by a question on whether this was salaried (i.e. employed) or non-salaried (i.e. self-employed or working in the informal sector). Education was captured as the highest level of education of the main breadwinner using the following categories: no formal education, primary, secondary, college or university level, or other. Information on household expenditure was captured by asking 7 questions on regular items purchased in a typical week (food and drink etc.), 11 questions on larger expenditure items typically purchased monthly (toiletries, clothing, etc.) and a further 12 questions on typical yearly spend on big household items such as furniture and livestock. Total food expenditure ($foodexp_h$) was summed as a separate variable for the purposes of calculating catastrophic expenditure (where food expenditure was used to define subsistence costs). Number of people living in the household (*HHsize)* was also captured, as was the

For peer review only - http://bmjopen.bmj.com/site/about/guidelines.xhtml

number of days of sickness before presentation, whether care had been sought elsewhere prior to presentation at Connaught Hospital, and the mode of transport used.

Data was also collected on the following: whether the patient was an emergency or elective case; whether or not the participant was eligible for free healthcare (for patients under the age of 5 years old, pregnant or lactating mothers, Ebola survivors, destitute and disabled patients); and the primary diagnosis, recorded from review of the patient's admission notes, ward and theatre ledgers (later summarised into 10 categories of surgical conditions: trauma, hernia, abdominal conditions, peripheral vascular disease or diabetic foot disease, urological conditions, breast mass / cancer, burns, ENT / dental disease, thyroid, congenital abnormality, or paediatrics). Treatment was categorised as operative or non-operative following review of the patient's admission notes. Length of hospital stay was also calculated.

Direct medical OOP costs were captured across the entire illness episode including in-hospital costs (from the point of admission to discharge from the tertiary care hospital) and pre-hospital costs (for other medical costs related to the admission episode which occurred prior to the tertiary care admission). In-hospital direct medical costs were the sum of administrative costs (including registration, admission, triage, bed and discharge fees), medications, medical supplies, investigations, blood transfusion, operation cost, and informal payments (defined as any payment that was not part of hospital policy, such as doctors' fees, tips, payments made to porters and to nursing staff for nursing care). If costs were 'formal', we asked whether these costs were paid directly to the hospital bank / cashiers directly or via hospital staff, or to an external facility (such as external pharmacy or laboratory). For pre-hospital care, non-medical direct costs were calculated from transport costs. For the hospital episode, non-medical direct costs were captured as: cost of transport to the hospital or to and from the hospital to get food, medical supplies and investigations from external facilities, and the cost of food and accommodation during the hospital stay. Finally, indirect costs were captured by estimating lost wages during the illness episode.

All costs are presented in Le and $US at the conversion rate of 15th July 2019 (1 Sierra Leonean Leone = 0.00011567 USD).

Total household expenditure (*totalexp*$_h$) was calculated over the course of 12 months by summing all the variables collected on all regular household items purchased as described above.

Total OOP payments for surgical care (OOP$_t$*): = total direct medical costs + total direct non-medical costs + total indirect costs*

Catastrophic expenditure (CE) was defined as either an expense of more than 40% of non-subsistence expenditure (i.e. household expenditure net of subsistence [here, food (*foodexp*$_h$)] costs) or an expense more than 10% of total annual expenditure.

CE was therefore present if: $$\frac{OOP_t}{totalexp_h - foodexp_h} > 0.4$$

Or if: $$\frac{OOP_t}{totalexp_h} > 0.1$$

Impoverishing Expenditure (IE) is defined as being pushed into or further into poverty. The Sierra Leone national poverty line (spending < $1.25/person /day) threshold was used for the main analysis. In addition, two further thresholds for poverty were used based on World Bank definitions: "poverty" - spending < $3.10/person/day and "extreme poverty" - spending <$1.90/person/day.[4] Presence of poverty before (baseline) and after OOP spending on surgical care were then calculated.

For peer review only - http://bmjopen.bmj.com/site/about/guidelines.xhtml

Baseline poverty (BLP_h) at each threshold was determined to be present if total household expenditure ($totalexp_h$) per individual inhabiting each household divided by the number of days in the year was below the poverty threshold chosen. i.e.: $\frac{\left(\frac{totalexp_h}{HHsize}\right)}{365} \leq poverty\ line$

Impoverishment as a result of surgical care was defined as present if the total household expenditure net the total OOP costs for surgical care ($totalexp_{netsurg} = totalexp_h - OOP_t$) per head of household, per day was less than the chosen poverty threshold

i.e.: IE present if $\frac{\left(\frac{totalexp_{netsurg}}{HHsize}\right)}{365} \leq poverty\ line$

Both CE and IE are presented as the number and percentage of participants who experienced CE and or IE.

Summary household asset data was collected using a yes or no response to the ownership of the following assets: Electricity / Light, Mobile phone, Radio, Television, Computer, Refrigerator, Generator, Bicycle, Motorcycle and Car or truck.

Sample size and power calculation

Based on a similar study done in Uganda which estimated CE to be 31%[16] in a free healthcare setting, modelled and WB data for SL which estimates CE at 84.7% and 49.9% respectively, and from discussion with academics with in-country knowledge, we estimated that CE would be around 60% of patients admitted for surgical care. The sample size required to capture this with a CI of 55-65%, allowing for 10% loss to follow up was 442 patients.

Statistical analysis

Statistical analysis was done using SPSS Version 25 for windows.

Characteristics of the population seeking care are described. Normally distributed data are presented as mean and standard deviation (SD), otherwise median and IQR are used. Multiple Imputation Chained Equations were used to compute missing data-points using predictive mean matching for variables with less than 20% missingness and where missingness was identified as not at random. Where imputed variables were used, the pooled mean is shown as standard SPSS output. A complete case analysis was done for variables describing how costs of accessing care were met and the consequences of accessing care.

Wealth characteristics (household asset ownership) of the population accessing surgical care were compared with those in the general population (2015 Census data[20]) using the Chi squared test.

Associations between direct medical in-hospital OOP costs of care and age, sex, type of admission (emergency or elective), operative or non-operative care, type of operative procedure, or length of stay were tested using a generalised linear model using a Tweedie function with a power of 1.9.

Ethical approval

Ethical approval was granted by the Sierra Leone Ethics and Scientific Review Committee and from the King's College London Research Ethics Committee (ref. LRU-17/18-6455)

All patients gave written consent to participate where possible and witnessed thumbprints and verbal consent where patients were illiterate. Patients were given information about the study at admission

For peer review only - http://bmjopen.bmj.com/site/about/guidelines.xhtml

and consented between 4-24 hours later after due time was given to consider the study information. Consent was re-confirmed just prior to doing the exit interview.

Results

Of the initial 416 recruited participants, a total of 335 were interviewed (Figure 1). Participant characteristics are presented in table 1. In summary, the mean age of the interviewed patients was 28 (SD 20). 39.4% were female and 80.3% lived in an urban area. 29% were formally employed with a further 66.9% being employed but without a regular salary – either self-employed or employed within the informal sector. The level of education of the main breadwinner was secondary school in 37.9%, college / university in 28% and no formal education in 23.6%. The median household size was 6 (IQR: 4) with a mean total yearly household expenditure of US$3569 (see appendix table 1 for imputed and non-imputed data and appendix table 2 for a comparison with expenditure assessed in the Economic and Financial survey in 2014). 67.2% of participants had sought care for their illness elsewhere prior to presentation at the tertiary referral hospital. 71.9% arrived by public transport and the majority were classed as emergency admissions (72.2%). The most common reasons for presentation were trauma, hernia, or other abdominal conditions. 67.5% underwent operative intervention with the remainder being managed by non-operative measures. Median length of stay was 8 days (IQR: 18).

The total mean cost for the surgical care episode was US$243 of which US$24 (10%) accounted for pre-hospital direct costs (medical costs were US$21 and non-medical were US$3). Of the in-hospital direct costs (mean US$172), a mean of US$138 (63%) was due to direct medical costs and US$34 (16%) for direct non-medical costs. Indirect costs, such as lost wages, totalled US$46. (Table 2 and appendix table 3).

Of the in-hospital direct medical costs, 48% were given to hospital staff (it was not clear whether the hospital staff later transferred these funds to the hospital bank or not), 33% were made directly to the hospital bank / cashiers and 17% to an external facility such as external pharmacy or diagnostic centre (Appendix table 4). A variety of means were used to meet costs (Table 3). Most (83.7% of patients) used their savings to meet some or all of the costs; only 2% (6 patients) had some form of health insurance. Wider implications included loss of wages in 36.9% and loss of job in 6.0%.

Catastrophic expenditure, when defined as OOP costs of more than 40% of capacity to pay affected 10% of those interviewed, rising to 18% when defined as out-of-pocket costs more than 10% of all household expenditure.

Prior to the surgical care episode, 45% of people interviewed were below the national poverty line, 90% were below the World Bank Poverty Level, and 70% below the World Bank Extreme Poverty level. Following payment for surgical care, 50% were pushed below or further below the national poverty line. Corresponding figures were 91% and 73%, for the World Bank thresholds of poverty and extreme poverty, respectively.

Analysis of the possession of household assets demonstrated that those interviewed were more likely to have electricity, a mobile phone, radio, television, refrigerator, bicycle, motorcycle or car than those of the general population in Sierra Leone (2015 Census data, all p=<0.001) or of the urban population in the Western Area (2015 Census data, all p=<0.05) (Table 4).

Regression analysis demonstrated that the factors that were significantly associated with increased costs were age, length of hospital stay, and undergoing a general surgical or urological procedure (Appendix table 5).

Discussion

For peer review only - http://bmjopen.bmj.com/site/about/guidelines.xhtml

In this study, we found that accessing and receiving tertiary level surgical care in Sierra Leone requires large up-front OOP payments which have a substantial impact on individual and households' economic situations. These equate to a catastrophic expense in nearly a fifth of households and are impoverishing half of the households that receive care. We found poverty, as assessed by household expenditure, was high indicating a limited financial buffer to accommodate costs of care. This is despite most people who access surgical care owning a higher level of assets that the general population.

The majority of the OOP payments were incurred in-hospital and as a result of direct medical costs. Payment for the operation itself and medications, medical supplies, and investigations (including laboratory tests) were the biggest contribution to these costs. A small percentage of costs were categorised as unofficial, such as for "nursing care" and "tips", although these were given by a majority of people who received care. In addition, in enquiring about the payment routes for formal costs, we identified that almost half of these were being paid through unofficial payment channels and made directly to staff. We do not know whether these payments were later transferred by staff to the hospital bank, however, that these informal routes are common indicates poor financial governance which urgently needs to be addressed.

Assessment of the wider implications of seeking surgical care in SL highlighted that the majority of payments were met using savings, followed by raising money from family contributions or borrowing money which may leave households in debt. In addition, a large number of participants lost wages during the sickness episode and a proportion lost their jobs. In a country where informal work predominates and earnings can be unpredictable, this may impact on household financial security and influence future health seeking behaviour, both of the individuals affected and their immediate family and communities.

The majority of patients accessing surgical care were young males; whether this male predominance is a true reflection of disease burden in Sierra Leone or reveals a hidden gender bias in care seeking behaviour is beyond the remit of this study. Nevertheless, males who sought care in our study are traditionally the main breadwinners and the most economically active population group in SL. This loss of wages and livelihood could have implications on the wider socio-economic determinants of health and the well-being of the household. The additional burden to the patients and their households as a result of the indirect costs supports the macroeconomic argument for investing in surgical care put forward by Grimes et al, who demonstrated the opportunity to avert 36,487 DALYs by investing in surgical care at hospital level in Sierra Leone. [21,22]

Some specialties, such as general surgery and urology incurred much higher overall costs for the surgical episode and this may be because operative intervention (with blood transfusion and a longer length of stay) is usually required. This contrasts for example, to trauma care that was often managed non-operatively. Such non-operative treatment for trauma may be partly as a result of local surgical practice, often driven by lack of resources such as the unavailability of internal fixation wires and orthopaedic implants, and partly because some common orthopaedic problems are managed non-operatively. In addition, we found that age and length of hospital stay were associated with significantly higher costs. This may be due to the fact that those under the age of 5 years were eligible for free health care in SL and that a longer stay in hospital may be associated with higher direct non-medical and indirect costs such as payment for food and lost wages.

There are a limited number of studies to draw a direct comparison with as only a few used a similar methodology (direct interview) as opposed to modelled data or the use of caesarean section costs as a proxy measure to extrapolate costs, CE and impoverishment.[2,16,23-28] There are even fewer studies that report on the financial implications of all or most types of surgical care. The majority report on

For peer review only - http://bmjopen.bmj.com/site/about/guidelines.xhtml

single surgical subspecialties such as obstetric care, paediatric surgery or trauma care. Nevertheless, there have been three recent studies from Uganda reporting CE rates of 31% and IE of 47% (assessing all surgical categories), CE of 55% (in adult surgery only) and 45% (in paediatric surgery).[16,29,30] A study in Malawi interviewing patients undergoing hernia operations reported CE rates as high as 90% using a threshold of 10% of yearly income.[26] Various studies looking at injury and trauma care costs in Vietnam, India and Nigeria have reported CE rates of 60%, 30% and 86% respectively[23] and a study in Morocco looking at obstetric surgical care alone estimated CE rates of 88%[31] while an emergency obstetric care study in Indonesia estimated CE at 68%.[32] This highlights the inter-country variability and although that makes it difficult to draw comparative conclusions, our study results relate closest to the Ugandan study that assessed all surgical categories using a similar methodology and thresholds. This highlights the need for a standardised way of assessing and measuring the financial implications of surgical care, to allow accurate collection and reporting of these global surgery metrics on financial risk protection.

In keeping with other studies, we noted lower rates of CE and IE in comparison to the modelled and extrapolated estimates for SL. This is probably because the modelled studies are based on the whole population that may require surgery and not on those that have successfully accessed surgical care. The lower rates of IE and CE seen may therefore be explained by a lack of access by the poorest. This is supported by data from SL that estimates that up to 25% of deaths in 2011 could have been averted through access to safe, timely and affordable surgical care and that SL has an unmet surgical burden of disease of 92% [10], with approximately 70% of Sierra Leoneans stating that the financial burden of OOP payments for healthcare was the biggest barrier to accessing care.[33,34] In addition, we found that those accessing tertiary level surgical care came from predominantly urban areas of SL and when compared to the wider SL population, had significantly higher asset ownership. Using the latter as a proxy of wealth or socio-economic status, this could indicate that those able to access and receive surgical care represent the relatively wealthier households of SL and therefore that the poorest and those at the highest risk of financial catastrophe are not accessing care when needed.

Limitations

There are several limitations to this study. Firstly, it was dependant on recall and self-reported estimates of OOP costs and household expenditure. Although the questionnaire and methodology are a well-established way of obtaining this information in a low-resource setting where informal work predominates and payments are not often receipted, the data is still subject to respondent and recall bias. This may have contributed to missing data or the underestimation of OOP costs and possible overestimation of household expenditure which is also subject to social desirability bias.

Attempts made to minimise this included breaking down household expenditure questions to weekly, monthly and yearly costs, using a chronological approach to the OOP cost questions that helped map out the patients journey for them, encouraging participants to bring an appropriate family member to the interview, and by gaining in-country consensus and piloting the questionnaire prior to use.

Whilst attempts were made to ensure another family member or respondent was available during the interview to assist with accuracy, this was not always possible. In addition, given the reliance on savings, borrowed money and family contributions, payments were often made without the knowledge of the patient and were therefore difficult to reliably collect. As a result, the data was handled using an established statistical approach that aimed to accurately account for the missing values. Further comparison between non-imputed and imputed data was performed which showed minimal disparity suggesting reliability of this method (see Appendix).

Secondly, given the informal payment methods and informal costs, collection of this information in-hospital and often on the ward may have prevented patients from honestly declaring all costs, leading

For peer review only - http://bmjopen.bmj.com/site/about/guidelines.xhtml

to underreporting. However, analysis of the missing data did not show a correlation or bias towards more missing costs if paid directly to staff versus at the bank.

In addition, the study only measures costs incurred during the illness episode up until discharge and therefore does not capture the indirect costs if patients are off work or do not return back to work following discharge or the costs of ongoing outpatient or follow-up care, medications and medical supplies. We have therefore likely substantially underestimated the total costs of seeking surgical care.

Finally, the desired sample size was not achieved. Nevertheless, although sample size was not obtained, the 95% confidence interval for a catastrophic expenditure rate of 18% was 14-22% which gives the study an overall power of 90%.

Conclusion

This is the first empirical study from Sierra Leone that quantifies the financial burden of accessing and receiving surgical care. It adds insight into the global and national SL modelled estimates of the likelihood of catastrophic and impoverishing expenditure if surgery is required and joins the small but growing body of other empirical studies reporting on the OOP costs and wider financial implications of surgical care. In addition, it highlights the need to prioritise financial risk protection within healthcare and surgery if universal health coverage is to be achieved.

References

1. Meara JG, Leather AJ, Hagander L, et al. Global Surgery 2030: evidence and solutions for achieving health, welfare, and economic development. *Lancet* 2015; **386**(9993): 569-624.
2. Shrime MG, Dare AJ, Alkire BC, O'Neill K, Meara JG. Catastrophic expenditure to pay for surgery worldwide: a modelling study. *Lancet Glob Health* 2015; **3 Suppl 2**: S38-44.
3. Shrime MG, Bickler SW, Alkire BC, Mock C. Global burden of surgical disease: an estimation from the provider perspective. *Lancet Glob Health* 2015; **3 Suppl 2**: S8-9.
4. World Bank Development Indicators 2016. http://data.worldbank.org/data-catalog/world-development-indicators (accessed 10th August 2018.
5. Shrime MG, Dare A, Alkire BC, Meara JG. A global country-level comparison of the financial burden of surgery. *Br J Surg* 2016; **103**(11): 1453-61.
6. WHO. World Health Organisation: Health statistics and information systems. Global Reference List of 100 Core Health Indicators. . 2015. http://www.who.int/healthinfo/indicators/2015/en/
7. World Bank: Risk of catastrophic expenditure for surgical care. Sierra Leone https://data.worldbank.org/indicator/SH.SGR.CRSK.ZS?locations=SL.
8. Farmer PE, Kim JY. Surgery and global health: a view from beyond the OR. *World J Surg* 2008; **32**(4): 533-6.
9. Bolkan HA, Von Schreeb J, Samai MM, et al. Met and unmet needs for surgery in Sierra Leone: A comprehensive, retrospective, countrywide survey from all health care facilities performing operations in 2012. *Surgery* 2015; **157**(6): 992-1001.
10. Groen RS, Samai M, Stewart KA, et al. Untreated surgical conditions in Sierra Leone: a cluster randomised, cross-sectional, countrywide survey. *Lancet* 2012; **380**(9847): 1082-7.
11. National Surgical, Obstetric and Anaesthesia Strategic Plan. . Sierra Leone Ministry of Health and Sanitation. .
12. Hsu J, Flores G, Evans D, Mills A, Hanson K. Measuring financial protection against catastrophic health expenditures: methodological challenges for global monitoring. *Int J Equity Health* 2018; **17**(1): 69.

For peer review only - http://bmjopen.bmj.com/site/about/guidelines.xhtml

For peer review only

13. Xu K, Evans DB, Carrin G, Aguilar-Rivera AM, Musgrove P, Evans T. Protecting households from catastrophic health spending. *Health Aff (Millwood)* 2007; **26**(4): 972-83.
14. Hoang HT, Pham TL, Vo TT, Nguyen PK, Doran CM, Hill PS. The costs of traumatic brain injury due to motorcycle accidents in Hanoi, Vietnam. *Cost Eff Resour Alloc* 2008; **6**: 17.
15. Nguyen H, Ivers R, Jan S, Martiniuk A, Pham C. Catastrophic household costs due to injury in Vietnam. *Injury* 2013; **44**(5): 684-90.
16. Anderson GA, Ilcisin L, Kayima P, et al. Out-of-pocket payment for surgery in Uganda: The rate of impoverishing and catastrophic expenditure at a government hospital. *PLoS One* 2017; **12**(10): e0187293.
17. World Health Organisation. Health statistics and information systems. Data collection tools - World Health Survey. . http://www.who.int/healthinfo/survey/en/ (accessed 19th April 2018).
18. (SAGE). WHOHsaisDct-WSoGAaAH. http://www.who.int/healthinfo/sage/en/
19. Grimes CE, Billingsley ML, Dare AJ, et al. The demographics of patients affected by surgical disease in district hospitals in two sub-Saharan African countries: a retrospective descriptive analysis. *Springerplus* 2015; **4**: 750.
20. 2015 Population and House Census: Summary of Final Results. : Statistics Sierra Leone
21. Grimes CE, Quaife M, Kamara TB, Lavy CBD, Leather AJM, Bolkan HA. Macroeconomic costs of the unmet burden of surgical disease in Sierra Leone: a retrospective economic analysis. *BMJ Open* 2018; **8**(3): e017824.
22. Grimes CE, Law R, Dare A, et al. Cost-Effectiveness of Two Government District Hospitals in Sub-Saharan Africa. *World J Surg* 2017; **41**(9): 2187-92.
23. Nguyen H, Ivers R, Jan S, Pham C. Cost of surgery and catastrophic expenditure in people admitted to hospital for injuries: estimates from a cohort study in Vietnam. *Lancet* 2015; **385 Suppl 2**: S50.
24. Prinja S, Bahuguna P, Gupta R, Sharma A, Rana SK, Kumar R. Coverage and Financial Risk Protection for Institutional Delivery: How Universal Is Provision of Maternal Health Care in India? *PLoS One* 2015; **10**(9): e0137315.
25. Honda A, Randaoharison PG, Matsui M. Affordability of emergency obstetric and neonatal care at public hospitals in Madagascar. *Reprod Health Matters* 2011; **19**(37): 10-20.
26. Bijlmakers L, Wientjes M, Mwapasa G, et al. Out-of-pocket payments and catastrophic household expenditure to access essential surgery in Malawi - A cross-sectional patient survey. *Ann Med Surg (Lond)* 2019; **43**: 85-90.
27. Banu T, Chowdhury TK, Aziz TT, et al. Cost Incurred by the Family for Surgery in Their Children: A Bangladesh Perspective. *World J Surg* 2018; **42**(12): 3841-8.
28. Urua U, Osungbade K, Obembe T, Adeniji F. A cost analysis of road traffic injuries in a tertiary hospital in south-west Nigeria. *Int J Inj Contr Saf Promot* 2017; **24**(4): 510-8.
29. MacKinnon N, St-Louis E, Yousef Y, Situma M, Poenaru D. Out-of-Pocket and Catastrophic Expenses Incurred by Seeking Pediatric and Adult Surgical Care at a Public, Tertiary Care Centre in Uganda. *World J Surg* 2018; **42**(11): 3520-7.
30. Yap A, Cheung M, Kakembo N, et al. From Procedure to Poverty: Out-of-Pocket and Catastrophic Expenditure for Pediatric Surgery in Uganda. *J Surg Res* 2018; **232**: 484-91.
31. Boukhalfa C, Abouchadi S, Cunden N, Witter S. The free delivery and caesarean policy in Morocco: how much do households still pay? *Trop Med Int Health* 2016; **21**(2): 245-52.
32. Quayyum Z, Nadjib M, Ensor T, Sucahya PK. Expenditure on obstetric care and the protective effect of insurance on the poor: lessons from two Indonesian districts. *Health Policy Plan* 2010; **25**(3): 237-47.
33. Ponsar F, Tayler-Smith K, Philips M, et al. No cash, no care: how user fees endanger health--lessons learnt regarding financial barriers to healthcare services in Burundi, Sierra Leone, Democratic Republic of Congo, Chad, Haiti and Mali. *Int Health* 2011; **3**(2): 91-100.
34. Xu K, Evans DB, Kawabata K, Zeramdini R, Klavus J, Murray CJ. Household catastrophic health expenditure: a multicountry analysis. *Lancet* 2003; **362**(9378): 111-7.

For peer review only - http://bmjopen.bmj.com/site/about/guidelines.xhtml

For peer review only

Figures and tables

Figure 1

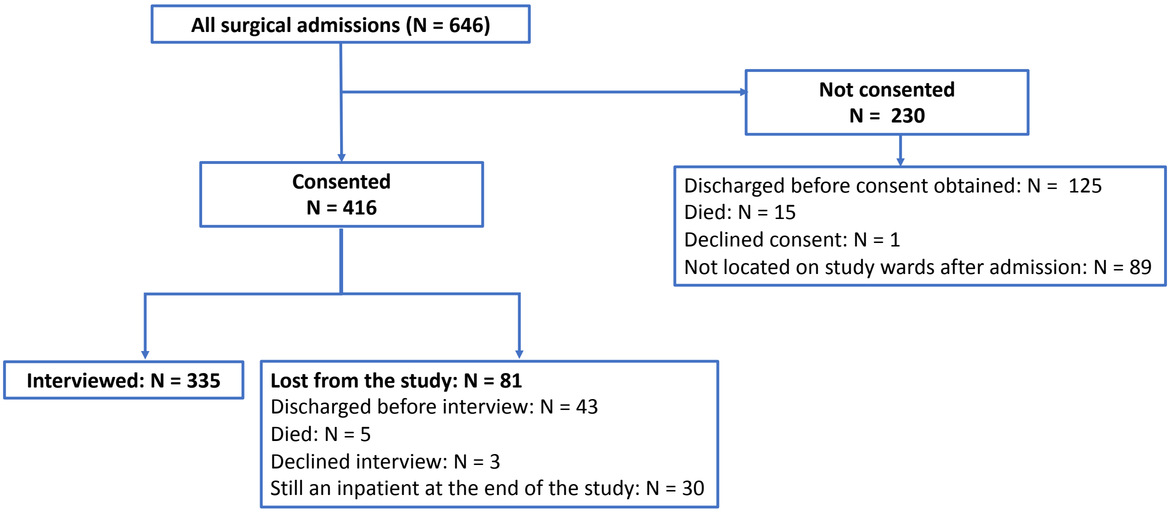

Figure 1: Study recruitment process diagram. Flow diagram of the total number of admissions (646) during the study period, highlighting those recruited, lost from the study and reasons for this.

For peer review only - http://bmjopen.bmj.com/site/about/guidelines.xhtml

Tables

Table 1: Participant characteristics

**Demographics of participants**	
Total number of patients interviewed	335
Mean age in years (SD)	28 (20)
Female number (%)	132 (39.4%)
Urban Residents (%)	269 (80.3%)
**Type of job (number (%)):**	
Self Employed / Informal Sector	224 (66.9%)
Employed	97 (29%)
Unemployed / Retired	12 (3.6%)
Missing /Don't know	2 (0.6%)
**Level of education of main breadwinner (number (%)):**	
No formal education	79 (23.6%)
Primary school	25 (7.5%)
Secondary school	127 (37.9%)
College / University	94 (28%)
Other / Missing / Don't know	10 (3.0%)
Median household size (IQR)	6 (4)
Total yearly household expenditure (US$)	$ 3,569
Number below national poverty line prior to illness	151 (45%)
**Surgical Care Episode Descriptors**	
Median days of sickness before presentation (IQR)	2 (7)
Number that sought care for illness elsewhere prior to presentation at Connaught	225 (67.2%)
**Mode of transport used to travel to hospital (number (%)):**	
Public transport	241 (71.9%)
Ambulance	67 (20%)
Private transport	23 (6.9%)
Walked	3 (0.9%)
Don't know / Missing	1 (0.3%)
Emergency admission (%)	242 (72.2%)
Eligible for free health care (%)	70 (20.9%)
**Primary diagnosis by surgical condition (number (%))**	
Trauma	114 (34%)
Hernia	58 (17.3%)
Abdominal conditions	56 (16.7%)
Peripheral vascular disease or diabetic foot disease	27 (8.1%)
Urological conditions	23 (6.9%)
Breast mass / cancer	16 (4.8%)
Burns	15 (4.5%)
ENT / dental disease	13 (3.9%)
Goitre	7 (2.1)
Congenital abnormality (paediatrics)	3 (0.9%)
Missing / don't know	3 (0.9%)
**Treatment (number (%)):**	
Operative	226 (67.5%)
Non-operative	109 (32.5%)
Median length of hospital stay (LOS) in days (IQR)	8 (18)

For peer review only

Table 2: Out-of-pocket costs.

Costs	Imputed mean cost ($US)
**Prehospital costs**	
**Direct pre-hospital medical OOP costs (total)**	**21 (88% of 24)**
- Consultation	2 (10% of 21)
- Medications	12 (57% of 21)
- Medical supplies	2 (10% of 21)
- Investigations	4 (19% of 21)
- Other miscellaneous	2 (10% of 21)
**Direct (pre-hospital) non-medical OOP costs (total)**	**3 (13% of 24)**
- Transport	3 (100% of 3)
Total pre-hospital costs	***24 (10% of 243)***
**In hospital costs**	
**Direct medical OOP costs (total)**	**138 (63% of 219)**
- Administrative	20 (14% of 138)
- Medications	26 (19% of 138)
- Medical supplies	14 (10% of 138)
- Investigations	15 (11% of 138)
- Blood transfusion	9 (7% of 138)
- Total operation costs	49 (36% of 138)
- Unofficial costs	6 (4% of 138)
- Other / miscellaneous	1 (1% of 138)
**Direct non-medical costs (total)**	**34 (16% of 219)**
- Transport to hospital	7 (21% of 34)
- Food	20 (59% of 34)
- Accommodation	0 (0% of 34)
- Other*	7 (21% of 34)
**Indirect costs**	
- Lost wages	46 (100% of 46)
**TOTAL OOP COSTS**	**243**

*other relates to travel and other associated costs incurred as a result for needed investigations from and or medication / supplies from an external facility. SPSS calculates only the mean using imputed variables, hence no standard deviation is displayed.

For peer review only - http://bmjopen.bmj.com/site/about/guidelines.xhtml

Table 3: How costs are met and the wider implications of seeking and undergoing surgical care (n is the number of cases with data on each variable)

How costs were met (total number responding to question)	Number (%) that used this as a means of meet OOP costs
Used **Savings** (n=326)	**273 (83.7%)**
Arranged **family contributions** (n=331)	**128 (38.7%)**
**Borrowed** money (n=331)	**102 (30.8%)**
Received **charity** money (n = 331)	**83 (25.3%)**
**Sold possessions** (n=329)	**17 (5.2%)**
**Other** (n=331)	**14 (4.2%)**
**Pawned** possessions (n=332)	**8 (2.4%)**
Have **Health insurance** (n=335)	**6 (1.8%)**
**Wider implications**	**Number (%) that experienced the wider implications of meeting OOP costs**
**Loss of wages** (n = 328)	**121 (36.9%)**
**Lost their job** / changed their role at work / home (n = 331)	**20 (6.0%)**
**Disruption to education** (n = 333)	**12 (3.6%)**

Table 4: Ownership of household assets in comparison to 2015 census data

Household assets	**Surgical cohort** Number (%) of households that own the asset	**2015 Census data** Whole country data
**Electricity**	227 (67.8%)	17.8%
**Mobile phone**	326 (97.3%)	62.94%
**Radio**	280 (83.6%)	58.03%
**Television**	212 (63.3%)	19.76%
**Refrigerator**	119 (35.5%)	8.22%
**Bicycle**	38 (11.3%)	6.43%
**Motorcycle**	8 (14.3%)	7.62%
**Car**	50 (14.9%)	3.65%

For peer review only - http://bmjopen.bmj.com/site/about/guidelines.xhtml

Appendix

Appendix table 1: Household expenditure showing imputed and non-imputed data sets.
Comparison of non-imputed and imputed data on household expenditure using Multiple Imputation Chained Equations to compute missing data-points using predictive mean matching.

Household expense	Non-imputed data mean (SD) (Le and $US)	Imputed data pooled mean (Le and $ US)
**Individual Consumption Expenditure by Households Including all variables collected**	**Le 39,665,597 (53,679,740) $ 4,425 (5,989)**	**Le 47,944,384 $5,349**
**Individual Consumption Expenditure by Households Excluding variables with > 20% missing data i.e. clothing, mobile phone credit and transport**	**Le 28,134,505 (31,539,987) $ 3,139 (3,519)**	**Le 31,988,507 $ 3,569**
Food and non-alcoholic beverages	Le 18,616,404 (22,364,391) $ 2,077 (2,495)	Le 20,867,118 $ 2,328
Alcoholic Beverages, Tobacco and Narcotics	Le 252,991 (1,047,612) $ 28 (117)	Le 314,095 $ 35
Rental	Le 884,123 (4,670,009) $ 99 (521)	Le 876,940 $ 98
Household maintenance	Le 108,092 (298,466) $ 12 (33)	Le 130,857 $ 15
Electricity, gas and other fuels	Le 720,748 (1,068,694) $ 80 (119)	Le 747,701 $ 83
Furnishings, household equipment and routine household maintenance	Le 632,577 (1,065,761) $ 71 (119)	Le 699,188 $ 78
Healthcare (traditional and western medicine)	Le 561,770 (1,490,207) $ 63 (166)	Le 740,205 $ 83
Recreation and cultural services	Le 821,684 (1,894,677) $ 92 (211)	Le 1,121,965 $ 125
Education	Le 1,338,183 (2,295,578) $ 149 (256)	Le 1,614,982 $ 180
Personal care / toiletries	Le 609,012 (629,656) $ 68 (70)	Le 627,269 $ 70
Health insurance	Le 8,333 (138,661) $ 1 (15)	Le 13,370 $ 1
Remittance	Le 974,408 (1,655,020) $ 109 (185)	Le 1,015,363 $ 113
Donations	Le 185,233 (475,436) $ 21 (53)	Le 203,246 $ 23
Livestock	Le 41,400	Le 45,254

For peer review only - http://bmjopen.bmj.com/site/about/guidelines.xhtml

	(213,804)<br>$ 5 (24)	$5
Taxes	Le 59,500<br>(261,497)<br>$ 7 (29)	Le 73,723<br><br>$ 8

Appendix table 2: Household expenditure data. Variables on household expenditure shown here, for comparison, with the Economic and Financial survey SL 2014 data. Categories were harmonised where possible, however given differences in questions asked between surveys, an exact match of categories was not possible to achieve.

**Household consumption expenditure (in Leones (Le) and USD ($))**		
**Household Expense**	**Sierra Leone Economic and Financial Survey data 2014**	**Study data**
**Individual Consumption Expenditure by Households (Total Expenditure)**	**Le 15,414,816<br>$ 1,739**	**Le 31,988,507<br>$ 3,569**
**Food and non-alcoholic beverages**	**Le 6,838,365<br>$ 771**	**Le 20,867,118<br>$ 2,328**
Food	Le 6,644,019<br>$ 74	Le 17,925,090<br>$ 2,000
Non-alcoholic beverages	Le 194,346<br>$ 22	Le 2,942,028<br>$ 328
**Alcoholic Beverages, Tobacco and Narcotics**	**Le 450,612<br>$ 51**	**Le 314,095<br>$ 35**
**Housing, water, electricity, gas and other fuels**	**Le 1,058,449<br>$119**	**Le 875,672<br>$ 98**
**Rental**	**Le 253,948<br>$ 29**	**Le 876,940<br>$ 98**
**Maintenance and repair of the dwelling**	**Le 32,650<br>$ 4**	**Le 876,940<br>$ 98**
**Electricity, gas and other fuels**	**Le 595,832<br>$ 67**	**Le 747,701<br>$ 83**
**Furnishings, household equipment and routine household maintenance**	**Le 413,364<br>$ 47**	**Le 699,188<br>$ 78**
Furnishings	Le 88,058<br>$ 10	Le 196,217<br>$ 22
Household appliances	Le 41,821<br>$ 5	Le 314,772<br>$ 35
Tools and equipment for house and garden	Le 45,403<br>$ 5	Le 57,344<br>$ 6
Goods and services for routine household maintenance	Le 105,438<br>$ 12	Le 130,857<br>$ 15
Purchase of vehicles	Le 108,710<br>$ 12	Le 160,759<br>$ 18
Educations	Le 794,478<br>$ 90	Le 1,614,982<br>$ 180
Insurance (HI)	Le 38,890<br>$ 4	Le 13,370<br>$ 1

For peer review only - http://bmjopen.bmj.com/site/about/guidelines.xhtml

Appendix table 3: Imputed and non-imputed data for out-of-pocket costs for comparison.

Costs	Imputed<br>Mean cost ($US)	Non-imputed<br>Mean cost ($US) (SD)
**Prehospital costs**		
**Direct pre-hospital medical OOP costs (total)**	**21 (88% of 24)**	**14 (65)**
- Consultation	2 (10% of 21)	1 (6)
- Medications	12 (57% of 21)	9 (46)
- Medical supplies	2 (10% of 21)	2 (8)
- Investigations	4 (19% of 21)	4 (25)
- Other miscellaneous	2 (10% of 21)	1 (10)
**Direct (pre-hospital) non-medical OOP costs (total)**	**3 (13% of 24)**	**3 (9)**
- Transport	3 (100% of 3)	3 (9)
Total pre-hospital costs	***24 (10% of 243)***	***25 (75)***
**In hospital costs**		
**Direct medical OOP costs (total)**	**138 (63% of 219)**	**109 (121)**
- Administrative	20 (14% of 138)	16 (24)
- Medications	26 (19% of 138)	25 (61)
- Medical supplies	14 (10% of 138)	11 (33)
- Investigations	15 (11% of 138)	14 (23)
- Blood transfusion	9 (7% of 138)	9 (22)
- Total operation costs	49 (36% of 138)	51 (75)
- Unofficial costs	6 (4% of 138)	5 (9)
- Other / miscellaneous	1 (1% of 138)	1 (8)
**Direct non-medical costs (total)**	**34 (16% of 219)**	**34 (34)**
- Transport to hospital	7 (21% of 34)	7 (17)
- Food	20 (59% of 34)	21 (20)
- Accommodation	0 (0% of 34)	0 (0)
- Other*	7 (21% of 34)	6 (10)
**Indirect costs**		
- Lost wages	46 (100% of 46)	35 (116)
**TOTAL OOP COSTS**	**243**	**176 (165)**

*other relates to travel and other associated costs incurred as a result for needed investigations from and or medication / supplies from an external facility. SPSS calculates only the mean using imputed variables, hence no standard deviation is displayed.

Appendix table 4: Route of payment for OOP costs; percentage of the total OOP costs by cost categories paid to bank / cashier, directly to staff or externally for different services accessed once tertiary level care was reached

Costs	% paid to Hospital bank / cashier	% paid directly to or via staff	% paid externally	% unknown	Total
**TOTAL in-hospital costs**	**32.64%**	**48.16%**	**16.50%**	**2.70%**	**100%**
**Administration**	**52.03%**	**42.53%**	-	5.44%	100%
- Registration fees	90.98%	8.71%	-	0.30%	100%
- Admission fees	66.78%	32.39%	-	0.83%	100%
- Triage fees	8.05%	91.95%	-	0.00%	100%
- Bed fees	19.47%	46.13%	-	34.40%	100%

For peer review only

- Discharge fees	41.19%	57.28%	-	1.53%	100%
**Investigations**	**19.39%**	**44.29%**	**33.73%**	**2.59%**	**100%**
- Laboratory	25.68%	40.29%	30.13%	3.90%	100%
- Imaging	14.43%	47.44%	36.57%	1.56%	100%
**Total operation costs**	**55.40%**	**32.89%**	**9.35%**	**2.35%**	**100%**
- Operation	80.10%	18.91%	-	1.00%	100%
- Medical supplies for the operation	13.42%	56.59%	25.31%	4.68%	100%
- Other / miscellaneous	69.77%	30.23%	-	0.00%	100%
**Blood transfusion**	**16.24%**	**67.68%**	**13.85%**	**2.23%**	**100%**
**Total medications and medical supplies for ward care**	**4.82%**	**61.00%**	**31.73%**	**2.45%**	**100%**
- Medications	2.19%	62.86%	31.91%	3.03%	100%
- Medical supplies	10.81%	56.74%	31.31%	1.14%	100%
**Informal payment**	**2.44%**	**97.56%**	**-**	**0.00%**	**100%**
- Doctors' fees	8.72%	91.28%	-	0.00%	100%
- Nursing care	0%	100%	-	0.00%	100%
- Porters	0%	100%	-	0.00%	100%
- Tips	0%	100%	-	0.00%	100%
**Other / miscellaneous costs**	**0%**	**98.37%**	**0**	**1.63%**	**100%**

For peer review only

Appendix table 5: Linear regression analysis showing odds of increasing in-hospital costs of care for each variable using a GLM with imputed variables using a Tweedie 1.9 function.

Variable		Odds Ratio	95% CI	p-value
Sex	Female	ref		
	Male	1.05	(0.86-1.29)	0.63
Age	Age	1.02	(1.01-1.02)	0.00
Length of stay	Length of stay	1.02	(1.02 -1.03)	0.00
Type of admission	Elective admission	ref		
	Emergency admission	0.96	(0.75-1.24)	0.77
Category of operation	Non-operative	ref		
	Burns	1.33	(0.25-7.00)	0.74
	ENT	0.64	(0.36-1.166)	0.14
	General surgery	1.67	(1.29-2.17)	0.00
	General paediatric surgery	0.84	(0.57-1.24)	0.38
	Trauma and orthopaedic	1.30	(0.98- 1.74)	0.07
	Urology	2.08	(1.22-3.53)	0.01
Area of residence	Rural	ref		
	Urban	0.98	(0.76-1.25)	0.85

For peer review only

For peer review only - http://bmjopen.bmj.com/site/about/guidelines.xhtml

For peer review only

For peer review only - http://bmjopen.bmj.com/site/about/guidelines.xhtml

Appendix

Appendix table 1: Household expenditure showing imputed and non-imputed data sets.
Comparison of non-imputed and imputed data on household expenditure using Multiple Imputation Chained Equations to compute missing data-points using predictive mean matching.

Household expense	Non-imputed data mean (SD) (Le and $US)	Imputed data pooled mean (Le and $ US)
**Individual Consumption Expenditure by Households Including all variables collected**	**Le 39,665,597 (53,679,740) $ 4,425 (5,989)**	**Le 47,944,384 $5,349**
**Individual Consumption Expenditure by Households Excluding variables with > 20% missing data i.e. clothing, mobile phone credit and transport**	**Le 28,134,505 (31,539,987) $ 3,139 (3,519)**	**Le 31,988,507 $ 3,569**
Food and non-alcoholic beverages	Le 18,616,404 (22,364,391) $ 2,077 (2,495)	Le 20,867,118 $ 2,328
Alcoholic Beverages, Tobacco and Narcotics	Le 252,991 (1,047,612) $ 28 (117)	Le 314,095 $ 35
Rental	Le 884,123 (4,670,009) $ 99 (521)	Le 876,940 $ 98
Household maintenance	Le 108,092 (298,466) $ 12 (33)	Le 130,857 $ 15
Electricity, gas and other fuels	Le 720,748 (1,068,694) $ 80 (119)	Le 747,701 $ 83
Furnishings, household equipment and routine household maintenance	Le 632,577 (1,065,761) $ 71 (119)	Le 699,188 $ 78
Healthcare (traditional and western medicine)	Le 561,770 (1,490,207) $ 63 (166)	Le 740,205 $ 83
Recreation and cultural services	Le 821,684 (1,894,677) $ 92 (211)	Le 1,121,965 $ 125
Education	Le 1,338,183 (2,295,578) $ 149 (256)	Le 1,614,982 $ 180
Personal care / toiletries	Le 609,012 (629,656) $ 68 (70)	Le 627,269 $ 70
Health insurance	Le 8,333 (138,661) $ 1 (15)	Le 13,370 $ 1
Remittance	Le 974,408 (1,655,020) $ 109 (185)	Le 1,015,363 $ 113
Donations	Le 185,233 (475,436) $ 21 (53)	Le 203,246 $ 23
Livestock	Le 41,400 (213,804)	Le 45,254 $5

For peer review only

	$ 5 (24)	
Taxes	Le 59,500<br>(261,497)<br>$ 7 (29)	Le 73,723<br><br>$ 8

For peer review only - http://bmjopen.bmj.com/site/about/guidelines.xhtml

Appendix table 2: Household expenditure data. Variables on household expenditure shown here, for comparison, with the Economic and Financial survey SL 2014 data. Categories were harmonised where possible, however given differences in questions asked between surveys, an exact match of categories was not possible to achieve.

**Household consumption expenditure (in Leones (Le) and USD ($))**		
**Household Expense**	**Sierra Leone Economic and Financial Survey data 2014**	**Study data**
**Individual Consumption Expenditure by Households (Total Expenditure)**	**Le 15,414,816<br>$ 1,739**	**Le 31,988,507<br>$ 3,569**
**Food and non-alcoholic beverages**	**Le 6,838,365<br>$ 771**	**Le 20,867,118<br>$ 2,328**
Food	Le 6,644,019<br>$ 74	Le 17,925,090<br>$ 2,000
Non-alcoholic beverages	Le 194,346<br>$ 22	Le 2,942,028<br>$ 328
**Alcoholic Beverages, Tobacco and Narcotics**	**Le 450,612<br>$ 51**	**Le 314,095<br>$ 35**
**Housing, water, electricity, gas and other fuels**	**Le 1,058,449<br>$119**	**Le 875,672<br>$ 98**
**Rental**	**Le 253,948<br>$ 29**	**Le 876,940<br>$ 98**
**Maintenance and repair of the dwelling**	**Le 32,650<br>$ 4**	**Le 876,940<br>$ 98**
**Electricity, gas and other fuels**	**Le 595,832<br>$ 67**	**Le 747,701<br>$ 83**
**Furnishings, household equipment and routine household maintenance**	**Le 413,364<br>$ 47**	**Le 699,188<br>$ 78**
Furnishings	Le 88,058<br>$ 10	Le 196,217<br>$ 22
Household appliances	Le 41,821<br>$ 5	Le 314,772<br>$ 35
Tools and equipment for house and garden	Le 45,403<br>$ 5	Le 57,344<br>$ 6
Goods and services for routine household maintenance	Le 105,438<br>$ 12	Le 130,857<br>$ 15
Purchase of vehicles	Le 108,710<br>$ 12	Le 160,759<br>$ 18
Educations	Le 794,478<br>$ 90	Le 1,614,982<br>$ 180
Insurance (HI)	Le 38,890<br>$ 4	Le 13,370<br>$ 1

For peer review only - http://bmjopen.bmj.com/site/about/guidelines.xhtml

Appendix table 3: Imputed and non-imputed data for out-of-pocket costs for comparison.

Costs	Imputed Mean cost ($US)	Non-imputed Mean cost ($US) (SD)
**Prehospital costs**		
**Direct pre-hospital medical OOP costs (total)**	**21 (88% of 24)**	**14 (65)**
- Consultation	2 (10% of 21)	1 (6)
- Medications	12 (57% of 21)	9 (46)
- Medical supplies	2 (10% of 21)	2 (8)
- Investigations	4 (19% of 21)	4 (25)
- Other miscellaneous	2 (10% of 21)	1 (10)
**Direct (pre-hospital) non-medical OOP costs (total)**	**3 (13% of 24)**	**3 (9)**
- Transport	3 (100% of 3)	3 (9)
Total pre-hospital costs	***24 (10% of 243)***	***25 (75)***
**In hospital costs**		
**Direct medical OOP costs (total)**	**138 (63% of 219)**	**109 (121)**
- Administrative	20 (14% of 138)	16 (24)
- Medications	26 (19% of 138)	25 (61)
- Medical supplies	14 (10% of 138)	11 (33)
- Investigations	15 (11% of 138)	14 (23)
- Blood transfusion	9 (7% of 138)	9 (22)
- Total operation costs	49 (36% of 138)	51 (75)
- Unofficial costs	6 (4% of 138)	5 (9)
- Other / miscellaneous	1 (1% of 138)	1 (8)
**Direct non-medical costs (total)**	**34 (16% of 219)**	**34 (34)**
- Transport to hospital	7 (21% of 34)	7 (17)
- Food	20 (59% of 34)	21 (20)
- Accommodation	0 (0% of 34)	0 (0)
- Other*	7 (21% of 34)	6 (10)
**Indirect costs**		
- Lost wages	46 (100% of 46)	35 (116)
**TOTAL OOP COSTS**	**243**	**176 (165)**

*other relates to travel and other associated costs incurred as a result for needed investigations from and or medication / supplies from an external facility. SPSS calculates only the mean using imputed variables, hence no standard deviation is displayed.

For peer review only - http://bmjopen.bmj.com/site/about/guidelines.xhtml

Appendix table 4: Route of payment for OOP costs; percentage of the total OOP costs by cost categories paid to bank / cashier, directly to staff or externally for different services accessed once tertiary level care was reached

Costs	% paid to Hospital bank / cashier	% paid directly to or via staff	% paid externally	% unknown	Total
**TOTAL in-hospital costs**	**32.64%**	**48.16%**	**16.50%**	**2.70%**	**100%**
**Administration**	**52.03%**	**42.53%**	-	5.44%	100%
- Registration fees	90.98%	8.71%	-	0.30%	100%
- Admission fees	66.78%	32.39%	-	0.83%	100%
- Triage fees	8.05%	91.95%	-	0.00%	100%
- Bed fees	19.47%	46.13%	-	34.40%	100%
- Discharge fees	41.19%	57.28%	-	1.53%	100%
**Investigations**	**19.39%**	**44.29%**	**33.73%**	**2.59%**	**100%**
- Laboratory	25.68%	40.29%	30.13%	3.90%	100%
- Imaging	14.43%	47.44%	36.57%	1.56%	100%
**Total operation costs**	**55.40%**	**32.89%**	**9.35%**	**2.35%**	**100%**
- Operation	80.10%	18.91%	-	1.00%	100%
- Medical supplies for the operation	13.42%	56.59%	25.31%	4.68%	100%
- Other / miscellaneous	69.77%	30.23%	-	0.00%	100%
**Blood transfusion**	**16.24%**	**67.68%**	**13.85%**	**2.23%**	**100%**
**Total medications and medical supplies for ward care**	**4.82%**	**61.00%**	**31.73%**	**2.45%**	**100%**
- Medications	2.19%	62.86%	31.91%	3.03%	100%
- Medical supplies	10.81%	56.74%	31.31%	1.14%	100%
**Informal payment**	**2.44%**	**97.56%**	**-**	**0.00%**	**100%**
- Doctors' fees	8.72%	91.28%	-	0.00%	100%
- Nursing care	0%	100%	-	0.00%	100%
- Porters	0%	100%	-	0.00%	100%
- Tips	0%	100%	-	0.00%	100%
**Other / miscellaneous costs**	**0%**	**98.37%**	**0**	**1.63%**	**100%**

For peer review only - http://bmjopen.bmj.com/site/about/guidelines.xhtml

Appendix table 5: Linear regression analysis showing odds of increasing in-hospital costs of care for each variable using a GLM with imputed variables using a Tweedie 1.9 function.

Variable		Odds Ratio	95% CI	p-value
Sex	Female	ref		
	Male	1.05	(0.86-1.29)	0.63
Age	Age	1.02	(1.01-1.02)	0.00
Length of stay	Length of stay	1.02	(1.02 -1.03)	0.00
Type of admission	Elective admission	ref		
	Emergency admission	0.96	(0.75-1.24)	0.77
Category of operation	Non-operative	ref		
	Burns	1.33	(0.25-7.00)	0.74
	ENT	0.64	(0.36-1.166)	0.14
	General surgery	1.67	(1.29-2.17)	0.00
	General paediatric surgery	0.84	(0.57-1.24)	0.38
	Trauma and orthopaedic	1.30	(0.98- 1.74)	0.07
	Urology	2.08	(1.22-3.53)	0.01
Area of residence	Rural	ref		
	Urban	0.98	(0.76-1.25)	0.85

For peer review only

For peer review only - http://bmjopen.bmj.com/site/about/guidelines.xhtml

BMJ Open

What is the financial burden to patients of accessing surgical care in Sierra Leone? A cross-sectional survey of catastrophic and impoverishing expenditure

Journal:	*BMJ Open*
Manuscript ID	bmjopen-2020-039049.R1
Article Type:	Original research
Date Submitted by the Author:	13-Jul-2020
Complete List of Authors:	Phull, Manraj ; West Hertfordshire Hospitals NHS Trust<br>Grimes, Caris; King's College London Faculty of Life Sciences and Medicine; Medway NHS Foundation Trust<br>Kamara, Thaim; University of Sierra Leone College of Medicine and Allied Health Sciences, Surgery<br>Wurie, Haja ; University of Sierra Leone College of Medicine and Allied Health Sciences<br>Leather, Andy; King's College London Faculty of Life Sciences and Medicine, King’s Centre for Global Health<br>Davies, Justine ; King's College London Faculty of Life Sciences and Medicine, Centre for Global Health; University of Birmingham Institute of Applied Health Research
**Primary Subject Heading**:	Health economics
Secondary Subject Heading:	Global health, Health policy, Surgery
Keywords:	SURGERY, HEALTH ECONOMICS, Health policy < HEALTH SERVICES ADMINISTRATION & MANAGEMENT

SCHOLARONE™
Manuscripts

I, the Submitting Author has the right to grant and does grant on behalf of all authors of the Work (as defined in the below author licence), an exclusive licence and/or a non-exclusive licence for contributions from authors who are: i) UK Crown employees; ii) where BMJ has agreed a CC-BY licence shall apply, and/or iii) in accordance with the terms applicable for US Federal Government officers or employees acting as part of their official duties; on a worldwide, perpetual, irrevocable, royalty-free basis to BMJ Publishing Group Ltd ("BMJ") its licensees and where the relevant Journal is co-owned by BMJ to the co-owners of the Journal, to publish the Work in this journal and any other BMJ products and to exploit all rights, as set out in our licence.

The Submitting Author accepts and understands that any supply made under these terms is made by BMJ to the Submitting Author unless you are acting as an employee on behalf of your employer or a postgraduate student of an affiliated institution which is paying any applicable article publishing charge ("APC") for Open Access articles. Where the Submitting Author wishes to make the Work available on an Open Access basis (and intends to pay the relevant APC), the terms of reuse of such Open Access shall be governed by a Creative Commons licence – details of these licences and which Creative Commons licence will apply to this Work are set out in our licence referred to above.

Other than as permitted in any relevant BMJ Author's Self Archiving Policies, I confirm this Work has not been accepted for publication elsewhere, is not being considered for publication elsewhere and does not duplicate material already published. I confirm all authors consent to publication of this Work and authorise the granting of this licence.

For peer review only

What is the financial burden to patients of accessing surgical care in Sierra Leone? A cross-sectional survey of catastrophic and impoverishing expenditure.

Manraj Phull (1), Caris E Grimes (2, 3), Thaim B Kamara (4), Haja Wurie (5), Andrew JM Leather (2) * and Justine Davies (2, 6, 7) *

*Co-senior authors

Authors

Manraj Phull BSc MBBS MRCS MSc
1) West Hertfordshire Hospitals NHS Trust. Watford General Hospital, Watford, Hertfordshire, UK

Caris E Grimes BSc MBBS MEd FRCS MD
2) King's Centre for Global Health and Health Partnerships, School of Population Health and Environmental Sciences, King's College London, London, SE5 9RJ, UK
3) Medway NHS Foundation Trust, Gillingham, Kent, UK

Thaim B Kamara MBChB, FWACS
4) College of Medicine and Allied Health Sciences, Freetown, Sierra Leone

Haja Wurie
5) College of Medicine and Allied Health Sciences, Freetown, Sierra Leone

Andrew JM Leather MBBS, FRCS, MS
2) King's Centre for Global Health and Health Partnerships, School of Population Health and Environmental Sciences, King's College London, London, SE5 9RJ, UK

Justine Davies (Corresponding Author) MBChB, MD (res), MRCP
2) King's Centre for Global Health and Health Partnerships, School of Population Health and Environmental Sciences, King's College London, London, SE5 9RJ, UK
6) Institute for Applied Health Research, University of Birmingham, Edgbaston, Birmingham B15 2TT UK
7) Medical Research Council/Wits University Rural Public Health and Health Transitions Research Unit, Faculty of Health Sciences, School of Public Health, University of the Witwatersrand, Johannesburg, South Africa

Correspondence to Justine Davies: J.Davies.6@bham.ac.uk

Author Contribution: JD, AL, TBK and HW conceptualised the study. MP, JD, and AL developed the protocol and survey tools; MP, JD, and CG analysed the data; all authors contributed to the interpretation of the results and write up of the manuscript; All authors approved the manuscript for publication.

Competing interests: None declared.

Funding: This research was partly funded by the National Institute of Health Research (NIHR) Global Health Research Unit on Health System Strengthening in Sub-Saharan Africa, King's College London (GHRU 16/136/54) using UK aid from the UK Government to support global health research. The

For peer review only - http://bmjopen.bmj.com/site/about/guidelines.xhtml

views expressed in this publication are those of the author(s) and not necessarily those of the NIHR or the Department of Health and Social Care.

Data Sharing: Further data is available on reasonable request from the corresponding author.

Patient and Public Involvement / Cohort Description: The Lancet Commission on Global Surgery has shown that out of pocket expenditure limits patients ability to access surgical care when needed. Accessing care for a surgically treatable disease to reduce mortality or morbidity is a priority for patients. The methodology employed was standard for assessing out of pocket costs, wealth, and healthcare expenditure. Patients were not involved in designing these methods, however, they were involved in testing and refining them to ensure appropriateness to a local setting. No patients were involved in the recruitment to and conduct of the study.

As part of the ethics board approval, we did not collect contact details of the patients involved in this study and hence cannot disseminate the results to them. However, the results are being shared widely amongst policy workers, community leaders, and clinicians in Sierra Leone. The patient advocacy movement in Sierra Leone, like in many low-income countries, is nascent, hence there are no patient groups with which to share results. We hope that our work will galvanise greater advocacy and enable sharing more widely.

We did not collect names of the six patients who helped to refine the data collection tool or ask their permission to be named. However, we have added a statement to the acknowledgements to generically acknowledge their contribution.

Acknowledgements: We thank the healthcare workers and patients who were involved in refining the data collection tool to ensure its applicability to a local setting.

For peer review only

Abstract

Objectives: To measure the financial burden associated with accessing surgical care in Sierra Leone.

Design: A cross-sectional survey conducted with patients at the time of discharge from tertiary level care. This captured demographics, yearly household expenditure, direct medical, direct non-medical, and indirect costs for surgical care, and summary household assets. Missing data were imputed.

Setting: The main tertiary level hospital in Freetown, Sierra Leone.

Participants: 335 surgical patients under the care of the hospital surgical team receiving operative or non-operative surgical care on the surgical wards.

Outcome measures: Rates of catastrophic expenditure (CE) (a cost > 10% of annual expenditure), impoverishment (being pushed into, or further into, poverty as a result of surgical care costs), amount of OOP costs, and means used to meet these costs were derived.

Results: Of 335 patients interviewed, 39.4% were female and 80.3% were urban dwellers. Median yearly household expenditure was US$3569. Mean out-of-pocket costs were US$243, of which a mean of US$24 (10%) was spent pre-hospital. Of costs incurred during the hospital admission, direct medical costs were US$138 (63%) and US$34 (16%) were direct non-medical costs. US$46 (21%) were indirect costs. Catastrophic expenditure affected 18% of those interviewed. 45% of patients were already below the national poverty line prior to admission, and 11% of those who were not were pushed below the poverty line following payment for surgical care. 83.7% of patients used household savings to meet OOP costs. Only 2% (6 patients) had health insurance.

Conclusion: Obtaining surgical care has substantial economic impacts on households which pushes them into poverty or further into poverty. The much-needed scaling up of surgical care needs to be accompanied by financial risk protection.

Article Summary

Strengths and Limitations

- Use of exit interviews to provide in depth data on costs of accessing surgical care.
- Thorough and detailed capture of household expenditure.
- Provides reliable estimates of OOP, catastrophic, and impoverishing expenditure as well as sources of financing.
- Data captured in one hospital only, although that is the major surgical care centre for the country.
- Only examines those who accessed care and doesn't allow exploration of costs as a limitation to accessing care.

For peer review only

Introduction

An estimated 33 million individuals globally face financial catastrophe through payment for surgery and anaesthetic care each year. Furthermore 3·7 billion people have been estimated to be at risk of catastrophic expenditure (CE – defined as a total OOP health payment that exceeds a set threshold of the household's annual income or expenditure) due to a lack of financial risk protection (FRP).[1,2] Surgical conditions make up 30% of the global burden of disease and globally an additional 143 million surgical procedures are required annually to meet the current unmet surgical need.[1,3] To ensure universal health coverage, it is therefore essential that FRP is prioritised alongside the scaling up of surgical care. The Lancet Commission on Global Surgery (LCoGS) stated a target of 100% financial protection by 2030 for people accessing surgical care, and FRP indicators for surgery are now included within the World Development Indicators (WDI).[4] Despite this, there is little information on financial implications of accessing surgery in the literature beyond modelled studies,[1,2,5] many of which have been based on few real-world data-points.

Worldwide modelled data on CE and impoverishing expenditure (IE – defined as being pushed into or further into poverty) related to surgical care reveals that those most affected are individuals in low- and middle-income countries (LMICs).[1,2,6] Modelling studies from Sierra Leone, classed as "least developed" by the UN, and with a population of 7 million reflects these findings; between 84.7% and 49.9% of the population in Sierra Leone is estimated to be at risk of CE if they require surgery. Estimated average out-of-pocket (OOP) costs for major surgery in the country were US$117.60, which put 73.3% to 59.2% of the population at risk of impoverishment.[5,7] However, there are no empirical data to validate these estimates. The estimated unmet surgical burden of disease in Sierra Leone is huge, at 92%, as a result of the historical neglect of surgical care both nationally and globally.[8-10] To enable effective planning of surgical services in future, an accurate understanding of the financial implications of accessing surgical services is required.

In Sierra Leone, as in many LMICs, payments for healthcare are upfront, complex, and not immediately apparent from hospital listed service charges. In addition, hospital listed charges – where they exist – may not reflect the total facility-incurred costs that patients pay during their hospitalisation. These include direct medical costs which are charges for the payment of medical care and direct non-medical costs which include items such as transport to the hospital and food. In addition, substantial costs of care may be incurred prior to the hospitalisation episode. For example, there may be direct medical costs at other healthcare facilities visited prior to the definitive admission. Finally, there are indirect costs (e.g. loss of wages whilst receiving care) that patients, and in some cases their caregivers, experience in their illness, which also impact upon ability to access care. Two ways of capturing these costs is the measurement of IE or CE. The two most widely used thresholds for CE are an expense of > 10% of total annual expenditure or > 40% of non-subsistence expenditure (i.e. household expenditure net of subsistence costs, as a means of capturing the ability to pay).[11-14]

This study aimed to measure the financial burden associated with receiving surgical care in Sierra Leone by using an exit survey to determine a) direct medical, direct non-medical, and indirect OOP costs to pay for a surgical care episode b) the rate of impoverishment and catastrophic expenditure, c) the wealth characteristics of the population accessing surgical care relative to that of the general Sierra Leonean population, d) the factors associated with higher costs of hospital care, e) the in-hospital payment mechanism (i.e. where and to whom the OOP payments are being made), and f) how costs of accessing surgical care are met, and the factors associated with meeting costs of care.

Methods

For peer review only - http://bmjopen.bmj.com/site/about/guidelines.xhtml

Setting

This study was done in the main tertiary referral centre in Sierra Leone, located in the central part of greater Freetown, and where the majority of surgical care in the countries' non charitable sector is done. It is a 400-bed hospital with 150 beds dedicated to surgical care. Surgical care is delivered in 5 of the 10 wards, an accident and emergency department with a trauma ward for short stay (< 24hrs) emergency surgical patients, a surgical outpatient unit, an intensive care unit and five operating theatres. The average surgical volume is 80 -100 operations per month.[15]. The surgical department is run by 8 surgical and 2 anaesthetic consultants covering six specialities: general surgery, surgical oncology, urology, paediatrics, trauma and orthopaedics, and ear, nose and throat (ENT) surgery. Obstetric and gynaecological surgical care is delivered at a nearby tertiary referral hospital dedicated to women's health, where all pregnant and lactating women receive free healthcare under the government's free health care initiative and therefore not included in this study.

Participants

Participants were all surgical patients who consented to take part, receiving operative or non-operative surgical care under the care of the hospital surgical team and located on one of the surgical wards. Patients under the care of non-surgical teams; patients under the age of 16 who were without a parent, guardian, or head of the household; and participants unable to consent and/or unwilling to take part in the study were excluded. Participants were recruited consecutively to the study on admission for surgical care from June to August 2018.

Data collection

A structured questionnaire was administered to patients and/or their relatives at the time of formal discharge from surgical care while patients were on the ward. Where patients self-discharged or left against medical advice, where possible they were interviewed when leaving the hospital. Interviews were conducted in a private space and all participants were encouraged to bring a relative, head of the household, or the main breadwinner to allow for expenditure and OOP costs to be captured accurately.

The questionnaire was designed based on tools used in similar studies done in LMIC settings.[16-19] It was co-designed with in-country experts, healthcare professionals, and researchers to ensure that the questions were suitable for the Sierra Leone context. The questionnaire was pilot-tested for ease of comprehension, clarity of definitions, appropriateness of questions, and manageability of the length of the interview in six patients (who were excluded from the analysis). Minor modifications were made to the wording of the questions based on this, but the meaning of the questions was not changed. The questionnaire was designed and written in English and administered by trained Sierra Leonean research assistants (RAs) in either English or a chosen local dialect (most commonly Krio). Data was captured on paper and later transferred to electronic format.

Definition and construction of variables

Data was collected on the particpants' age, gender and address (later used to calculate if they were resident in an urban or rural area). The occupation of the main breadwinner was recorded using free text followed by a question on whether this was salaried (i.e. employed) or non-salaried (i.e. self-employed or working in the informal sector). Education was captured as the highest level of education of the main breadwinner. Information on household expenditure was captured by asking 7 questions on regular items purchased in a typical week (food and drink etc.), 11 questions on larger expenditure items typically purchased monthly (toiletries, clothing, etc.) and a further 12 questions on typical yearly spend on big household items such as furniture and livestock (see Appendix 6). Total food expenditure ($foodexp_h$) was summed as a separate variable for the purposes of calculating CE (where food expenditure was used to define subsistence costs). Number of people living in the household (*HHsize)* was also captured, as was the number of days of sickness before presentation, whether care

For peer review only - http://bmjopen.bmj.com/site/about/guidelines.xhtml

had been sought elsewhere prior to presentation at Connaught Hospital, and the mode of transport used.

Data was also collected on the following: whether the patient was an emergency or elective case; whether or not the participant was eligible for free healthcare (for patients under the age of 5 years old, pregnant or lactating mothers, Ebola survivors, destitute and disabled patients); and the primary diagnosis, recorded from review of the patient's admission notes, ward and theatre ledgers (later summarised into 10 categories of surgical conditions: trauma, hernia, abdominal conditions, peripheral vascular disease or diabetic foot disease, urological conditions, breast mass / cancer, burns, ENT / dental disease, thyroid, congenital abnormality, or paediatrics. Treatment was categorised as operative or non-operative following review of the patient's admission notes. Length of hospital stay was also calculated.

Direct medical OOP costs were captured across the entire illness episode including in-hospital costs (from the point of admission to discharge from the tertiary care hospital) and pre-hospital costs (for other medical costs related to the admission episode which occurred prior to the tertiary care admission). In-hospital direct medical costs were the sum of administrative costs (including registration, admission, triage, bed and discharge fees), medications, medical supplies, investigations, blood transfusion, operation cost, and informal payments (defined as any payment that was not part of hospital policy, such as doctors' fees, tips, payments made to porters and to nursing staff for nursing care). If costs were 'formal', we asked whether these costs were paid directly to the hospital bank / cashiers directly or via hospital staff, or to an external facility (such as external pharmacy or laboratory). For pre-hospital care, non-medical direct costs were calculated from transport costs. For the hospital episode, non-medical direct costs were captured as: cost of transport to the hospital or to and from the hospital to get food, medical supplies and investigations from external facilities, and the cost of food and accommodation during the hospital stay. Finally, indirect costs were captured by estimating lost wages during the illness episode.

All costs are presented in Le and $US at the conversion rate of 15th July 2019 (1 Sierra Leonean Leone = 0.00011567 USD).

Total household expenditure ($totalexp_h$) was calculated over the course of 12 months by summing all the variables collected on all regular household items purchased as described above.

Total OOP payments for surgical care (OOP_t): = total direct medical costs + total direct non-medical costs + total indirect costs

Catastrophic expenditure (CE) is most widely defined as either an expense of more than 40% of non-subsistence expenditure (i.e. household expenditure net of subsistence [here, food ($foodexp_h$)] costs) or an expense more than 10% of total annual expenditure. We used both of these definitions in our calculations.

CE was therefore present if: $\frac{OOP_t}{totalexp_h - foodexp_h} > 0.4$

Or if: $\frac{OOP_t}{totalexp_h} > 0.1$

Impoverishing Expenditure (IE) is defined as being pushed into or further into poverty. The Sierra Leone national poverty line (spending < $1.25/person /day) threshold was used for the main analysis. In addition, two further thresholds for poverty were used based on World Bank definitions: "poverty"

For peer review only - http://bmjopen.bmj.com/site/about/guidelines.xhtml

- spending < \$3.10/person/day and "extreme poverty" - spending <\$1.90/person/day.[4] Presence of poverty before (baseline) and after OOP spending on surgical care were then calculated.

Baseline poverty (BLP_h) at each threshold was determined to be present if total household expenditure ($totalexp_h$) per individual inhabiting each household divided by the number of days in the year was below the poverty threshold chosen. i.e.: $\frac{\left(\frac{totalexp_h}{HHsize}\right)}{365} \leq poverty\ line$

Impoverishment as a result of surgical care was defined as present if the total household expenditure net the total OOP costs for surgical care ($totalexp_{netsurg} = totalexp_h - OOP_t$) per head of household, per day was less than the chosen poverty threshold

i.e.: IE present if $\frac{\left(\frac{totalexp_{netsurg}}{HHsize}\right)}{365} \leq poverty\ line$

Both CE and IE are presented as the number and percentage of participants who experienced CE and or IE.

Summary household asset data was collected using a yes or no response to the ownership of the following assets: Electricity / Light, Mobile phone, Radio, Television, Computer, Refrigerator, Generator, Bicycle, Motorcycle and Car or truck.

Sample size and power calculation

Based on a similar study done in Uganda which estimated CE to be 31%[16] in a free healthcare setting, modelled and World Bank data for Sierra Leone which estimates CE at 84.7% and 49.9% respectively, and from discussion with academics with in-country knowledge, we estimated that CE would be around 60% of patients admitted for surgical care. The sample size required to capture this with a CI of 55-65%, allowing for 10% loss to follow up was 442 patients.

Statistical analysis

Statistical analysis was done using SPSS Version 25 for windows.

Characteristics of the population seeking care are described. Normally distributed data are presented as mean and standard deviation (SD), otherwise median, IQR and range are used. Multiple Imputation Chained Equations were used to compute missing data-points using predictive mean matching for variables with less than 20% missingness and where missingness was identified as not at random. Where imputed variables were used, the pooled mean is shown as standard SPSS output. A complete case analysis was done for variables describing how costs of accessing care were met and the consequences of accessing care.

Wealth characteristics (household asset ownership) of the population accessing surgical care were compared with those in the general population (2015 Census data[20]) using the Chi squared test.

Associations between direct medical in-hospital OOP costs of care and age, sex, type of admission (emergency or elective), operative or non-operative care, type of operative procedure, or length of stay were tested using a generalised linear model using a Tweedie function with a power of 1.9.

Ethical approval

Ethical approval was granted by the Sierra Leone Ethics and Scientific Review Committee and from the King's College London Research Ethics Committee (ref. LRU-17/18-6455)

For peer review only - http://bmjopen.bmj.com/site/about/guidelines.xhtml

All patients gave written consent to participate where possible and witnessed thumbprints and verbal consent where patients were illiterate. Patients were given information about the study at admission and consented between 4-24 hours later after due time was given to consider the study information. Consent was re-confirmed just prior to doing the exit interview.

Results

Of the initial 416 recruited participants, a total of 335 were interviewed (Figure 1). Participant characteristics are presented in table 1. In summary, the mean age of the interviewed patients was 28 (SD 20). 39.4% were female and 80.3% lived in an urban area. 29% were formally employed with a further 66.9% being employed but without a regular salary – either self-employed or employed within the informal sector. The level of education of the main breadwinner was secondary school in 37.9%, college / university in 28% and no formal education in 23.6%. The median household size was 6 (IQR: 4, range: 4-8) with a mean total yearly household expenditure of US$3569 (see appendix table 1 for imputed and non-imputed data and appendix table 2 for a comparison with expenditure assessed in the Economic and Financial survey in 2014[21]). 67.2% of participants had sought care for their illness elsewhere prior to presentation at the tertiary referral hospital. 71.9% arrived by public transport and the majority were classed as emergency admissions (72.2%). The most common reasons for presentation were trauma, hernia, or other abdominal conditions. 67.5% underwent operative intervention with the remainder being managed by non-operative measures. Median length of stay was 8 days (IQR: 18, range: 3-21).

Table 1: Participant characteristics

**Demographics of participants**	
Total number of patients interviewed	335
Mean age in years (SD)	28 (20)
Female number (%)	132 (39.4%)
Urban Residents (%)	269 (80.3%)
**Type of job (number (%)):**<br>Self Employed / Informal Sector<br>Employed<br>Unemployed / Retired<br>Missing /Don't know	 224 (66.9%)<br>97 (29%)<br>12 (3.6%)<br>2 (0.6%)
**Level of education of main breadwinner (number (%)):**<br>No formal education<br>Primary school<br>Secondary school<br>College / University<br>Other / Missing / Don't know	 79 (23.6%)<br>25 (7.5%)<br>127 (37.9%)<br>94 (28%)<br>10 (3.0%)
Median household size (IQR, (range))	6 (4, (4-8))
Total yearly household expenditure (US$)	$ 3,569
Number below national poverty line prior to illness	151 (45%)
**Surgical Care Episode Descriptors**	
Median days of sickness before presentation (IQR, (range))	2 (14, (0-14))
Number that sought care for illness elsewhere prior to presentation at Connaught	225 (67.2%)
**Mode of transport used to travel to hospital (number (%)):**<br>Public transport<br>Ambulance	241 (71.9%)<br>67 (20%)<br>23 (6.9%)

For peer review only - http://bmjopen.bmj.com/site/about/guidelines.xhtml

Private transport<br>Walked<br>Don't know / Missing	3 (0.9%)<br>1 (0.3%)
Emergency admission (%)	242 (72.2%)
Eligible for free health care (%)*	70 (20.9%)
**Primary diagnosis by surgical condition (number (%))**	
Trauma	114 (34%)
Hernia	58 (17.3%)
Abdominal conditions	56 (16.7%)
Peripheral vascular disease or diabetic foot disease	27 (8.1%)
Urological conditions	23 (6.9%)
Breast mass / cancer	16 (4.8%)
Burns	15 (4.5%)
ENT / dental disease	13 (3.9%)
Goitre	7 (2.1)
Congenital abnormality (paediatrics)	3 (0.9%)
Missing / don't know	3 (0.9%)
**Treatment (number (%)):**	
Operative	226 (67.5%)
Non-operative	109 (32.5%)
Median length of hospital stay (LOS) in days (IQR, (range))	8 (18, (3-21))

* Eligible for free health care indicates those that fall under the government Free Health Care Initiative (FHCI); a health financing policy introduced in 2010 aimed to significantly improve maternal and child health through the provision of free healthcare services for all children under 5, pregnant and lactating women. This was later extended to include Ebola survivors.

The total mean cost for the surgical care episode was US$243 of which US$24 (10%) accounted for pre-hospital direct costs (medical costs were US$21 and non-medical were US$3). Of the in-hospital direct costs (mean US$172), a mean of US$138 (63%) was due to direct medical costs and US$34 (16%) for direct non-medical costs. Indirect costs, such as lost wages, totalled US$46. (Table 2 and appendix table 3).

Table 2: Out-of-pocket costs.

Costs	Imputed mean cost ($US (% of subtotal))
**Prehospital costs**	
**Direct pre-hospital medical OOP costs (total)**	**21 (88% of 24)**
- Consultation	2 (10% of 21)
- Medications	12 (57% of 21)
- Medical supplies	2 (10% of 21)
- Investigations	4 (19% of 21)
- Other miscellaneous	2 (10% of 21)
**Direct (pre-hospital) non-medical OOP costs (total)**	**3 (13% of 24)**
- Transport	3 (100% of 3)
Total pre-hospital costs	***24 (10% of 243)***
**In hospital costs**	
**Direct medical OOP costs (total)**	**138 (63% of 219)**
- Administrative	20 (14% of 138)

For peer review only - http://bmjopen.bmj.com/site/about/guidelines.xhtml

- Medications	26 (19% of 138)
- Medical supplies	14 (10% of 138)
- Investigations	15 (11% of 138)
- Blood transfusion	9 (7% of 138)
- Total operation costs	49 (36% of 138)
- Unofficial costs	6 (4% of 138)
- Other / miscellaneous	1 (1% of 138)
**Direct non-medical costs (total)**	**34 (16% of 219)**
- Transport to hospital	7 (21% of 34)
- Food	20 (59% of 34)
- Accommodation	0 (0% of 34)
- Other*	7 (21% of 34)
**Indirect costs**	
- Lost wages	46 (100% of 46)
**TOTAL OOP COSTS**	**243**

*other relates to travel and other associated costs incurred as a result for needed investigations from and or medication / supplies from an external facility. SPSS calculates only the mean using imputed variables, hence no standard deviation is displayed.

For peer review only - http://bmjopen.bmj.com/site/about/guidelines.xhtml

Of the in-hospital direct medical costs, 48% were given to hospital staff (it was not clear whether the hospital staff later transferred these funds to the hospital bank or not), 33% were made directly to the hospital bank / cashiers and 17% to an external facility such as external pharmacy or diagnostic centre (Appendix table 4).

A variety of means were used to meet costs and participants were allowed to mention more than one means of covering costs (Table 3). Most (83.7% of patients) used their savings to meet some or all of the costs, with family contributions, borrowing money and charitable donations forming the 2nd, 3rd and 4th most frequently used means of meeting OOP payments, respectively. Only 2% (6 patients) had some form of health insurance. Wider implications included loss of wages in 36.9% and loss of job in 6.0%.

Table 3: How costs are met and the wider implications of seeking and undergoing surgical care (n is the number of cases with data on each variable)

How costs were met (total number responding to question)	Number (%) that used this as a means of meet OOP costs
Used **Savings** (n=326)	**273 (83.7%)**
Arranged **family contributions** (n=331)	**128 (38.7%)**
**Borrowed** money (n=331)	**102 (30.8%)**
Received **charity** money (n = 331)	**83 (25.3%)**
**Sold possessions** (n=329)	**17 (5.2%)**
**Other** (n=331)	**14 (4.2%)**
**Pawned** possessions (n=332)	**8 (2.4%)**
Have **Health insurance** (n=335)	**6 (1.8%)**
**Wider implications**	**Number (%) that experienced the wider implications of meeting OOP costs**
**Loss of wages** (n = 328)	**121 (36.9%)**
**Lost their job** / changed their role at work / home (n = 331)	**20 (6.0%)**
**Disruption to education** (n = 333)	**12 (3.6%)**

Catastrophic expenditure, when defined as OOP costs of more than 40% of non-subsistence expenditure affected 10% of those interviewed, rising to 18% when defined as out-of-pocket costs more than 10% of all household expenditure.

Prior to the surgical care episode, 45% of people interviewed were below the national poverty line, 90% were below the World Bank Poverty Level, and 70% below the World Bank Extreme Poverty level. Following payment for surgical care, 50% were pushed below or further below the national poverty line. Corresponding figures were 91% and 73%, for the World Bank thresholds of poverty and extreme poverty, respectively.

Analysis of the possession of household assets demonstrated that those interviewed were more likely to have electricity, a mobile phone, radio, television, refrigerator, bicycle, motorcycle or car than those of the general population in Sierra Leone (2015 Census data, all p=<0.001) or of the urban population in the Western Area (2015 Census data, all p=<0.05) (Table 4).

Regression analysis demonstrated that the factors associated with greater costs were older age, longer length of hospital stay and undergoing a general surgical or urological procedure (Appendix table 5).

For peer review only - http://bmjopen.bmj.com/site/about/guidelines.xhtml

Discussion

In this study, we found that accessing and receiving tertiary level surgical care in Sierra Leone requires large up-front OOP payments which have a substantial impact on individual and households' economic situations. These equate to a catastrophic expense in nearly a fifth of households and are impoverishing half of the households that receive care. We found poverty, as assessed by household expenditure, was high indicating a limited financial buffer to accommodate costs of care. This is despite most people who access surgical care owning a higher level of assets that the general population.

The majority of the OOP payments were incurred in-hospital and as a result of direct medical costs. Payment for the operation itself and medications, medical supplies, and investigations (including laboratory tests) were the biggest contribution to these costs. A small percentage of costs were categorised as unofficial, such as for "nursing care" and "tips", although these were given by a majority of people who received care. In addition, in enquiring about the payment routes for formal costs, we identified that almost half of these were being paid through unofficial payment channels and made directly to staff. We do not know whether these payments were later transferred by staff to the hospital bank, however, that these informal routes are common and indicate poor financial governance which urgently needs to be addressed.

Assessment of the wider implications of seeking surgical care in Sierra Leone highlighted that the majority of payments were met using savings, followed by raising money from family contributions or borrowing money which may leave households in debt. In addition, a large number of participants lost wages during the sickness episode and a proportion lost their jobs. In a country where informal work predominates and earnings can be unpredictable, this may impact on household financial security and influence future health seeking behaviour, both of the individuals affected and their immediate family and communities.

The majority of patients accessing surgical care were young males; whether this male predominance is a true reflection of surgical disease burden, beyond obstetrics and gynaecological care, in Sierra Leone or reveals a hidden gender bias in care seeking behaviour is beyond the remit of this study. Nevertheless, males who sought care in our study are traditionally the main breadwinners and the most economically active population group in Sierra Leone. This loss of wages and livelihood could have implications on the wider socio-economic determinants of health and the well-being of the household. The additional burden to the patients and their households as a result of the indirect costs supports the macroeconomic argument for investing in surgical care put forward by Grimes et al, who demonstrated the opportunity to avert 36,487 DALYs by investing in surgical care at hospital level in Sierra Leone. [22,23]

Some specialties, such as general surgery and urology incurred much higher overall costs for the surgical episode and this may be because operative intervention (with blood transfusion and a longer length of stay) is usually required. This contrasts for example, to trauma care that was often managed non-operatively. Such non-operative treatment for trauma may be partly as a result of local surgical practice, often driven by lack of resources such as the unavailability of internal fixation wires and orthopaedic implants, and partly because some common orthopaedic problems are managed non-operatively. In addition, we found that age and length of hospital stay were associated with significantly higher costs. This may be due to the fact that those under the age of 5 years were eligible for free health care in Sierra Leone and that a longer stay in hospital was associated with higher direct non-medical and indirect costs such as payment for food and lost wages.

There are a limited number of studies to draw a direct comparison with as only a few used a similar methodology (direct interview) as opposed to modelled data or the use of caesarean section costs as

For peer review only - http://bmjopen.bmj.com/site/about/guidelines.xhtml

a proxy measure to extrapolate costs, CE and impoverishment.[2,16,24-29] There are even fewer studies that report on the financial implications of all or most types of surgical care. The majority report on single surgical subspecialties such as obstetric care, paediatric surgery or trauma care. Nevertheless, there have been three recent studies from Uganda reporting CE rates of 31% and IE of 47% (assessing all surgical categories), CE of 55% (in adult surgery only) and 45% (in paediatric surgery).[16,30,31] A study in Malawi interviewing patients undergoing hernia operations reported CE rates as high as 90% using a threshold of 10% of yearly income.[27] Various studies looking at injury and trauma care costs in Vietnam, India and Nigeria have reported CE rates of 60%, 30% and 86% respectively[24] and a study in Morocco looking at obstetric surgical care alone estimated CE rates of 88%[32] while an emergency obstetric care study in Indonesia estimated CE at 68%.[33] This highlights the inter-country variability and although that makes it difficult to draw comparative conclusions, our study results relate closest to the Ugandan study that assessed all surgical categories using a similar methodology and thresholds. This highlights the need for a standardised way of assessing and measuring the financial implications of surgical care, to allow accurate collection and reporting of these global surgery metrics on financial risk protection.

In keeping with other studies, we noted lower rates of CE and IE in comparison to the modelled and extrapolated estimates for Sierra Leone. This is probably because the modelled studies are based on the whole population that may require surgery and not on those that have successfully accessed surgical care. The lower rates of IE and CE seen may therefore be explained by a lack of access by the poorest. This is supported by data from Sierra Leone that estimates that up to 25% of deaths in 2011 could have been averted through access to safe, timely and affordable surgical care and that Sierra Leone has an unmet surgical burden of disease of 92% [10], with approximately 70% of Sierra Leoneans stating that the financial burden of OOP payments for healthcare was the biggest barrier to accessing care.[34,35] In addition, we found that those accessing tertiary level surgical care came from predominantly urban areas of Sierra Leone and when compared to the wider Sierra Leone population, had significantly higher asset ownership. Using the latter as a proxy of wealth or socio-economic status, this could indicate that those able to access and receive surgical care represent the relatively wealthier households of Sierra Leone and therefore that the poorest and those at the highest risk of financial catastrophe are not accessing care when needed. This may also reflect other known barriers to seeking surgical care in LMICs that are often complex and multifactorial such as cultural beliefs, attitudes and fears towards surgical care and structural barriers such as geographical access, transport links and referral systems.[36]

Limitations

There are several limitations to this study. Firstly, it was dependant on recall and self-reported estimates of OOP costs and household expenditure. Although the questionnaire and methodology are a well-established way of obtaining this information in a low-resource setting where informal work predominates and payments are not often receipted, the data is still subject to respondent and recall bias. This may have contributed to missing data or the underestimation of OOP costs and possible overestimation of household expenditure which is also subject to social desirability bias.

Attempts made to minimise this included breaking down household expenditure questions to weekly, monthly and yearly costs, using a chronological approach to the OOP cost questions that helped map out the patients journey for them, encouraging participants to bring an appropriate family member to the interview, and by gaining in-country consensus and piloting the questionnaire prior to use.

Whilst attempts were made to ensure another family member or respondent was available during the interview to assist with accuracy, this was not always possible. In addition, given the reliance on savings, borrowed money and family contributions, payments were often made without the knowledge of the patient and were therefore difficult to reliably collect. As a result, the data was

For peer review only - http://bmjopen.bmj.com/site/about/guidelines.xhtml
For peer review only

handled using an established statistical approach that aimed to accurately account for the missing values. Further comparison between non-imputed and imputed data was performed which showed minimal disparity suggesting reliability of this method (see Appendix).

Secondly, given that patients were often interviewed on the wards and potentially within hearing range of nurses, data on informal payment methods and informal costs, may not have been fully reported. With this in mind we would have expected to see more missing data for the variable payments made directly to staff in comparison to those made to the banks, however we did not observe this. This indicates that participants were not deterred from sharing information on informal payments within the in-hospital study setting.

Thirdly, the study only measures costs incurred during the illness episode up until discharge and therefore does not capture the indirect costs if patients are off work or do not return back to work following discharge or the costs of ongoing outpatient or follow-up care, medications and medical supplies. We have therefore likely substantially underestimated the total costs of seeking surgical care.

In addition, Sierra Leone tertiary level obstetric care is provided at a different hospital and offered free of charge. Therefore, costs of accessing this were not included in this study. Further work needs to be done to see if those receiving free maternal healthcare incur any OOP costs and if informal payments such as tips paid to staff are as prevalent in the obstetric care hospital.

Finally, the desired sample size was not achieved as not all surgical patients admitted were interviewed. This was mostly due to many being discharged out of hours, at the weekend or after a short admission on the acute trauma ward, before the study team could consent or interview them. With regards to the later this may indicate minor pathology, a shorter stay and therefore lower OOP costs. Inclusion of these cases may have lowered the mean OOP costs, CE and IE rates but would poorly represent the financial barriers and wider implications of accessing surgical care for those that may have absconded or self-discharged due the cost of care. Nevertheless, although sample size was not obtained, the 95% confidence interval for a catastrophic expenditure rate of 18% was 14-22% which gives the study an overall power of 90%.

Conclusion

This is the first empirical study from Sierra Leone that quantifies the financial burden of accessing and receiving surgical care. It adds insight into the global and national Sierra Leone modelled estimates of the likelihood of catastrophic and impoverishing expenditure if surgery is required and joins the small but growing body of other empirical studies reporting on the OOP costs and wider financial implications of surgical care. In addition, it highlights the need to prioritise financial risk protection within healthcare and surgery if universal health coverage is to be achieved.

References

1. Meara JG, Leather AJ, Hagander L, et al. Global Surgery 2030: evidence and solutions for achieving health, welfare, and economic development. *Lancet* 2015; **386**(9993): 569-624.

For peer review only - http://bmjopen.bmj.com/site/about/guidelines.xhtml

For peer review only

2. Shrime MG, Dare AJ, Alkire BC, O'Neill K, Meara JG. Catastrophic expenditure to pay for surgery worldwide: a modelling study. *Lancet Glob Health* 2015; **3 Suppl 2**: S38-44.
3. Shrime MG, Bickler SW, Alkire BC, Mock C. Global burden of surgical disease: an estimation from the provider perspective. *Lancet Glob Health* 2015; **3 Suppl 2**: S8-9.
4. World Bank Development Indicators 2016. http://data.worldbank.org/data-catalog/world-development-indicators (accessed 10th August 2018.
5. Shrime MG, Dare A, Alkire BC, Meara JG. A global country-level comparison of the financial burden of surgery. *Br J Surg* 2016; **103**(11): 1453-61.
6. WHO. World Health Organisation: Health statistics and information systems. Global Reference List of 100 Core Health Indicators. . 2015. http://www.who.int/healthinfo/indicators/2015/en/
7. World Bank: Risk of catastrophic expenditure for surgical care. Sierra Leone https://data.worldbank.org/indicator/SH.SGR.CRSK.ZS?locations=SL.
8. Farmer PE, Kim JY. Surgery and global health: a view from beyond the OR. *World J Surg* 2008; **32**(4): 533-6.
9. Bolkan HA, Von Schreeb J, Samai MM, et al. Met and unmet needs for surgery in Sierra Leone: A comprehensive, retrospective, countrywide survey from all health care facilities performing operations in 2012. *Surgery* 2015; **157**(6): 992-1001.
10. Groen RS, Samai M, Stewart KA, et al. Untreated surgical conditions in Sierra Leone: a cluster randomised, cross-sectional, countrywide survey. *Lancet* 2012; **380**(9847): 1082-7.
11. Hsu J, Flores G, Evans D, Mills A, Hanson K. Measuring financial protection against catastrophic health expenditures: methodological challenges for global monitoring. *Int J Equity Health* 2018; **17**(1): 69.
12. Xu K, Evans DB, Carrin G, Aguilar-Rivera AM, Musgrove P, Evans T. Protecting households from catastrophic health spending. *Health Aff (Millwood)* 2007; **26**(4): 972-83.
13. Hoang HT, Pham TL, Vo TT, Nguyen PK, Doran CM, Hill PS. The costs of traumatic brain injury due to motorcycle accidents in Hanoi, Vietnam. *Cost Eff Resour Alloc* 2008; **6**: 17.
14. Nguyen H, Ivers R, Jan S, Martiniuk A, Pham C. Catastrophic household costs due to injury in Vietnam. *Injury* 2013; **44**(5): 684-90.
15. National Surgical, Obstetric and Anaesthesia Strategic Plan. Sierra Leone Ministry of Health and Sanitation.
16. Anderson GA, Ilcisin L, Kayima P, et al. Out-of-pocket payment for surgery in Uganda: The rate of impoverishing and catastrophic expenditure at a government hospital. *PLoS One* 2017; **12**(10): e0187293.
17. World Health Organisation. Health statistics and information systems. Data collection tools - World Health Survey. . http://www.who.int/healthinfo/survey/en/ (accessed 19th April 2018).
18. (SAGE). WHOHsaisDct-WSoGAaAH. http://www.who.int/healthinfo/sage/en/
19. Grimes CE, Billingsley ML, Dare AJ, et al. The demographics of patients affected by surgical disease in district hospitals in two sub-Saharan African countries: a retrospective descriptive analysis. *Springerplus* 2015; **4**: 750.
20. 2015 Population and House Census: Summary of Final Results. . https://www.statistics.sl/images/StatisticsSL/Documents/final-results_-2015_population_and_housing_census.pdf. Accessed 21st August 2019. .
21. Economic and Financial Survey 2014. . https://www.statistics.sl/images/StatisticsSL/Documents/Publications/2014/econmic_and_financial_survey_2014_2.pdf
(accessed 20th June 2019)
.
22. Grimes CE, Quaife M, Kamara TB, Lavy CBD, Leather AJM, Bolkan HA. Macroeconomic costs of the unmet burden of surgical disease in Sierra Leone: a retrospective economic analysis. *BMJ Open* 2018; **8**(3): e017824.
23. Grimes CE, Law R, Dare A, et al. Cost-Effectiveness of Two Government District Hospitals in Sub-Saharan Africa. *World J Surg* 2017; **41**(9): 2187-92.
24. Nguyen H, Ivers R, Jan S, Pham C. Cost of surgery and catastrophic expenditure in people admitted to hospital for injuries: estimates from a cohort study in Vietnam. *Lancet* 2015; **385 Suppl 2**: S50.
25. Prinja S, Bahuguna P, Gupta R, Sharma A, Rana SK, Kumar R. Coverage and Financial Risk Protection for Institutional Delivery: How Universal Is Provision of Maternal Health Care in India? *PLoS One* 2015; **10**(9): e0137315.
26. Honda A, Randaoharison PG, Matsui M. Affordability of emergency obstetric and neonatal care at public hospitals in Madagascar. *Reprod Health Matters* 2011; **19**(37): 10-20.

For peer review only - http://bmjopen.bmj.com/site/about/guidelines.xhtml

27. Bijlmakers L, Wientjes M, Mwapasa G, et al. Out-of-pocket payments and catastrophic household expenditure to access essential surgery in Malawi - A cross-sectional patient survey. *Ann Med Surg (Lond)* 2019; **43**: 85-90.
28. Banu T, Chowdhury TK, Aziz TT, et al. Cost Incurred by the Family for Surgery in Their Children: A Bangladesh Perspective. *World J Surg* 2018; **42**(12): 3841-8.
29. Urua U, Osungbade K, Obembe T, Adeniji F. A cost analysis of road traffic injuries in a tertiary hospital in south-west Nigeria. *Int J Inj Contr Saf Promot* 2017; **24**(4): 510-8.
30. MacKinnon N, St-Louis E, Yousef Y, Situma M, Poenaru D. Out-of-Pocket and Catastrophic Expenses Incurred by Seeking Pediatric and Adult Surgical Care at a Public, Tertiary Care Centre in Uganda. *World J Surg* 2018; **42**(11): 3520-7.
31. Yap A, Cheung M, Kakembo N, et al. From Procedure to Poverty: Out-of-Pocket and Catastrophic Expenditure for Pediatric Surgery in Uganda. *J Surg Res* 2018; **232**: 484-91.
32. Boukhalfa C, Abouchadi S, Cunden N, Witter S. The free delivery and caesarean policy in Morocco: how much do households still pay? *Trop Med Int Health* 2016; **21**(2): 245-52.
33. Quayyum Z, Nadjib M, Ensor T, Sucahya PK. Expenditure on obstetric care and the protective effect of insurance on the poor: lessons from two Indonesian districts. *Health Policy Plan* 2010; **25**(3): 237-47.
34. Ponsar F, Tayler-Smith K, Philips M, et al. No cash, no care: how user fees endanger health--lessons learnt regarding financial barriers to healthcare services in Burundi, Sierra Leone, Democratic Republic of Congo, Chad, Haiti and Mali. *Int Health* 2011; **3**(2): 91-100.
35. Xu K, Evans DB, Kawabata K, Zeramdini R, Klavus J, Murray CJ. Household catastrophic health expenditure: a multicountry analysis. *Lancet* 2003; **362**(9378): 111-7.
36. Grimes CE, Bowman KG, Dodgion CM, Lavy CB. Systematic review of barriers to surgical care in low-income and middle-income countries. *World J Surg* 2011; **35**(5): 941-50.

For peer review only

For peer review only

For peer review only - http://bmjopen.bmj.com/site/about/guidelines.xhtml

For peer review only

For peer review only - http://bmjopen.bmj.com/site/about/guidelines.xhtml

Figure 1

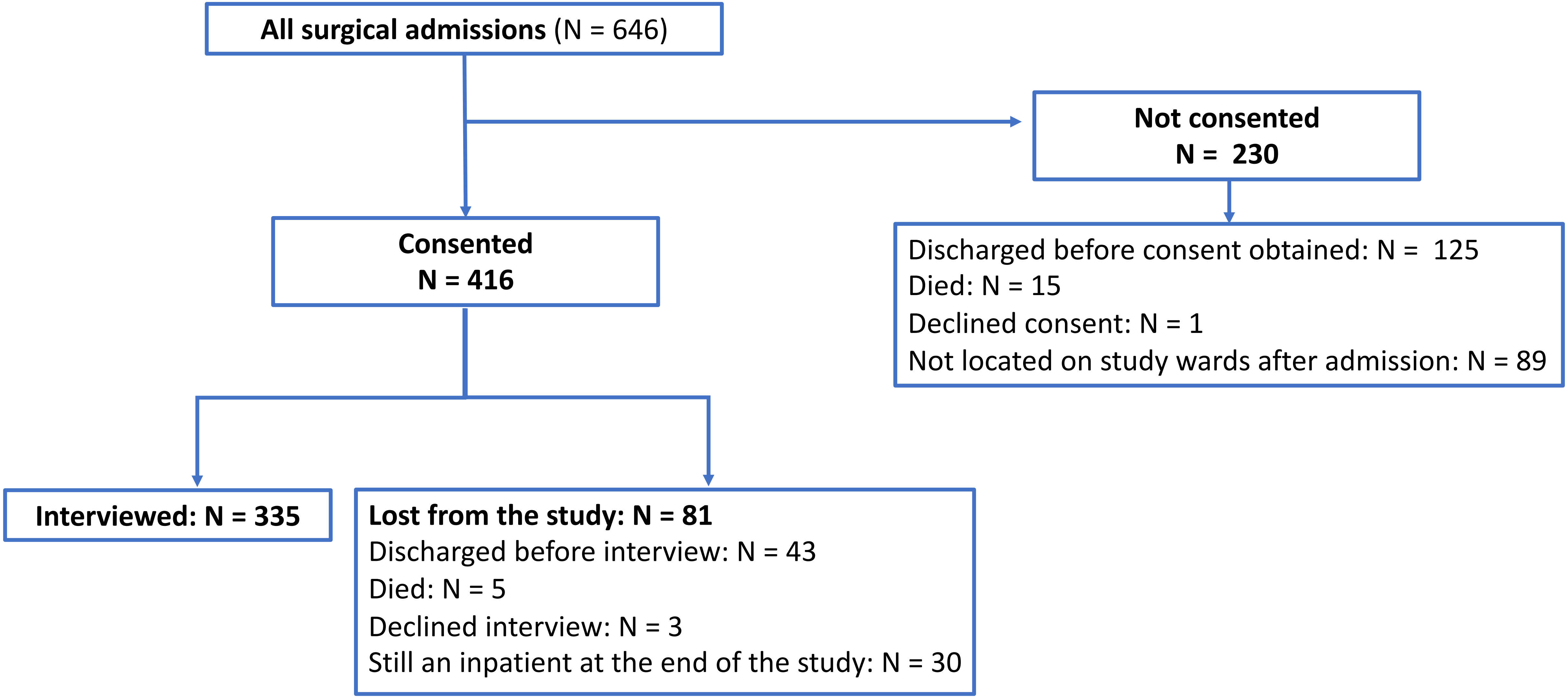

Figure 1: Study recruitment process diagram. Flow diagram of the total number of admissions (646) during the study period, highlighting those recruited, lost from the study and reasons for this.

Appendix

Additional information on recruitment and training of research assistants

RAs were recruited through a competitive process and trained to administer the questionnaire. Training for all RAs was standardised and formally ran over 2 days. This involved; a formal presentation introducing the study, a review of all study processes and associated documents, a role play interview between the RAs using the questionnaire, a walk through the hospital to ensure the RAs gained an insight in to the surgical patients' journey and points at which OOP payments may be made or cost incurred and a review of clinical notes, ward admission books and theatre log books to ensure that all demographic and diagnostic information was accurately captured.

Appendix table 1: Household expenditure showing imputed and non-imputed data sets.
Comparison of non-imputed and imputed data on household expenditure using Multiple Imputation Chained Equations to compute missing data-points using predictive mean matching.

Household expense	Non-imputed data mean (SD) (Le and $US)	Imputed data pooled mean (Le and $ US)
**Individual Consumption Expenditure by Households Including all variables collected**	**Le 39,665,597 (53,679,740) $ 4,425 (5,989)**	**Le 47,944,384 $5,349**
**Individual Consumption Expenditure by Households Excluding variables with > 20% missing data i.e. clothing, mobile phone credit and transport**	**Le 28,134,505 (31,539,987) $ 3,139 (3,519)**	**Le 31,988,507 $ 3,569**
Food and non-alcoholic beverages	Le 18,616,404 (22,364,391) $ 2,077 (2,495)	Le 20,867,118 $ 2,328
Alcoholic Beverages, Tobacco and Narcotics	Le 252,991 (1,047,612) $ 28 (117)	Le 314,095 $ 35
Rental	Le 884,123 (4,670,009) $ 99 (521)	Le 876,940 $ 98
Household maintenance	Le 108,092 (298,466) $ 12 (33)	Le 130,857 $ 15
Electricity, gas and other fuels	Le 720,748 (1,068,694) $ 80 (119)	Le 747,701 $ 83
Furnishings, household equipment and routine household maintenance	Le 632,577 (1,065,761) $ 71 (119)	Le 699,188 $ 78
Healthcare (traditional and western medicine)	Le 561,770 (1,490,207) $ 63 (166)	Le 740,205 $ 83
Recreation and cultural services	Le 821,684 (1,894,677) $ 92 (211)	Le 1,121,965 $ 125
Education	Le 1,338,183 (2,295,578) $ 149 (256)	Le 1,614,982 $ 180
Personal care / toiletries	Le 609,012 (629,656) $ 68 (70)	Le 627,269 $ 70
Health insurance	Le 8,333	Le 13,370

For peer review only - http://bmjopen.bmj.com/site/about/guidelines.xhtml

	(138,661)<br>$ 1 (15)	$ 1
Remittance	Le 974,408<br>(1,655,020)<br>$ 109 (185)	Le 1,015,363<br>$ 113
Donations	Le 185,233<br>(475,436)<br>$ 21 (53)	Le 203,246<br>$ 23
Livestock	Le 41,400<br>(213,804)<br>$ 5 (24)	Le 45,254<br>$5
Taxes	Le 59,500<br>(261,497)<br>$ 7 (29)	Le 73,723<br>$ 8

Appendix table 2: Household expenditure data. Variables on household expenditure shown here, for broad comparison, with the Economic and Financial survey Sierra Leone 2014 data[21]. Categories were harmonised where possible, however given differences in questions asked between surveys, an exact match of categories was not possible to achieve. Costs from the 2014 Economic and Financial Survey were not adjusted for inflation which needs to be considered when reviewing this data.

**Household consumption expenditure (in Leones (Le) and USD ($))**		
**Household Expense**	**Sierra Leone Economic and Financial Survey data 2014**	**Study data**
**Individual Consumption Expenditure by Households (Total Expenditure)**	**Le 15,414,816<br>$ 1,739**	**Le 31,988,507<br>$ 3,569**
**Food and non-alcoholic beverages**	**Le 6,838,365<br>$ 771**	**Le 20,867,118<br>$ 2,328**
Food	Le 6,644,019<br>$ 74	Le 17,925,090<br>$ 2,000
Non-alcoholic beverages	Le 194,346<br>$ 22	Le 2,942,028<br>$ 328
**Alcoholic Beverages, Tobacco and Narcotics**	**Le 450,612<br>$ 51**	**Le 314,095<br>$ 35**
**Housing, water, electricity, gas and other fuels**	**Le 1,058,449<br>$119**	**Le 875,672<br>$ 98**
**Rental**	**Le 253,948<br>$ 29**	**Le 876,940<br>$ 98**
**Maintenance and repair of the dwelling**	**Le 32,650<br>$ 4**	**Le 876,940<br>$ 98**
**Electricity, gas and other fuels**	**Le 595,832<br>$ 67**	**Le 747,701<br>$ 83**
**Furnishings, household equipment and routine household maintenance**	**Le 413,364<br>$ 47**	**Le 699,188<br>$ 78**
Furnishings	Le 88,058<br>$ 10	Le 196,217<br>$ 22
Household appliances	Le 41,821<br>$ 5	Le 314,772<br>$ 35
Tools and equipment for house and garden	Le 45,403<br>$ 5	Le 57,344<br>$ 6
Goods and services for routine household maintenance	Le 105,438<br>$ 12	Le 130,857<br>$ 15
Purchase of vehicles	Le 108,710<br>$ 12	Le 160,759<br>$ 18
Educations	Le 794,478<br>$ 90	Le 1,614,982<br>$ 180

For peer review only - http://bmjopen.bmj.com/site/about/guidelines.xhtml

Insurance (HI)	Le 38,890<br>$ 4	Le 13,370<br>$ 1

For peer review only - http://bmjopen.bmj.com/site/about/guidelines.xhtml

Appendix table 3: Imputed and non-imputed data for out-of-pocket costs for comparison.

Costs	Imputed Mean cost ($US (% of subtotal))	Non-imputed Mean cost ($US) (SD)
**Prehospital costs**		
**Direct pre-hospital medical OOP costs (total)**	**21 (88% of 24)**	**14 (65)**
- Consultation	2 (10% of 21)	1 (6)
- Medications	12 (57% of 21)	9 (46)
- Medical supplies	2 (10% of 21)	2 (8)
- Investigations	4 (19% of 21)	4 (25)
- Other miscellaneous	2 (10% of 21)	1 (10)
**Direct (pre-hospital) non-medical OOP costs (total)**	**3 (13% of 24)**	**3 (9)**
- Transport	3 (100% of 3)	3 (9)
Total pre-hospital costs	***24 (10% of 243)***	***25 (75)***
**In hospital costs**		
**Direct medical OOP costs (total)**	**138 (63% of 219)**	**109 (121)**
- Administrative	20 (14% of 138)	16 (24)
- Medications	26 (19% of 138)	25 (61)
- Medical supplies	14 (10% of 138)	11 (33)
- Investigations	15 (11% of 138)	14 (23)
- Blood transfusion	9 (7% of 138)	9 (22)
- Total operation costs	49 (36% of 138)	51 (75)
- Unofficial costs	6 (4% of 138)	5 (9)
- Other / miscellaneous	1 (1% of 138)	1 (8)
**Direct non-medical costs (total)**	**34 (16% of 219)**	**34 (34)**
- Transport to hospital	7 (21% of 34)	7 (17)
- Food	20 (59% of 34)	21 (20)
- Accommodation	0 (0% of 34)	0 (0)
- Other*	7 (21% of 34)	6 (10)
**Indirect costs**		
- Lost wages	46 (100% of 46)	35 (116)
**TOTAL OOP COSTS**	**243**	**176 (165)**

*other relates to travel and other associated costs incurred as a result for needed investigations from and or medication / supplies from an external facility. SPSS calculates only the mean using imputed variables, hence no standard deviation is displayed.

For peer review only

Appendix table 4: Route of payment for OOP costs; percentage of the total OOP costs by cost categories paid to bank / cashier, directly to staff or externally for different services accessed once tertiary level care was reached

Costs	% paid to Hospital bank / cashier	% paid directly to or via staff	% paid externally	% unknown	Total
**TOTAL in-hospital costs**	**32.64%**	**48.16%**	**16.50%**	**2.70%**	**100%**
**Administration**	**52.03%**	**42.53%**	-	5.44%	100%
- Registration fees	90.98%	8.71%	-	0.30%	100%
- Admission fees	66.78%	32.39%	-	0.83%	100%
- Triage fees	8.05%	91.95%	-	0.00%	100%
- Bed fees	19.47%	46.13%	-	34.40%	100%
- Discharge fees	41.19%	57.28%	-	1.53%	100%
**Investigations**	**19.39%**	**44.29%**	**33.73%**	**2.59%**	**100%**
- Laboratory	25.68%	40.29%	30.13%	3.90%	100%
- Imaging	14.43%	47.44%	36.57%	1.56%	100%
**Total operation costs**	**55.40%**	**32.89%**	**9.35%**	**2.35%**	**100%**
- Operation	80.10%	18.91%	-	1.00%	100%
- Medical supplies for the operation	13.42%	56.59%	25.31%	4.68%	100%
- Other / miscellaneous	69.77%	30.23%	-	0.00%	100%
**Blood transfusion**	**16.24%**	**67.68%**	**13.85%**	**2.23%**	**100%**
**Total medications and medical supplies for ward care**	**4.82%**	**61.00%**	**31.73%**	**2.45%**	**100%**
- Medications	2.19%	62.86%	31.91%	3.03%	100%
- Medical supplies	10.81%	56.74%	31.31%	1.14%	100%
**Informal payment**	**2.44%**	**97.56%**	**-**	**0.00%**	**100%**
- Doctors' fees	8.72%	91.28%	-	0.00%	100%
- Nursing care	0%	100%	-	0.00%	100%
- Porters	0%	100%	-	0.00%	100%
- Tips	0%	100%	-	0.00%	100%
**Other / miscellaneous costs**	**0%**	**98.37%**	**0**	**1.63%**	**100%**

For peer review only - http://bmjopen.bmj.com/site/about/guidelines.xhtml

Appendix table 5: Linear regression analysis showing odds of increasing in-hospital costs of care for each variable using a generalized linear model with imputed variables using a Tweedie 1.9 function.

Variable		Odds Ratio	95% CI	p-value
Sex	Female	ref		
	Male	1.05	(0.86-1.29)	0.63
Age	Age	1.02	(1.01-1.02)	0.00
Length of stay	Length of stay	1.02	(1.02 -1.03)	0.00
Type of admission	Elective admission	ref		
	Emergency admission	0.96	(0.75-1.24)	0.77
Category of operation	Non-operative	ref		
	Burns	1.33	(0.25-7.00)	0.74
	ENT	0.64	(0.36-1.166)	0.14
	General surgery	1.67	(1.29-2.17)	0.00
	General paediatric surgery	0.84	(0.57-1.24)	0.38
	Trauma and orthopaedic	1.30	(0.98- 1.74)	0.07
	Urology	2.08	(1.22-3.53)	0.01
Area of residence	Rural	ref		
	Urban	0.98	(0.76-1.25)	0.85

Appendix table 6: Study questionnaire

Section 1: Demographics and admission questions

These questions are to be answered in conjunction with the patients notes and screening/recruitment sheet

Code	Question	Response
A1	Participant **unique ID**:	
A2	**Discharge** details	Care episode complete and discharged by Dr ☐<br>Transfer to another healthcare facility ☐<br>Self-discharging ☐ If ticked state reason_________________
A3	Where was the patient **admitted from**?	Connaught
		Direct to trauma ward / A&E / OPD ☐<br>Referral ☐ specify _______________<br>SOP (specialist outpatients) ☐<br>Through direct contact ☐<br>Transfer from the medical ward ☐<br>Other ☐ specify ______________
A4	Where is the patient being **discharged from**?	Connaught
		Trauma ward / A&E / OPD ☐<br>Surgical ward ☐<br>Annexe ☐<br>Other ☐ specify ___________
A5	**Age** of patient	_______________ / Don't know ☐ / Adult (AD) ☐
A6	**Sex**	Male ☐ Female ☐
A7	Usual **residential location** (address including district and from that state if urban or rural)	Address: ______________________________________<br>Urban ☐ Rural ☐

For peer review only - http://bmjopen.bmj.com/site/about/guidelines.xhtml

A8	Does the patient meet any exemption criteria for **free treatment**?	Yes ☐ No ☐<br>If yes, state which criteria (tick as applicable)<br>Aged under 5 years ☐<br>Pregnant ☐<br>Lactating ☐<br>Ebola survivor ☐<br>Destitute ☐<br>Disabled ☐
A9	**Type of admission**	Emergency (e.g trauma ward) ☐ Elective ☐
A10	**Primary diagnosis**	______________________________
A11	What **treatment** did the patient receive? *(Note: Procedures are often done in the Trauma ward (minors procedure room) e.g skin traction/POP/suturing)*	**Operation/ procedure** ☐ (specify and go to A14)<br>Specify ______________________________<br>**Non-operative** surgical care ☐ (go to A13)
A12	If patient received **non-operative care** state reason	Clinically appropriate / not recommended by Dr ☐<br>Patient chose not to have an operation / procedure ☐<br>Financial (unable to pay) ☐<br>Other ☐ (please give a brief statement)<br>______________________________
A13	**Length of hospital stay** (admission and discharge date)	**Admission date:** _ _/_ _/_ _ _ _**Discharge date:**_ _/_ _/_ _ _ _

Section 2a: Household structure and typical household income and expenditure

All questions are to be answered by the patient, parent, guardian or household member or head. ***Encourage patient to invite household head / member / breadwinner or the person who deals with household expenditure and or has made the payments for the care received - to help answer the questions.***

"Firstly are a few questions to understand the structure of your household (the people that eat food from the same pot and take instructions from the same head (excluding lodgers / individuals that pay to live in your house) and the average household income"

Code	Question	Response
B1	**Who** is being interviewed? Tick all that are applicable<br><br>If questions are being answered by someone other than the patient (e.g. household member), what is their relationship to the patient?	Patient ☐<br>Parent / Guardian ☐<br>Household head ☐<br>Other ☐ (State relationship to patient)<br>______________________
B2	What is the **size of your household**, including yourself how many people normally eat food from the same pot and take instructions from the same head (exclude lodgers / individuals that pay to live in your house)	______________
B3	Does **anyone in your household (household head/members) generate income**?	Yes ☐ (go to B4) No ☐ (go to B7)
B4	**How many people** in your household **generate income** that is used to support the household?	______________<br>Don't know ☐
B5	What is the **occupation** of the person **who contributes the most** to your household expenses?	______________<br>Salaried ☐ Non-salaried ☐<br>Don't know ☐
B6	**How much income does your household generate** (in total) to support the household in a typical month?	__________Leones Don't know ☐
B7	What is the **highest level of education of the main breadwinner (i.e the individual identified in B5)?**	No formal education ☐<br>Primary ☐<br>Secondary ☐<br>College ☐<br>University ☐<br>Other ☐ specify ____________
B8	Does your household **have any of the following**?	Electricity / Light Yes ☐ No ☐<br>Mobile phone Yes ☐ No ☐<br>Radio Yes ☐ No ☐<br>Television (TV) Yes ☐ No ☐<br>Computer Yes ☐ No ☐<br>Refrigerator Yes ☐ No ☐

For peer review only - http://bmjopen.bmj.com/site/about/guidelines.xhtml

		Generator Yes ☐ No ☐<br>Bicycle Yes ☐ No ☐<br>Honda/ Motorcycle Yes ☐ No ☐<br>Car or truck Yes ☐ No ☐
B9	What material is used for the **roof of your house**?	Natural roofing (thatch) ☐<br>Basic roofing (tarpoline, metal, zinc) ☐<br>Finished roof (concrete / tiled) ☐<br>Other ☐ specify _______________
B10	What material is used for the **floor of your house**?	Natural floor (mud/earth/wattle) ☐<br>Basic floor (wood/cement) ☐<br>Finished floor (concrete/tile/carpet) ☐<br>Other ☐ specify _______________
B11	What material is used for the **walls of your house**?	Natural walls (mud/earth/wattle) ☐<br>Basic walls (stone/mud bricks/zinc) ☐<br>Finished walls (concrete) ☐<br>Other ☐ specify _______________

Section 2b: Typical household expenditure

"I would now like to ask you some questions about your household expenses starting with how much your household spends on food and consumption in a typical week"

Code	Question	Response
C1	Does the month of **Ramadhan** effect your household spending/expenses?	Yes ☐ No ☐<br>**If yes, say: please answer all the questions on expenses based on a week / month outside of the Ramadhan period**
C2	How much does your household spend on **food (including chop money)**?	_________________Leones Don't know ☐
C3	How much does your household spend **drinks** (non-alcoholic drinks such as water, tea, coffee, milk and soft drinks?	_________________Leones Don't know ☐
C4	**Tobacco and alcoholic beverages** (including beer, wine, spirits, poyo) – had at home / outside	_________________Leones Don't know ☐
C5	Food eaten outside the dwelling (for example, at vendors, **cookery**, kiosks or restaurants)	_________________Leones Don't know ☐
C6	**Other food** items (e.g kola nut, food not included in chop money)	_________________Leones Don't know ☐
C7	Communication fees, including megabites (internet), mobile phone (**credit /top up)** and others?	_________________Leones Don't know ☐
C8	**Transportation** (to work place, market, school etc)? (For example, petrol, taxis, motorbike taxis)	_________________Leones Don't know ☐

"I would now like to ask you about other expenses your household might have had in the last month or a typical month"

C9	**Utilities, such as water, light, electricity (NPA), waste disposal,** etc.?	_________________Leones Don't know ☐
C10	**Fuel (e.g cooking / generator** - gas, coal, kerosene, firewood, petrol, diesel, etc.)?	_________________Leones Don't know ☐
C11	**Personal toiletries** and personal care (e.g.soap, toothpaste, toothbrush, toilet roll, cosmetics, beauty salon, getting hair done etc.)?	_________________Leones Don't know ☐
C12	**Clothing** and bedding?	_________________Leones Don't know ☐
C13	**Loan repayments** (e.g on your house) / other **debt** or **microcredit** for business or other purposes?	_________________Leones Don't know ☐
C14	**Entertainment**, including; cinemas/video centres to watch football matches, games, stadium for shows, soccer matches, or hangouts / chilling?	_________________Leones Don't know ☐
C15	Payments for **household help**/servants, including cook, maid, driver, security, gardener, etc.?	_________________Leones Don't know ☐
C16	How much does your household pay in **remittance** to family living away from home (**money, airtel/Africell money**, food etc)?	_________________Leones Don't know ☐
C17	Excluding this hospital stay, how much money does your household usually spend on health care related to **"white man medicine",** including medicines, fees for doctors' appointments and hospital visits?	_________________Leones Don't know ☐
C18	Excluding this hospital stay, how much money does your household usually spend on health care related to **"country/black man medicine"**?	_________________Leones Don't know ☐
C19	Do you have any **other monthly expenditures**?	Yes ☐ Specify ___________________________<br>No ☐ (go to C21)

For peer review only - http://bmjopen.bmj.com/site/about/guidelines.xhtml

C20	**How much do these other expenditures** amount to?	________________Leones Don't know ☐
"I know these questions may be difficult to answer but try to give me the best estimate of expenses. I would now like you to focus on household expenses **over the last 12 months**. These are expenses that may be more periodic or "big purchases"		
C21	**Rent** (per year)	________________Leones Don't know ☐
C22	**Education fees and school supplies** (tuition, course fees, books)?	________________Leones Don't know ☐
C23	**Electrical goods** (mobile phones, televisions, DVD, radio, refrigerators,)?	________________Leones Don't know ☐
C24	**Big purchases** such as;<br>**Furniture** (tables, chairs, beds, sleeping mats)<br>**Vehicles** (trucks, cars, motorcycles, keke, bicycles) and **upkeep/repairs**?<br>**Tools** (brooms, nails, hammer, paint, shovel, gardening equipment etc)	 ________________Leones Don't know ☐<br>________________Leones Don't know ☐<br>________________Leones Don't know ☐
C25	**Hosting rituals or ceremonies** (funerals, birthdays, wedding, naming ceremony, pray de close, Christmas, the Haj)?	________________Leones Don't know ☐
C26	**Gifts for ceremonies** (funerals, birthdays, wedding, naming ceremony, pray de close, Christmas) if invited?	________________Leones Don't know ☐
C27	**Donations or contributions** to religious organisations (e.g. church/mosque)	________________Leones Don't know ☐
C28	**Deposit / upfront payments for property or land** (excluding monthly loans)?	________________Leones Don't know ☐
C29	Cleaning services or repair service (**house maintenance**)?	________________Leones Don't know ☐
C30	**Livestock** (chicken, goat, sheep etc)?	________________Leones Don't know ☐
C31	**Taxes** (city rate for home owner, vehicle tax, city council tax, NASSIT (national social security insurance trust)?	________________Leones Don't know ☐
C32	**Health insurance** premiums (including, community health insurance schemes) or pre-paid health plans?	________________Leones Don't know ☐

Section 3: Out-of-pocket payments for care sought prior to admission (from the day they fell ill with this illness)
"I would now like to ask you about all the health costs to your household before coming to hospital from when you fell sick with this illness / problem"

Code	Question	Response
D1	**When did you fall sick with the illness** that meant you had to be admitted to hospital?	______days before admission<br>______weeks before admission<br>______months before admission<br>Don't know ☐ (go to section 3 (C1)
"I would now like you to think about that period of time; **from falling sick to getting to the point of decision to come to Connaught / PCMH.** The following questions will be related to that time. I know that it may be difficult to think that far back but please give the best estimate you can of the costs"		
D2	Did you seek care for this illness elsewhere **before coming to this hospital**?	Yes ☐ (go to D3) No ☐ (go to section 4 (E1))
D3	**Where did you go** to get care for your illness before coming to this hospital? Tick all that are applicable<br><br>For each type of care provider ticked from the list below ask about the **number of visits** and a **cost break down**. If the patient cannot give a breakdown of costs ask for an **estimated total**<br>***Guidance notes;***<br>***Consultation -*** *fees to the provider ("doctors' fees"), fees excluding investigations and tests*<br>***Medications***<br>***Medical supplies*** *- Consumables, gloves, bandages, dressings etc*<br>***Investigations*** *– Includes labs, scans, x-rays and other imaging*<br>***Transportation*** *- transport for getting to and from the facilities, subsequent investigations/ getting treatments / medicines*	
	**Private clinic** ☐ no. of visits ___<br>How much was spent on;<br>Consultation ________________Leones Don't know ☐<br>Medications ________________Leones Don't know ☐<br>Medical supplies ______________Leones Don't know ☐<br>Investigations ________________Leones Don't know ☐<br>Transportation ______________Leones Don't know ☐<br>Other ____________________Leones Don't know ☐<br>**OR** Estimated total ____________Leones Don't know ☐<br><br>**Charity or religious clinic** ☐ no. of visits ___<br>How much was spent on;	**Other government hospital** ☐ no. of visits ___<br>How much was spent on;<br>Consultation ________________Leones Don't know ☐<br>Medications ________________Leones Don't know ☐<br>Medical supplies ______________Leones Don't know ☐<br>Investigations ________________Leones Don't know ☐<br>Transportation ______________Leones Don't know ☐<br>Other ____________________Leones Don't know ☐<br>**OR** Estimated total ____________Leones Don't know ☐<br><br>**Private hospital** ☐ no. of visits ___<br>How much was spent on;

For peer review only

Consultation ____________ Leones Don't know ☐
Medications ____________ Leones Don't know ☐
Medical supplies ____________ Leones Don't know ☐
Investigations ____________ Leones Don't know ☐
Transportation ____________ Leones Don't know ☐
Other ____________ Leones Don't know ☐
OR Estimated total ________ Leones Don't know ☐

Pharmacy, drug peddler, pepper Dr. ☐ no. of visits ___
How much was spent on;
Consultation ____________ Leones Don't know ☐
Medications ____________ Leones Don't know ☐
Medical supplies ____________ Leones Don't know ☐
Investigations ____________ Leones Don't know ☐
Transportation ____________ Leones Don't know ☐
Other ____________ Leones Don't know ☐
OR Estimated total ________ Leones Don't know ☐

Traditional Healer / Herbalist ☐ no. of visits ___
How much was spent on;
Consultation ____________ Leones Don't know ☐
Medications ____________ Leones Don't know ☐
Medical supplies ____________ Leones Don't know ☐
Investigations ____________ Leones Don't know ☐
Transportation ____________ Leones Don't know ☐
Other ____________ Leones Don't know ☐
OR Estimated total ________ Leones Don't know ☐

Traditional Birth Attendant (TBA) /Community Health Worker came home ☐ no. of visits ___
How much was spent on;
Consultation ____________ Leones Don't know ☐
Medications ____________ Leones Don't know ☐
Medical supplies ____________ Leones Don't know ☐
Investigations ____________ Leones Don't know ☐
Transportation ____________ Leones Don't know ☐
Other ____________ Leones Don't know ☐
OR Estimated total ________ Leones Don't know ☐

Home delivery ☐ no. of visits _____
How much was spent on;
Consultation ____________ Leones Don't know ☐
Medications ____________ Leones Don't know ☐
Medical supplies ____________ Leones Don't know ☐
Investigations ____________ Leones Don't know ☐
Transportation ____________ Leones Don't know ☐
Other ____________ Leones Don't know ☐
OR Estimated total ________ Leones Don't know ☐

Consultation ____________ Leones Don't know ☐
Medications ____________ Leones Don't know ☐
Medical supplies ____________ Leones Don't know ☐
Investigations ____________ Leones Don't know ☐
Transportation ____________ Leones Don't know ☐
Other ____________ Leones Don't know ☐
OR Estimated total ________ Leones Don't know ☐

Community health clinic (Cheifdom level) ☐
no. of visits ___
How much was spent on;
Consultation ____________ Leones Don't know ☐
Medications ____________ Leones Don't know ☐
Medical supplies ____________ Leones Don't know ☐
Investigations ____________ Leones Don't know ☐
Transportation ____________ Leones Don't know ☐
Other ____________ Leones Don't know ☐
OR Estimated total ________ Leones Don't know ☐

Community health post ☐ no. of visits ___
How much was spent on;
Consultation ____________ Leones Don't know ☐
Medications ____________ Leones Don't know ☐
Medical supplies ____________ Leones Don't know ☐
Investigations ____________ Leones Don't know ☐
Transportation ____________ Leones Don't know ☐
Other ____________ Leones Don't know ☐
OR Estimated total ________ Leones Don't know ☐

Maternity Child Health Post (MCHP) ☐ no. of visits ___
How much was spent on;
Consultation ____________ Leones Don't know ☐
Medications ____________ Leones Don't know ☐
Medical supplies ____________ Leones Don't know ☐
Investigations ____________ Leones Don't know ☐
Transportation ____________ Leones Don't know ☐
Other ____________ Leones Don't know ☐
OR Estimated total ________ Leones Don't know ☐

Other ☐ specify ______________ no. of visits ___
How much was spent on;
Consultation ____________ Leones Don't know ☐
Medications ____________ Leones Don't know ☐
Medical supplies ____________ Leones Don't know ☐
Investigations ____________ Leones Don't know ☐
Transportation ____________ Leones Don't know ☐
Other ____________ Leones Don't know ☐
OR Estimated total ________ Leones Don't know ☐

Section 4: Out-of-pocket payments related to hospital admission
"I would now like to ask you about <u>all the costs to your household from travel to the hospital to admission, your whole hospital stay and treatment through to discharge</u>"

Please reiterate that as with all questions any questions related to informal payments / payments directly to staff are completely anonymous and no names are recorded or required. The purpose is to understand how much patients have to pay for <u>all</u> types of costs associated with their hospital admission so the following questions should be answered without undue concern.

If the household did not incur any of the expenses listed please state 0 Leones (this applies throughout the questionnaire)

Code	Question	Response

For peer review only - http://bmjopen.bmj.com/site/about/guidelines.xhtml
For peer review only

E1	Once the referral or decision was made to come to Connaught/PCMH, **how did you and all the attendants come / travel**?	Ambulance ☐<br>Public transport - Taxi / KK /Okada ☐<br>Private transport ☐<br>Other ☐ specify ________________
E2	How much did this **transport cost?**	__________Leones Don't know ☐
E3	Did you have to pay any money for **registration**? If yes, ask how much the household paid for registration fees?<br><br>Tick all that are applicable	__________Leones Don't know ☐<br>Who or where was that paid to;<br>- Bank in Connaught ☐<br>__________Leones<br>- Directly to hospital staff ☐<br>__________Leones<br>- Other ☐ specify __________<br>__________Leones
E4	Did you have to pay any money for **admission** (fees / booklet)? If yes, ask how much the household paid for admission?<br><br>Tick all that are applicable	__________Leones Don't know ☐<br>Who or where was that paid to;<br>- Bank in Connaught ☐<br>__________Leones<br>- Directly to hospital staff ☐<br>__________Leones<br>- Other ☐ specify __________<br>__________Leones
E5	Did you have to pay any money at or for **triage (e.g for blood sugar)**? If yes, ask how much the household paid?<br><br>Tick all that are applicable	__________Leones Don't know ☐<br>Who or where was that paid to;<br>- Bank in Connaught ☐<br>__________Leones<br>- Directly to hospital staff ☐<br>__________Leones<br>- Other ☐ specify __________<br>- __________Leones
E6	Did you have to pay any money for any **helpers** (e.g porters, wheelchair, hospital staff to help you get around etc). If yes, ask how much the household paid?	__________Leones Don't know ☐
E7	Did you have to pay any **doctors' fees / consultation fees**? If yes, ask how much the household paid in doctors' fees / consultation fees?<br><br>Tick all that are applicable	__________Leones Don't know ☐<br>Who or where was that paid to;<br>- Bank in Connaught ☐<br>__________Leones<br>- Directly to healthcare workers ☐<br>__________Leones<br>- Other ☐ specify __________<br>__________Leones
E8	Did you have any **laboratory (lab) tests**? If yes, ask how much money the household spent on these tests?<br><br>Tick all that are applicable	__________Leones Don't know ☐<br>Who or where was that paid to;<br>- Bank in Connaught ☐<br>__________Leones<br>- External lab ☐<br>__________Leones<br>- Internal lab ☐<br>__________Leones<br>- Directly to healthcare workers ☐<br>__________Leones<br>- Other ☐ specify __________<br>__________Leones
E9	Did you have any **x-rays, scans or imaging**? If yes, ask how much money your household spent on scans, imaging and x-rays?<br><br>Tick all that are applicable	__________Leones Don't know ☐<br>Who or where was that paid to;<br>- Bank in Connaught ☐<br>__________Leones<br>- External facility ☐<br>__________Leones<br>- Internal facility ☐<br>__________Leones

For peer review only - http://bmjopen.bmj.com/site/about/guidelines.xhtml

		- Directly to healthcare workers ☐ ________________Leones<br>- Other ☐ specify ____________________ ________________Leones
E10	Did the patient have an **operation or procedure** (e.g skin traction/POP/suturing) in hospital?	Yes ☐ No ☐ (go to E15)
E11	How much did you pay for this **operation or procedure**?<br><br>Tick <u>all</u> that are applicable	___________________Leones. Don't know ☐<br>Who or where was that paid to;<br>- Bank in Connaught ☐ ________________Leones<br>- Directly to healthcare workers ☐ ________________Leones<br>- Other ☐ specify ____________________ ________________Leones
E12	Did you have to pay for **medications or medical supplies** (e.g. cannulas, IV lines, gauze, bandages, dressings, gloves, catheters and other consumables) **for theatre / your operation/procedure**? If yes, how much money did your household spend on these medicines and medical supplies?<br><br>Tick <u>all</u> that are applicable	___________________Leones. Don't know ☐<br>Who or where was that paid to;<br>- Bank in Connaught ☐ ________________Leones<br>- External pharmacy ☐ ________________Leones<br>- Internal pharmacy / cost recovery ☐ ________________Leones<br>- Directly to healthcare workers ☐ ________________Leones<br>- Other ☐ specify ____________________ ________________Leones
E13	Did you have any **extra charges once in the operating room /area**? Please specify what this was for.	Yes ☐ (specify) No ☐ (go to E15)<br>**Specify what this was for?**<br>____________________________________
E14	How much money did your household spend on these **extra-charges in the operating room?**<br><br>Tick <u>all</u> that are applicable	___________________Leones. Don't know ☐<br>Who or where was that paid to;<br>- Bank in Connaught ☐ ________________Leones<br>- External pharmacy ☐ ________________Leones<br>- Internal pharmacy / cost recovery ☐ ________________Leones<br>- Directly to healthcare workers ☐ ________________Leones<br>- Other ☐ specify ____________________ ________________Leones
E15	Did your household have to arrange and get **blood for transfusion**?	Yes ☐ No ☐ (go to E18)
E17	How much money did your household spend on **blood transfusion related costs** (including blood bank fees, supplies, paying a donor etc.)<br><br>Tick <u>all</u> that are applicable	___________________Leones Don't know ☐<br>Who or where was that paid to;<br>- Bank in Connaught ☐ ________________Leones<br>- External facility ☐ ________________Leones<br>- Internal facility ☐ ________________Leones<br>- Directly to healthcare workers ☐ ________________Leones<br>- Other ☐ specify ____________________ ________________Leones
E18	Did you have to pay for any **medications in A&E/OPD or on the ward** (excluding prescriptions specifically for theatre)? If yes, ask how much the household paid for this?<br><br>Tick <u>all</u> that are applicable	___________________Leones Don't know ☐<br>Who or where was that paid to;<br>- Bank in Connaught ☐ ________________Leones<br>- External pharmacy ☐ ________________Leones<br>- Internal pharmacy / cost recovery ☐

For peer review only - http://bmjopen.bmj.com/site/about/guidelines.xhtml

		______________Leones<br>- Directly to healthcare workers ☐<br>______________Leones<br>- Other ☐ specify ________________<br>______________Leones
E19	Did you have to pay for any **medical supplies in A&E/OPD and the Ward** (not including medications, e.g. cannulas, IV lines, gauze, bandages, dressings, gloves, catheters and other consumables) (excluding the prescription for theatre))? If yes, ask how much was paid?<br><br>Tick all that are applicable	________________Leones. Don't know ☐<br>Who or where was that paid to;<br>- Bank in Connaught ☐<br>______________Leones<br>- External pharmacy / facility ☐<br>______________Leones<br>- Internal pharmacy / facility / cost recovery ☐<br>______________Leones<br>- Directly to healthcare workers ☐<br>______________Leones<br>- Other ☐ specify ________________<br>______________Leones
E20	Did you have to pay any **bed fees** (excluding admission fees / book? If yes, ask how much money the household paid in bed fees?<br><br>Tick all that are applicable	______________Leones Don't know ☐<br>Who or where was that paid to;<br>- Bank in Connaught ☐<br>______________Leones<br>- Directly to healthcare workers ☐<br>______________Leones<br>- Other ☐ specify ________________<br>______________Leones
E21	Did your household pay any money for **nursing care** (**requested** by nurses for care such as dressing changes etc, not including medications or medical supplies)? If yes, ask how much was paid?	______________Leones Don't know ☐<br>Who or where was that paid to;<br>- Bank in Connaught ☐<br>______________Leones<br>- Directly to healthcare workers ☐<br>______________Leones<br>- Other ☐ specify ________________<br>______________Leones
E22	Did your household give any **tips (ahjo)** i.e. voluntary and not requested money / gifts to healthcare workers / staff? If yes ask how much this amounted to?	________________Leones Don't know ☐
E23	How many **family members / attendants / persons stayed in hospital** during your hospital stay (excluding visitors)?	______________________people
E24	**Where did they stay** and **how much** did your household pay towards this?	In hospital ☐<br>Outside accommodation ☐<br>Other ☐ specify ______________________<br>Total cost____________Leones Don't know ☐
E25	During your hospital stay **where did you get food from**?<br><br>Tick all that are applicable	Provided by the hospital ☐<br>Bought by the patient / household ☐<br>Brought for the patient by someone else ☐<br>Other ☐ specify ____________________________
E26	How much did you / your household **spend on food** during your hospital stay? If they can't give a break down then ask for a total.	On patient ______________Leones Don't know ☐<br>For attendants ___________Leones Don't know ☐<br>Total ______________Leones Don't know ☐
E27	Did your household spend any money on transport for all the **"running" (transport to get food, hospital supplies, investigations and medications externally, excluding transport for visitors)**? If yes ask how much and state what this was for?<br>Tick all that are applicable	________________Leones Don't know ☐<br>**What was this for?**<br>Food for the patients / attendants ☐<br>Going for tests / investigations outside ☐<br>Getting medicines / medical supplies ☐<br>Other ☐ specify ______________
E28	Did your household have to pay any money at the time of **discharge**? If yes, ask how much was paid?	________________Leones. Don't know ☐<br>Who or where was that paid to;<br>- Bank in Connaught ☐<br>______________Leones<br>- Directly to healthcare workers ☐<br>______________Leones

For peer review only - http://bmjopen.bmj.com/site/about/guidelines.xhtml

		- Other ☐ specify ________________ ________________Leones
E29	Did your household have **any other illness related / hospital costs (e.g at SOP / other departments visited)**? If yes, specify what these costs where for.	Yes ☐ (specify and go to E30) No ☐ (go to F1)<br>**Specify**________________________________
E30	How much did these **other costs** amount to?	________________Leones. Don't know ☐<br>Who or where was that paid to;<br>- Bank in Connaught ☐ ________________Leones<br>- Directly to healthcare workers ☐ ________________Leones<br>- Other ☐ specify ________________ ________________Leones

Section 5: Indirect non-medical expenditure and how costs are met
"I will now ask you some questions about the broader implications of the costs and how you have met these costs?"

Code	Question	Response
F1	Do you have **health insurance/medical care** to cover any medical costs?	Yes ☐ No ☐ (go to F5)<br>Don't know ☐
F2	What **type of health insurance** do you have?	☐ Private insurance<br>☐ Work-related insurance<br>☐ Other (specify)____________________
F3	What is your **monthly contribution towards your insurance** (if applicable)?	________________Leones Don't know ☐
F4	Does **this insurance cover all (total) medical costs?**	Yes ☐ No ☐ If no, how much was paid in addition ____________Leones Don't know ☐
F5	**How did your household pay** for all the above costs? Tick all that applicable	
	**Use savings**? Yes ☐ No ☐ Don't know ☐	How much? ________________Leones Don't know ☐
	**Borrow money**? Yes ☐ No ☐ Don't know ☐	How much?________________Leones<br>Does this need to be paid back? Yes ☐ No ☐ Don't know ☐
	**Arrange family contributions?** Yes ☐ No ☐ Don't know ☐	How much?________________Leones<br>Does this need to be paid back? Yes ☐ No ☐ Don't know ☐
	**Charity money from family/friends outside the country/church/social clubs?** Yes ☐ No ☐ Don't know ☐	How much?________________Leones<br>Does this need to be paid back? Yes ☐ No ☐ Don't know ☐
	**Pledge / pawn any possessions** (including livestock, assets including electrical goods, generator, watches, jewellery etc.)?<br>Yes ☐ No ☐ Don't know ☐	How much money did this raise?________________Leones<br>Don't know ☐
	**Sell any possessions or land?** Yes ☐ No ☐ Don't know ☐	How much money did this raise?________________Leones<br>Don't know ☐
	**Any other way of meeting the hospital costs?**<br>Yes ☐ No ☐ Don't know ☐	Specify how ____________________________<br>How much money did this raise?________________Leones<br>Don't know ☐
F6	Did your household have to **stop sending any children to school, or pay reduced school fees** in order to pay for this hospitalisation?	Yes ☐ No ☐ Don't know ☐
F7	Did your household **loose any wages** due to this hospital stay? If yes, ask how much was lost?	Yes ☐ No ☐ Don't know ☐<br>How much was lost ________________Leones<br>Don't know ☐
F8	Did you or anyone in your household **lose their job or change their role at work or home?**	Yes ☐ No ☐ Don't know ☐<br>If yes please specify<br>Lost job ☐<br>Took up employment / 2nd job ☐<br>Change of duties in household to meet costs ☐<br>Other ☐ specify ____________________________

For peer review only - http://bmjopen.bmj.com/site/about/guidelines.xhtml

Any other comments?

For peer review only

For peer review only - http://bmjopen.bmj.com/site/about/guidelines.xhtml

STROBE Statement—Checklist of items that should be included in reports of ***cross-sectional studies***

	Item No	Recommendation	Page number
**Title and abstract**	1	(*a*) Indicate the study's design with a commonly used term in the title or the abstract	1-3
		(*b*) Provide in the abstract an informative and balanced summary of what was done and what was found	1-3
**Introduction**			**4**
Background/rationale	2	Explain the scientific background and rationale for the investigation being reported	4
Objectives	3	State specific objectives, including any prespecified hypotheses	4
**Methods**			**5-7**
Study design	4	Present key elements of study design early in the paper	5
Setting	5	Describe the setting, locations, and relevant dates, including periods of recruitment, exposure, follow-up, and data collection	5
Participants	6	(*a*) Give the eligibility criteria, and the sources and methods of selection of participants	5
Variables	7	Clearly define all outcomes, exposures, predictors, potential confounders, and effect modifiers. Give diagnostic criteria, if applicable	5-7
Data sources/ measurement	8*	For each variable of interest, give sources of data and details of methods of assessment (measurement). Describe comparability of assessment methods if there is more than one group	*5-7*
Bias	9	Describe any efforts to address potential sources of bias	10-11
Study size	10	Explain how the study size was arrived at	7
Quantitative variables	11	Explain how quantitative variables were handled in the analyses. If applicable, describe which groupings were chosen and why	7
Statistical methods	12	(*a*) Describe all statistical methods, including those used to control for confounding	7
		(*b*) Describe any methods used to examine subgroups and interactions	7
		(*c*) Explain how missing data were addressed	7
		(*d*) If applicable, describe analytical methods taking account of sampling strategy	n/a
		(*e*) Describe any sensitivity analyses	7
**Results**			**8-9**
Participants	13*	(a) Report numbers of individuals at each stage of study—eg numbers potentially eligible, examined for eligibility, confirmed eligible, included in the study, completing follow-up, and analysed	8-9 Figure 1
		(b) Give reasons for non-participation at each stage	Figure 1
		(c) Consider use of a flow diagram	Figure 1
Descriptive data	14*	(a) Give characteristics of study participants (eg demographic, clinical, social) and information on exposures and potential confounders	8 Table 1
		(b) Indicate number of participants with missing data for each variable of interest	Table 1
Outcome data	15*	Report numbers of outcome events or summary measures	8-9 Table 1 Table 2

For peer review only

			Table 3<br>Table 4
Main results	16	(*a*) Give unadjusted estimates and, if applicable, confounder-adjusted estimates and their precision (eg, 95% confidence interval). Make clear which confounders were adjusted for and why they were included	n/a
		(*b*) Report category boundaries when continuous variables were categorized	8-9<br>Tables
		(*c*) If relevant, consider translating estimates of relative risk into absolute risk for a meaningful time period	n/a
Other analyses	17	Report other analyses done—eg analyses of subgroups and interactions, and sensitivity analyses	Appendix<br>1, 2, 3
**Discussion**			**9 - 10**
Key results	18	Summarise key results with reference to study objectives	9-10
Limitations	19	Discuss limitations of the study, taking into account sources of potential bias or imprecision. Discuss both direction and magnitude of any potential bias	10-11
Interpretation	20	Give a cautious overall interpretation of results considering objectives, limitations, multiplicity of analyses, results from similar studies, and other relevant evidence	9-10
Generalisability	21	Discuss the generalisability (external validity) of the study results	10
**Other information**			
Funding	22	Give the source of funding and the role of the funders for the present study and, if applicable, for the original study on which the present article is based	1

*Give information separately for exposed and unexposed groups.

Note: An Explanation and Elaboration article discusses each checklist item and gives methodological background and published examples of transparent reporting. The STROBE checklist is best used in conjunction with this article (freely available on the Web sites of PLoS Medicine at http://www.plosmedicine.org/, Annals of Internal Medicine at http://www.annals.org/, and Epidemiology at http://www.epidem.com/). Information on the STROBE Initiative is available at www.strobe-statement.org.

For peer review only

What is the financial burden to patients of accessing surgical care in Sierra Leone? A cross-sectional survey of catastrophic and impoverishing expenditure

Journal:	*BMJ Open*
Manuscript ID	bmjopen-2020-039049.R2
Article Type:	Original research
Date Submitted by the Author:	26-Oct-2020
Complete List of Authors:	Phull, Manraj ; West Hertfordshire Hospitals NHS Trust<br>Grimes, Caris; King's College London Faculty of Life Sciences and Medicine; Medway NHS Foundation Trust<br>Kamara, Thaim; University of Sierra Leone College of Medicine and Allied Health Sciences, Surgery<br>Wurie, Haja ; University of Sierra Leone College of Medicine and Allied Health Sciences<br>Leather, Andy; King's College London Faculty of Life Sciences and Medicine, King's Centre for Global Health<br>Davies, Justine ; King's College London Faculty of Life Sciences and Medicine, Centre for Global Health; University of Birmingham Institute of Applied Health Research
**Primary Subject Heading**:	Health economics
Secondary Subject Heading:	Global health, Health policy, Surgery
Keywords:	SURGERY, HEALTH ECONOMICS, Health policy < HEALTH SERVICES ADMINISTRATION & MANAGEMENT

SCHOLARONE™
Manuscripts

I, the Submitting Author has the right to grant and does grant on behalf of all authors of the Work (as defined in the below author licence), an exclusive licence and/or a non-exclusive licence for contributions from authors who are: i) UK Crown employees; ii) where BMJ has agreed a CC-BY licence shall apply, and/or iii) in accordance with the terms applicable for US Federal Government officers or employees acting as part of their official duties; on a worldwide, perpetual, irrevocable, royalty-free basis to BMJ Publishing Group Ltd ("BMJ") its licensees and where the relevant Journal is co-owned by BMJ to the co-owners of the Journal, to publish the Work in this journal and any other BMJ products and to exploit all rights, as set out in our licence.

The Submitting Author accepts and understands that any supply made under these terms is made by BMJ to the Submitting Author unless you are acting as an employee on behalf of your employer or a postgraduate student of an affiliated institution which is paying any applicable article publishing charge ("APC") for Open Access articles. Where the Submitting Author wishes to make the Work available on an Open Access basis (and intends to pay the relevant APC), the terms of reuse of such Open Access shall be governed by a Creative Commons licence – details of these licences and which Creative Commons licence will apply to this Work are set out in our licence referred to above.

Other than as permitted in any relevant BMJ Author's Self Archiving Policies, I confirm this Work has not been accepted for publication elsewhere, is not being considered for publication elsewhere and does not duplicate material already published. I confirm all authors consent to publication of this Work and authorise the granting of this licence.

For peer review only

What is the financial burden to patients of accessing surgical care in Sierra Leone? A cross-sectional survey of catastrophic and impoverishing expenditure.

Manraj Phull (1), Caris E Grimes (2, 3), Thaim B Kamara (4), Haja Wurie (5), Andrew JM Leather (2) * and Justine Davies (2, 6, 7, 8) *

*Co-senior authors

Authors

Manraj Phull BSc MBBS MRCS MSc
1) West Hertfordshire Hospitals NHS Trust. Watford General Hospital, Watford, Hertfordshire, UK

Caris E Grimes BSc MBBS MEd FRCS MD
2) King's Centre for Global Health and Health Partnerships, School of Population Health and Environmental Sciences, King's College London, London, SE5 9RJ, UK
3) Medway NHS Foundation Trust, Gillingham, Kent, UK

Thaim B Kamara MBChB, FWACS
4) College of Medicine and Allied Health Sciences, Freetown, Sierra Leone

Haja Wurie
5) College of Medicine and Allied Health Sciences, Freetown, Sierra Leone

Andrew JM Leather MBBS, FRCS, MS
2) King's Centre for Global Health and Health Partnerships, School of Population Health and Environmental Sciences, King's College London, London, SE5 9RJ, UK

Justine Davies (Corresponding Author) MBChB, MD (res), MRCP
2) King's Centre for Global Health and Health Partnerships, School of Population Health and Environmental Sciences, King's College London, London, SE5 9RJ, UK
6) Institute for Applied Health Research, University of Birmingham, Edgbaston, Birmingham B15 2TT UK
7) Medical Research Council/Wits University Rural Public Health and Health Transitions Research Unit, Faculty of Health Sciences, School of Public Health, University of the Witwatersrand, Johannesburg, South Africa
8) Centre for Global Surgery, Department of Public Health, Stellenbosch University, South Africa

Correspondence to Justine Davies: J.Davies.6@bham.ac.uk

For peer review only

Abstract

Objectives: To measure the financial burden associated with accessing surgical care in Sierra Leone.

Design: A cross-sectional survey conducted with patients at the time of discharge from tertiary level care. This captured demographics, yearly household expenditure, direct medical, direct non-medical, and indirect costs for surgical care, and summary household assets. Missing data were imputed.

Setting: The main tertiary level hospital in Freetown, Sierra Leone.

Participants: 335 surgical patients under the care of the hospital surgical team receiving operative or non-operative surgical care on the surgical wards.

Outcome measures: Rates of catastrophic expenditure (CE) (a cost > 10% of annual expenditure), impoverishment (being pushed into, or further into, poverty as a result of surgical care costs), amount of out-of-pocket (OOP) costs, and means used to meet these costs were derived.

Results: Of 335 patients interviewed, 39% were female and 80% were urban dwellers. Median yearly household expenditure was US$3569. Mean OOP costs were US$243, of which a mean of US$24 (10%) was spent pre-hospital. Of costs incurred during the hospital admission, direct medical costs were US$138 (63%) and US$34 (16%) were direct non-medical costs. US$46 (21%) were indirect costs. Catastrophic expenditure affected 18% of those interviewed. Concerning impoverishment, 45% of patients were already below the national poverty line prior to admission, and 9% of those who were not were pushed below the poverty line following payment for surgical care. 84% of patients used household savings to meet OOP costs. Only 2% (6 patients) had health insurance.

Conclusion: Obtaining surgical care has substantial economic impacts on households which pushes them into poverty or further into poverty. The much-needed scaling up of surgical care needs to be accompanied by financial risk protection.

Article Summary
Strengths and Limitations

- Use of exit interviews to provide in depth data on costs of accessing surgical care.
- Thorough and detailed capture of household expenditure.
- Provides reliable estimates of OOP, catastrophic, and impoverishing expenditure as well as sources of financing.
- Data captured in one hospital only, although that is the major surgical care centre for the country.
- Only examines those who accessed care and doesn't allow exploration of costs as a limitation to accessing care.

For peer review only

Introduction

An estimated 33 million individuals globally face financial catastrophe through payment for surgery and anaesthetic care each year. Furthermore 3·7 billion people have been estimated to be at risk of catastrophic expenditure (CE – defined as a total out-of-pocket (OOP) health payment that exceeds a set threshold of the household's annual income or expenditure) due to a lack of financial risk protection (FRP).[1,2] Surgical conditions make up 30% of the global burden of disease and globally an additional 143 million surgical procedures are required annually to meet the current unmet surgical need.[1,3] To ensure universal health coverage, it is therefore essential that FRP is prioritised alongside the scaling up of surgical care. The Lancet Commission on Global Surgery (LCoGS) stated a target of 100% financial protection by 2030 for people accessing surgical care, and FRP indicators for surgery are now included within the World Development Indicators (WDI).[4] Despite this, there is little information on financial implications of accessing surgery in the literature beyond modelled studies,[1,2,5] many of which have been based on few real-world data-points.

Worldwide modelled data on CE and impoverishing expenditure (IE – defined as being pushed into or further into poverty) related to surgical care reveals that those most affected are individuals in low- and middle-income countries (LMICs).[1,2,6] Modelling studies from Sierra Leone, classed as "least developed" by the UN, and with a population of 7 million reflects these findings; between 84.7% and 49.9% of the population in Sierra Leone is estimated to be at risk of CE if they require surgery. Estimated average OOP costs for major surgery in the country were US$117.60, which put 73.3% to 59.2% of the population at risk of impoverishment.[5,7] However, there are no empirical data to validate these estimates. The estimated unmet surgical burden of disease in Sierra Leone is huge, at 92%, as a result of the historical neglect of surgical care both nationally and globally.[8-10] To enable effective planning of surgical services in future, an accurate understanding of the financial implications of accessing surgical services is required.

In Sierra Leone, as in many LMICs, payments for healthcare are upfront, complex, and not immediately apparent from hospital listed service charges. In addition, hospital listed charges – where they exist – may not reflect the total facility-incurred costs that patients pay during their hospitalisation. These include direct medical costs which are charges for the payment of medical care and direct non-medical costs which include items such as transport to the hospital and food. In addition, substantial costs of care may be incurred prior to the hospitalisation episode. For example, there may be direct medical costs at other healthcare facilities visited prior to the definitive admission. Finally, there are indirect costs (e.g. loss of wages whilst receiving care) that patients, and in some cases their caregivers, experience in their illness, which also impact upon ability to access care. Two ways of capturing these costs is the measurement of IE or CE. The two most widely used thresholds for CE are an expense of > 10% of total annual expenditure or > 40% of non-subsistence expenditure (i.e. household expenditure net of subsistence costs, as a means of capturing the ability to pay).[11-14]

This study aimed to measure the financial burden associated with receiving surgical care in Sierra Leone by using an exit survey to determine a) direct medical, direct non-medical, and indirect OOP costs to pay for a surgical care episode b) the rate of impoverishment and catastrophic expenditure, c) the wealth characteristics of the population accessing surgical care relative to that of the general Sierra Leonean population, d) the factors associated with higher costs of hospital care, e) the in-hospital payment mechanism (i.e. where and to whom the OOP payments are being made), and f) how costs of accessing surgical care are met, and the factors associated with meeting costs of care.

Methods

Setting

For peer review only - http://bmjopen.bmj.com/site/about/guidelines.xhtml

This study was done in the main tertiary referral centre in Sierra Leone, located in the central part of greater Freetown, and where the majority of surgical care in the countries' non charitable sector is done. It is a 400-bed hospital with 150 beds dedicated to surgical care. Surgical care is delivered in 5 of the 10 wards, an accident and emergency department with a trauma ward for short stay (< 24hrs) emergency surgical patients, a surgical outpatient unit, an intensive care unit and five operating theatres. The average surgical volume is 80 -100 operations per month.[15]. The surgical department is run by 8 surgical and 2 anaesthetic consultants covering six specialities: general surgery, surgical oncology, urology, paediatrics, trauma and orthopaedics, and ear, nose and throat (ENT) surgery. Obstetric and gynaecological surgical care is delivered at a nearby tertiary referral hospital dedicated to women's health, where all pregnant and lactating women receive free healthcare under the government's free health care initiative and therefore not included in this study.

Participants

Participants were all surgical patients who consented to take part, receiving operative or non-operative surgical care under the care of the hospital surgical team and located on one of the surgical wards. Patients under the care of non-surgical teams; patients under the age of 16 who were without a parent, guardian, or head of the household; and participants unable to consent and/or unwilling to take part in the study were excluded. Participants were recruited consecutively to the study on admission for surgical care from June to August 2018.

Data collection

A structured questionnaire was administered to patients and/or their relatives at the time of formal discharge from surgical care while patients were on the ward. Where patients self-discharged or left against medical advice, where possible they were interviewed when leaving the hospital. Interviews were conducted in a private space and all participants were encouraged to bring a relative, head of the household, or the main breadwinner to allow for expenditure and OOP costs to be captured accurately.

The questionnaire was designed based on tools used in similar studies done in LMIC settings.[16-19] It was co-designed with in-country experts, healthcare professionals, and researchers to ensure that the questions were suitable for the Sierra Leone context. The questionnaire was pilot-tested for ease of comprehension, clarity of definitions, appropriateness of questions, and manageability of the length of the interview in six patients (who were excluded from the analysis). Minor modifications were made to the wording of the questions based on this, but the meaning of the questions was not changed. The questionnaire was designed and written in English and administered by trained Sierra Leonean research assistants (RAs) in either English or a chosen local dialect (most commonly Krio). Data was captured on paper and later transferred to electronic format.

Definition and construction of variables

Data was collected on the particpants' age, gender and address (later used to determine if they were resident in an urban or rural area). The occupation of the main breadwinner was recorded using free text followed by a question on whether this was salaried (i.e. employed) or non-salaried (i.e. self-employed or working in the informal sector). Education was captured as the highest level of education of the main breadwinner. Information on household expenditure was captured by asking 7 questions on regular items purchased in a typical week (food and drink etc.), 11 questions on larger expenditure items typically purchased monthly (toiletries, clothing, etc.) and a further 12 questions on typical yearly spend on big household items such as furniture and livestock (see Appendix 1). Total food expenditure ($foodexp_h$) was summed as a separate variable for the purposes of calculating CE (where food expenditure was used to define subsistence costs). Number of people living in the household (*HHsize)* was also captured, as was the number of days of sickness before presentation, whether care

For peer review only - http://bmjopen.bmj.com/site/about/guidelines.xhtml

had been sought elsewhere prior to presentation at Connaught Hospital, and the mode of transport used.

Data was also collected on the following: whether the patient was an emergency or elective case; whether or not the participant was eligible for free healthcare (for patients under the age of 5 years old, pregnant or lactating mothers, Ebola survivors, destitute and disabled patients); and the primary diagnosis, recorded from review of the patient's admission notes, ward and theatre ledgers (later summarised into 10 categories of surgical conditions: trauma, hernia, abdominal conditions, peripheral vascular disease or diabetic foot disease, urological conditions, breast mass / cancer, burns, ENT / dental disease, thyroid, congenital abnormality, or paediatrics. Treatment was categorised as operative or non-operative following review of the patient's admission notes. Length of hospital stay was also calculated.

Direct medical OOP costs were captured across the entire illness episode including in-hospital costs (from the point of admission to discharge from the tertiary care hospital) and pre-hospital costs (for other medical costs related to the admission episode which occurred prior to the tertiary care admission). In-hospital direct medical costs were the sum of administrative costs (including registration, admission, triage, bed and discharge fees), medications, medical supplies, investigations, blood transfusion, operation cost, and informal payments (defined as any payment that was not part of hospital policy, such as doctors' fees, tips, payments made to porters and to nursing staff for nursing care). If costs were 'formal', we asked whether these costs were paid directly to the hospital bank / cashiers directly or via hospital staff, or to an external facility (such as external pharmacy or laboratory). For pre-hospital care, non-medical direct costs were calculated from transport costs. For the hospital episode, non-medical direct costs were captured as: cost of transport to the hospital or to and from the hospital to get food, medical supplies and investigations from external facilities, and the cost of food and accommodation during the hospital stay. Finally, indirect costs were captured by estimating lost wages during the illness episode.

All costs are presented in Le and $US at the conversion rate of 15th July 2019 (1 Sierra Leonean Leone = 0.00011567 USD).

Total household expenditure ($totalexp_h$) was calculated over the course of 12 months by summing all the variables collected on all regular household items purchased as described above.

Total OOP payments for surgical care (OOP_t): = total direct medical costs + total direct non-medical costs + total indirect costs

Catastrophic expenditure (CE) is most widely defined as either an expense more than 10% of total annual expenditure or an expense of more than 40% of non-subsistence expenditure (i.e. household expenditure net of subsistence [here, food ($foodexp_h$)] costs) . We considered 10% of total household expenditure to be our main outcome of CE, but present results from the 40% of non subsistence expenditure as a sensitivity analysis.

CE was therefore present if: $\frac{OOP_t}{totalexp_h} > 0.1$

In the sensitivity analysis, using the threshold of 40% of non subsistence expenditure, CE was present if $\frac{OOP_t}{totalexp_h - foodexp_h} > 0.4$
:

For peer review only - http://bmjopen.bmj.com/site/about/guidelines.xhtml

Impoverishing Expenditure (IE) is defined as being pushed into or further into poverty. The Sierra Leone national poverty line (spending < $1.25/person /day) threshold was used for the main analysis. In addition, two further thresholds for poverty were used based on World Bank definitions: "poverty" - spending < $3.10/person/day and "extreme poverty" - spending <$1.90/person/day.[4] Presence of poverty before (baseline) and after OOP spending on surgical care were then calculated.

Baseline poverty (BLP_h) at each threshold was determined to be present if total household expenditure ($totalexp_h$) per individual inhabiting each household divided by the number of days in the year was below the poverty threshold chosen. i.e.: $\frac{\left(\frac{totalexp_h}{HHsize}\right)}{365} \leq poverty\ line$

Impoverishment as a result of surgical care was defined as present if the total household expenditure net the total OOP costs for surgical care ($totalexp_{netsurg} = totalexp_h - OOP_t$) per head of household, per day was less than the chosen poverty threshold

i.e.: IE present if $\frac{\left(\frac{totalexp_{netsurg}}{HHsize}\right)}{365} \leq poverty\ line$

Both CE and IE are presented as the number and percentage of participants who experienced CE and or IE.

Summary household asset data was collected using a yes or no response to the ownership of the following assets: Electricity / Light, Mobile phone, Radio, Television, Computer, Refrigerator, Generator, Bicycle, Motorcycle and Car or truck.

Sample size and power calculation

Sample size was calculated using the USCF online calculator[20]. Based on a similar study done in Uganda which estimated CE to be 31%[16] in a free healthcare setting, modelled and World Bank data for Sierra Leone which estimates CE at 84.7% and 49.9% respectively, and from discussion with academics with in-country knowledge, we estimated that CE would be around 60% of patients admitted for surgical care. The sample size required to capture this with a CI of 55-65%, allowing for 10% loss to follow up was 442 patients.

Statistical analysis

Statistical analysis was done using SPSS Version 25 for windows.

Characteristics of the population seeking care are described. Normally distributed data are presented as mean and standard deviation (SD), otherwise median, IQR and range are used. Multiple Imputation Chained Equations were used to compute missing data-points using predictive mean matching for variables with less than 20% missingness and where missingness was identified as not at random. Where imputed variables were used, the pooled mean is shown as standard SPSS output. A complete case analysis was done for variables describing how costs of accessing care were met and the consequences of accessing care.

Wealth characteristics (household asset ownership) of the population accessing surgical care were compared with those in the general population (2015 Census data[21]) using the Chi squared test.

Associations between direct medical in-hospital OOP costs of care and age, sex, type of admission (emergency or elective), operative or non-operative care, type of operative procedure, or length of stay were tested using a generalised linear model using a Tweedie function with a power of 1.9.

Ethical approval

For peer review only - http://bmjopen.bmj.com/site/about/guidelines.xhtml

Ethical approval was granted by the Sierra Leone Ethics and Scientific Review Committee and from the King's College London Research Ethics Committee (ref. LRU-17/18-6455)

All patients gave written consent to participate where possible and witnessed thumbprints and verbal consent where patients were illiterate. Patients were given information about the study at admission and consented between 4-24 hours later after due time was given to consider the study information. Consent was re-confirmed just prior to doing the exit interview.

Results

Of the initial 416 recruited participants, a total of 335 were interviewed (Figure 1). Participant characteristics are presented in table 1. In summary, the mean age of the interviewed patients was 28 (SD 20). 39% were female and 80% lived in an urban area. 29% were formally employed with a further 66.9% being employed but without a regular salary – either self-employed or employed within the informal sector. The level of education of the main breadwinner was secondary school in 38%, college / university in 28% and no formal education in 24%. The median household size was 6 (IQR: 4, range: 4-8) with a mean total yearly household expenditure of US$3569 (see appendix table 2 for imputed and non-imputed data and appendix table 3 for a comparison with expenditure assessed in the Economic and Financial survey in 2014[22]). 67% of participants had sought care for their illness elsewhere prior to presentation at the tertiary referral hospital. 72% arrived by public transport and the majority were classed as emergency admissions (72%). The most common reasons for presentation were trauma, hernia, or other abdominal conditions. 68% underwent operative intervention with the remainder being managed by non-operative measures. Median length of stay was 8 days (IQR: 18, range: 3-21).

Table 1: Participant characteristics

**Demographics of participants**	
Total number of patients interviewed	335
Mean age in years (SD)	28 (20)
Female number (%)	132 (39%)
Urban Residents (%)	269 (80%)
**Type of job (number (%)):**	
Self Employed / Informal Sector	224 (67%)
Employed	97 (29%)
Unemployed / Retired	12 (4%)
Missing /Don't know	2 (1%)
**Level of education of main breadwinner (number (%)):**	
No formal education	79 (24%)
Primary school	25 (8%)
Secondary school	127 (38%)
College / University	94 (28%)
Other / Missing / Don't know	10 (3%)
Median household size (IQR, (range))	6 (4, (4-8))
Total yearly household expenditure (US$)	$ 3,569
Number below national poverty line prior to illness	151 (45%)
**Surgical Care Episode Descriptors**	
Median days of sickness before presentation (IQR, (range))	2 (14, (0-14))
Number that sought care for illness elsewhere prior to presentation at Connaught	225 (67%)

For peer review only - http://bmjopen.bmj.com/site/about/guidelines.xhtml

**Mode of transport used to travel to hospital (number (%)):**<br>Public transport<br>Ambulance<br>Private transport<br>Walked<br>Don't know / Missing	 241 (72%)<br>67 (20%)<br>23 (7%)<br>3 (1%)<br>1 (0%)
Emergency admission (%)	242 (72%)
Eligible for free health care (%)*	70 (21%)
**Primary diagnosis by surgical condition (number (%))**<br>Trauma<br>Hernia<br>Abdominal conditions<br>Peripheral vascular disease or diabetic foot disease<br>Urological conditions<br>Breast mass / cancer<br>Burns<br>ENT / dental disease<br>Goitre<br>Congenital abnormality (paediatrics)<br>Missing / don't know	 114 (34%)<br>58 (17%)<br>56 (17%)<br>27 (8%)<br>23 (7%)<br>16 (5%)<br>15 (5%)<br>13 (4%)<br>7 (2%)<br>3 (1%)<br>3 (1%)
**Treatment (number (%)):**<br>Operative<br>Non-operative	 226 (68%)<br>109 (33%)
Median length of hospital stay (LOS) in days (IQR, (range))	8 (18, (3-21))

* Eligible for free health care indicates those that fall under the government Free Health Care Initiative (FHCI); a health financing policy introduced in 2010 aimed to significantly improve maternal and child health through the provision of free healthcare services for all children under 5, pregnant and lactating women. This was later extended to include Ebola survivors.

The total mean cost for the surgical care episode was US$243 of which US$24 (10%) accounted for pre-hospital direct costs (medical costs were US$21 and non-medical were US$3). Of the in-hospital direct costs (mean US$172), a mean of US$138 (63%) was due to direct medical costs and US$34 (16%) for direct non-medical costs. Indirect costs, such as lost wages, totalled US$46. (Table 2 and appendix table 4).

Table 2: Out-of-pocket costs.

Costs	Imputed mean cost ($US (% of subtotal))
**Prehospital costs**	
**Direct pre-hospital medical OOP costs (total)**	**21 (88% of 24)**
- Consultation	2 (10% of 21)
- Medications	12 (57% of 21)
- Medical supplies	2 (10% of 21)
- Investigations	4 (19% of 21)
- Other miscellaneous	2 (10% of 21)
**Direct (pre-hospital) non-medical OOP costs (total)**	**3 (13% of 24)**
- Transport	3 (100% of 3)
Total pre-hospital costs	***24 (10% of 243)***

For peer review only - http://bmjopen.bmj.com/site/about/guidelines.xhtml
For peer review only

In hospital costs	
**Direct medical OOP costs (total)**	**138 (63% of 219)**
- Administrative	20 (14% of 138)
- Medications	26 (19% of 138)
- Medical supplies	14 (10% of 138)
- Investigations	15 (11% of 138)
- Blood transfusion	9 (7% of 138)
- Total operation costs	49 (36% of 138)
- Unofficial costs	6 (4% of 138)
- Other / miscellaneous	1 (1% of 138)
**Direct non-medical costs (total)**	**34 (16% of 219)**
- Transport to hospital	7 (21% of 34)
- Food	20 (59% of 34)
- Accommodation	0 (0% of 34)
- Other*	7 (21% of 34)
**Indirect costs**	
- Lost wages	46 (100% of 46)
**TOTAL OOP COSTS**	**243**

*other relates to travel and other associated costs incurred as a result for needed investigations from and or medication / supplies from an external facility. SPSS calculates only the mean using imputed variables, hence no standard deviation is displayed.

For peer review only

Of the in-hospital direct medical costs, 48% were given to hospital staff (it was not clear whether the hospital staff later transferred these funds to the hospital bank or not), 33% were made directly to the hospital bank / cashiers and 17% to an external facility such as external pharmacy or diagnostic centre (Appendix table 5).

A variety of means were used to meet costs and participants were allowed to mention more than one means of covering costs (Table 3). Most (84% of patients) used their savings to meet some or all of the costs, with family contributions, borrowing money and charitable donations forming the 2nd, 3rd and 4th most frequently used means of meeting OOP payments, respectively. Only 2% (6 patients) had some form of health insurance. Wider implications included loss of wages in 37% and loss of job in 6.0%.

Table 3: How costs are met and the wider implications of seeking and undergoing surgical care (n is the number of cases with data on each variable)

How costs were met (total number responding to question)	Number (%) that used this as a means of meet OOP costs
Used **Savings** (n=326)	**273 (84%)**
Arranged **family contributions** (n=331)	**128 (39%)**
**Borrowed** money (n=331)	**102 (31%)**
Received **charity** money (n = 331)	**83 (25%)**
**Sold possessions** (n=329)	**17 (5%)**
**Other** (n=331)	**14 (4%)**
**Pawned** possessions (n=332)	**8 (2%)**
Have **Health insurance** (n=335)	**6 (2%)**
**Wider implications**	**Number (%) that experienced the wider implications of meeting OOP costs**
**Loss of wages** (n = 328)	**121 (37%)**
**Lost their job** / changed their role at work / home (n = 331)	**20 (6.0%)**
**Disruption to education** (n = 333)	**12 (4%)**

Catastrophic expenditure, when defined as OOP costs of more than 10% of all household expenditure affected 18% of those interviewed. In the sensitivity analysis using the threshold of more than 40% of non-subsistence expenditure, catastrophic expenditure affe,cted 10% of those interviewed..

Prior to the surgical care episode, 45% of people interviewed were below the national poverty line, 90% were below the World Bank Poverty Level, and 70% below the World Bank Extreme Poverty level. Following payment for surgical care, 50% were pushed below or further below the national poverty line. Corresponding figures were 91% and 73%, for the World Bank thresholds of poverty and extreme poverty, respectively.

Analysis of the possession of household assets demonstrated that those interviewed were more likely to have electricity, a mobile phone, radio, television, refrigerator, bicycle, motorcycle or car than those of the general population in Sierra Leone (2015 Census data, all p=<0.001) or of the urban population in the Western Area (2015 Census data, all p=<0.05) (Table 4).

Table 4: Ownership of household assets in comparison to 2015 census data

Household assets	Surgical cohort	2015 Census data Whole country data

For peer review only - http://bmjopen.bmj.com/site/about/guidelines.xhtml

	Number (%) of households that own the asset	
**Electricity**	227 (67.8%)	17.8%
**Mobile phone**	326 (97.3%)	62.94%
**Radio**	280 (83.6%)	58.03%
**Television**	212 (63.3%)	19.76%
**Refrigerator**	119 (35.5%)	8.22%
**Bicycle**	38 (11.3%)	6.43%
**Motorcycle**	8 (14.3%)	7.62%
**Car**	50 (14.9%)	3.65%

For peer review only

Regression analysis demonstrated that the factors associated with greater costs were older age, longer length of hospital stay and undergoing a general surgical or urological procedure (Appendix table 6).

Discussion

In this study, we found that accessing and receiving tertiary level surgical care in Sierra Leone requires large up-front OOP payments which have a substantial impact on individual and households' economic situations. These equate to a catastrophic expense in nearly a fifth of households and are impoverishing half of the households that receive care. We found poverty, as assessed by household expenditure, was high, indicating a limited financial buffer to accommodate costs of care. This is despite most people who access surgical care owning a higher level of assets than the general population.

The majority of the OOP payments were incurred in-hospital and as a result of direct medical costs. Payment for the operation itself and medications, medical supplies, and investigations (including laboratory tests) were the biggest contribution to these costs. A small percentage of costs were categorised as unofficial, such as for "nursing care" and "tips", although these were given by a majority of people who received care. In addition, almost half of these were being paid through unofficial payment channels and made directly to staff. We do not know whether these payments were later transferred to the hospital bank, however, these informal routes are common and indicate poor financial governance which urgently needs to be addressed.

The majority of payments were met using savings, followed by raising money from family or borrowing money. In addition, a large number of participants lost wages during the sickness episode and a proportion lost their jobs. In a country where informal work predominates and earnings can be unpredictable, this may impact on household financial security and influence future health seeking behaviour, both of the individuals affected and their immediate family and communities.

The majority of patients accessing surgical care were young males; whether this male predominance is a true reflection of surgical disease burden, beyond obstetrics and gynaecological care, in Sierra Leone or reveals a hidden gender bias in care seeking behaviour is beyond the remit of this study. Nevertheless, males who sought care in our study are traditionally the main breadwinners and the most economically active population group in Sierra Leone. This loss of wages and livelihood could have implications on the wider socio-economic determinants of health and the well-being of the household. The additional burden to the patients and their households as a result of the indirect costs supports the macroeconomic argument for investing in surgical care put forward by Grimes et al, who demonstrated the opportunity to avert 36,487 DALYs by investing in surgical care at hospital level in Sierra Leone. [23,24]

Some specialties, such as general surgery and urology incurred much higher overall costs for the surgical episode and this may be because operative intervention (with blood transfusion and a longer length of stay) is usually required. This contrasts for example, to trauma care that was often managed non-operatively. Such non-operative treatment for trauma may be partly as a result of local surgical practice, often driven by lack of resources such as the unavailability of internal fixation wires and orthopaedic implants, and partly because some common orthopaedic problems are managed non-operatively. In addition, we found that age and length of hospital stay were associated with significantly higher costs. This may be due to the fact that those under the age of 5 years were eligible for free health care in Sierra Leone and that a longer stay in hospital was associated with higher direct non-medical and indirect costs such as payment for food and lost wages.

For peer review only - http://bmjopen.bmj.com/site/about/guidelines.xhtml

There are a limited number of studies to draw a direct comparison with as only a few used a similar methodology (direct interview) as opposed to modelled data or the use of caesarean section costs as a proxy measure to extrapolate costs, CE and impoverishment.[2,16,25-30] There are even fewer studies that report on the financial implications of all or most types of surgical care. The majority report on single surgical subspecialties such as obstetric care, paediatric surgery or trauma care. Nevertheless, there have been three recent studies from Uganda reporting CE rates of 31% and 55% and IE of 47%.[16,31,32] A study in Malawi interviewing patients undergoing hernia operations reported CE rates as high as 90% using a threshold of 10% of yearly income.[28] Various studies looking at injury and trauma care costs in Vietnam, India and Nigeria have reported CE rates of 60%, 30% and 86% respectively[25] and a study in Morocco looking at obstetric surgical care alone estimated CE rates of 88%[33] while an emergency obstetric care study in Indonesia estimated CE at 68%.[34] The inter-country variability makes it difficult to draw comparative conclusions. This highlights the need for a standardised way of assessing and measuring the financial implications of surgical care, to allow accurate collection and reporting of these global surgery metrics on financial risk protection.

In keeping with other studies, we noted lower rates of CE and IE in comparison to the modelled and extrapolated estimates for Sierra Leone. This is probably because the modelled studies are based on the whole population that may require surgery and not on those that have successfully accessed surgical care. The lower rates of IE and CE seen may therefore be explained by a lack of access by the poorest. This is supported by data from Sierra Leone that estimates that up to 25% of deaths in 2011 could have been averted through access to safe, timely and affordable surgical care and that Sierra Leone has an unmet surgical burden of disease of 92% [10], with approximately 70% of Sierra Leoneans stating that the financial burden of OOP payments for healthcare was the biggest barrier to accessing care.[35,36] In addition, we found that those accessing tertiary level surgical care came from predominantly urban areas of Sierra Leone and when compared to the wider Sierra Leone population, had significantly higher asset ownership. It may be therefore that the poorest and those at the highest risk of financial catastrophe are not accessing care when needed. This may also reflect other known barriers to seeking surgical care in LMICs that are often complex and multifactorial such as cultural beliefs, attitudes and fears towards surgical care and structural barriers such as geographical access, transport links and referral systems.[37]

Limitations

There are several limitations to this study. Firstly, it was dependant on recall and self-reported estimates of OOP costs and household expenditure. Although the questionnaire and methodology are a well-established way of obtaining this information in a low-resource setting where informal work predominates and payments are not often receipted. To increase accuracy of data collected, household expenditure questions were broken down to weekly, monthly and yearly costs, a chronological approach was used to the OOP cost questions that helped map out the patients journey for them, participants were encouraged to bring an appropriate family member to the interview, and in-country consensus gained and the questionnaire piloted prior to use.

Secondly, given that patients were often interviewed on the wards and potentially within hearing range of nurses, data on informal payment methods and informal costs, may not have been fully reported. If this were the case, we would have expected to see more missing data for payments made directly to staff in comparison to those made to the banks, however we did not observe this. This suggests that participants did not appear to be deterred from sharing this information.

Thirdly, the study only measures costs incurred during the illness episode up until discharge. We have therefore likely substantially underestimated the total costs of seeking surgical care.

For peer review only

In addition, in Sierra Leone tertiary level obstetric care is provided at a different hospital and offered free of charge. Therefore, costs of accessing this were not included in this study. Further work needs to be done to see if those receiving free maternal healthcare incur any OOP costs and if informal payments such as tips paid to staff are as prevalent in the obstetric care hospital.

Finally, the desired sample size was not achieved as not all surgical patients admitted were interviewed. This was mostly due to many being discharged out of hours, at the weekend or after a short admission on the acute trauma ward, before the study team could consent or interview them. This may indicate that these patient had minor pathology, a shorter stay and lower OOP costs. Inclusion of these cases may have lowered the mean OOP costs, CE and IE rates but would poorly represent the financial barriers and wider implications of accessing surgical care for those that may have absconded or self-discharged due the cost of care. Nevertheless, although sample size was not obtained, the 95% confidence interval for a catastrophic expenditure rate of 18% was 14-22% which gives the study an overall power of 90%.

Conclusion

This is the first empirical study from Sierra Leone that quantifies the financial burden of accessing and receiving surgical care. It adds insight into the global and national Sierra Leone modelled estimates of the likelihood of catastrophic and impoverishing expenditure if surgery is required and joins the small but growing body of other empirical studies reporting on the OOP costs and wider financial implications of surgical care. In addition, it highlights the need to prioritise financial risk protection within healthcare and surgery if universal health coverage is to be achieved.

Figure Legends

Figure 1: Study Recruitment Process Diagram

Author Contribution: JD, AL, TBK and HW conceptualised the study. MP, JD, and AL developed the protocol and survey tools; MP, JD, and CG analysed the data; all authors contributed to the interpretation of the results and write up of the manuscript; All authors approved the manuscript for publication.

Competing interests: None declared.

Funding: This research was partly funded by the National Institute of Health Research (NIHR) Global Health Research Unit on Health System Strengthening in Sub-Saharan Africa, King's College London (GHRU 16/136/54) using UK aid from the UK Government to support global health research. The views expressed in this publication are those of the author(s) and not necessarily those of the NIHR or the Department of Health and Social Care.

Data Sharing: Further data is available on reasonable request from the corresponding author.

Patient and Public Involvement / Cohort Description: The Lancet Commission on Global Surgery has shown that out of pocket expenditure limits patients ability to access surgical care when needed. Accessing care for a surgically treatable disease to reduce mortality or morbidity is a priority for patients. The methodology employed was standard for assessing out of pocket costs, wealth, and healthcare expenditure. Patients were not involved in designing these methods, however, they were involved in testing and refining them to ensure appropriateness to a local setting. No patients were involved in assisiting with the recruitment to and conduct of the study.

For peer review only - http://bmjopen.bmj.com/site/about/guidelines.xhtml

As part of the ethics board approval, we did not collect contact details of the patients involved in this study and hence cannot disseminate the results to them. However, the results are being shared widely amongst policy workers, community leaders, and clinicians in Sierra Leone. The patient advocacy movement in Sierra Leone, like in many low-income countries, is nascent, hence there are no patient groups with which to share results. We hope that our work will galvanise greater advocacy and enable sharing more widely.

Acknowledgements: We thank the healthcare workers and patients who were involved in refining the data collection tool to ensure its applicability to a local setting.

References

1. Meara JG, Leather AJ, Hagander L, et al. Global Surgery 2030: evidence and solutions for achieving health, welfare, and economic development. *Lancet* 2015; **386**(9993): 569-624.
2. Shrime MG, Dare AJ, Alkire BC, O'Neill K, Meara JG. Catastrophic expenditure to pay for surgery worldwide: a modelling study. *Lancet Glob Health* 2015; **3 Suppl 2**: S38-44.
3. Shrime MG, Bickler SW, Alkire BC, Mock C. Global burden of surgical disease: an estimation from the provider perspective. *Lancet Glob Health* 2015; **3 Suppl 2**: S8-9.
4. World Bank Development Indicators 2016. http://data.worldbank.org/data-catalog/world-development-indicators (accessed 10th August 2018.
5. Shrime MG, Dare A, Alkire BC, Meara JG. A global country-level comparison of the financial burden of surgery. *Br J Surg* 2016; **103**(11): 1453-61.
6. WHO. World Health Organisation: Health statistics and information systems. Global Reference List of 100 Core Health Indicators. . 2015. http://www.who.int/healthinfo/indicators/2015/en/
7. World Bank: Risk of catastrophic expenditure for surgical care. Sierra Leone https://data.worldbank.org/indicator/SH.SGR.CRSK.ZS?locations=SL.
8. Farmer PE, Kim JY. Surgery and global health: a view from beyond the OR. *World J Surg* 2008; **32**(4): 533-6.
9. Bolkan HA, Von Schreeb J, Samai MM, et al. Met and unmet needs for surgery in Sierra Leone: A comprehensive, retrospective, countrywide survey from all health care facilities performing operations in 2012. *Surgery* 2015; **157**(6): 992-1001.
10. Groen RS, Samai M, Stewart KA, et al. Untreated surgical conditions in Sierra Leone: a cluster randomised, cross-sectional, countrywide survey. *Lancet* 2012; **380**(9847): 1082-7.
11. Hsu J, Flores G, Evans D, Mills A, Hanson K. Measuring financial protection against catastrophic health expenditures: methodological challenges for global monitoring. *Int J Equity Health* 2018; **17**(1): 69.
12. Xu K, Evans DB, Carrin G, Aguilar-Rivera AM, Musgrove P, Evans T. Protecting households from catastrophic health spending. *Health Aff (Millwood)* 2007; **26**(4): 972-83.
13. Hoang HT, Pham TL, Vo TT, Nguyen PK, Doran CM, Hill PS. The costs of traumatic brain injury due to motorcycle accidents in Hanoi, Vietnam. *Cost Eff Resour Alloc* 2008; **6**: 17.
14. Nguyen H, Ivers R, Jan S, Martiniuk A, Pham C. Catastrophic household costs due to injury in Vietnam. *Injury* 2013; **44**(5): 684-90.
15. National Surgical, Obstetric and Anaesthesia Strategic Plan. Sierra Leone Ministry of Health and Sanitation.
16. Anderson GA, Ilcisin L, Kayima P, et al. Out-of-pocket payment for surgery in Uganda: The rate of impoverishing and catastrophic expenditure at a government hospital. *PLoS One* 2017; **12**(10): e0187293.
17. World Health Organisation. Health statistics and information systems. Data collection tools - World Health Survey. . http://www.who.int/healthinfo/survey/en/ (accessed 19th April 2018).
18. (SAGE). WHOHsaisDct-WSoGAaAH. http://www.who.int/healthinfo/sage/en/

For peer review only - http://bmjopen.bmj.com/site/about/guidelines.xhtml

19. Grimes CE, Billingsley ML, Dare AJ, et al. The demographics of patients affected by surgical disease in district hospitals in two sub-Saharan African countries: a retrospective descriptive analysis. *Springerplus* 2015; **4**: 750.
20. USCF. Sample Size Calculators for Designing Clinical Research https://sample-size.net (accessed 6th October 2020.
21. 2015 Population and House Census: Summary of Final Results. . https://www.statistics.sl/images/StatisticsSL/Documents/final-results_-2015_population_and_housing_census.pdf. Accessed 21st August 2019. .
22. Economic and Financial Survey 2014. . https://www.statistics.sl/images/StatisticsSL/Documents/Publications/2014/econmic_and_financial_survey_2014_2.pdf
(accessed 20th June 2019)
.
23. Grimes CE, Quaife M, Kamara TB, Lavy CBD, Leather AJM, Bolkan HA. Macroeconomic costs of the unmet burden of surgical disease in Sierra Leone: a retrospective economic analysis. *BMJ Open* 2018; **8**(3): e017824.
24. Grimes CE, Law R, Dare A, et al. Cost-Effectiveness of Two Government District Hospitals in Sub-Saharan Africa. *World J Surg* 2017; **41**(9): 2187-92.
25. Nguyen H, Ivers R, Jan S, Pham C. Cost of surgery and catastrophic expenditure in people admitted to hospital for injuries: estimates from a cohort study in Vietnam. *Lancet* 2015; **385 Suppl 2**: S50.
26. Prinja S, Bahuguna P, Gupta R, Sharma A, Rana SK, Kumar R. Coverage and Financial Risk Protection for Institutional Delivery: How Universal Is Provision of Maternal Health Care in India? *PLoS One* 2015; **10**(9): e0137315.
27. Honda A, Randaoharison PG, Matsui M. Affordability of emergency obstetric and neonatal care at public hospitals in Madagascar. *Reprod Health Matters* 2011; **19**(37): 10-20.
28. Bijlmakers L, Wientjes M, Mwapasa G, et al. Out-of-pocket payments and catastrophic household expenditure to access essential surgery in Malawi - A cross-sectional patient survey. *Ann Med Surg (Lond)* 2019; **43**: 85-90.
29. Banu T, Chowdhury TK, Aziz TT, et al. Cost Incurred by the Family for Surgery in Their Children: A Bangladesh Perspective. *World J Surg* 2018; **42**(12): 3841-8.
30. Urua U, Osungbade K, Obembe T, Adeniji F. A cost analysis of road traffic injuries in a tertiary hospital in south-west Nigeria. *Int J Inj Contr Saf Promot* 2017; **24**(4): 510-8.
31. MacKinnon N, St-Louis E, Yousef Y, Situma M, Poenaru D. Out-of-Pocket and Catastrophic Expenses Incurred by Seeking Pediatric and Adult Surgical Care at a Public, Tertiary Care Centre in Uganda. *World J Surg* 2018; **42**(11): 3520-7.
32. Yap A, Cheung M, Kakembo N, et al. From Procedure to Poverty: Out-of-Pocket and Catastrophic Expenditure for Pediatric Surgery in Uganda. *J Surg Res* 2018; **232**: 484-91.
33. Boukhalfa C, Abouchadi S, Cunden N, Witter S. The free delivery and caesarean policy in Morocco: how much do households still pay? *Trop Med Int Health* 2016; **21**(2): 245-52.
34. Quayyum Z, Nadjib M, Ensor T, Sucahya PK. Expenditure on obstetric care and the protective effect of insurance on the poor: lessons from two Indonesian districts. *Health Policy Plan* 2010; **25**(3): 237-47.
35. Ponsar F, Tayler-Smith K, Philips M, et al. No cash, no care: how user fees endanger health--lessons learnt regarding financial barriers to healthcare services in Burundi, Sierra Leone, Democratic Republic of Congo, Chad, Haiti and Mali. *Int Health* 2011; **3**(2): 91-100.
36. Xu K, Evans DB, Kawabata K, Zeramdini R, Klavus J, Murray CJ. Household catastrophic health expenditure: a multicountry analysis. *Lancet* 2003; **362**(9378): 111-7.
37. Grimes CE, Bowman KG, Dodgion CM, Lavy CB. Systematic review of barriers to surgical care in low-income and middle-income countries. *World J Surg* 2011; **35**(5): 941-50.

For peer review only - http://bmjopen.bmj.com/site/about/guidelines.xhtml

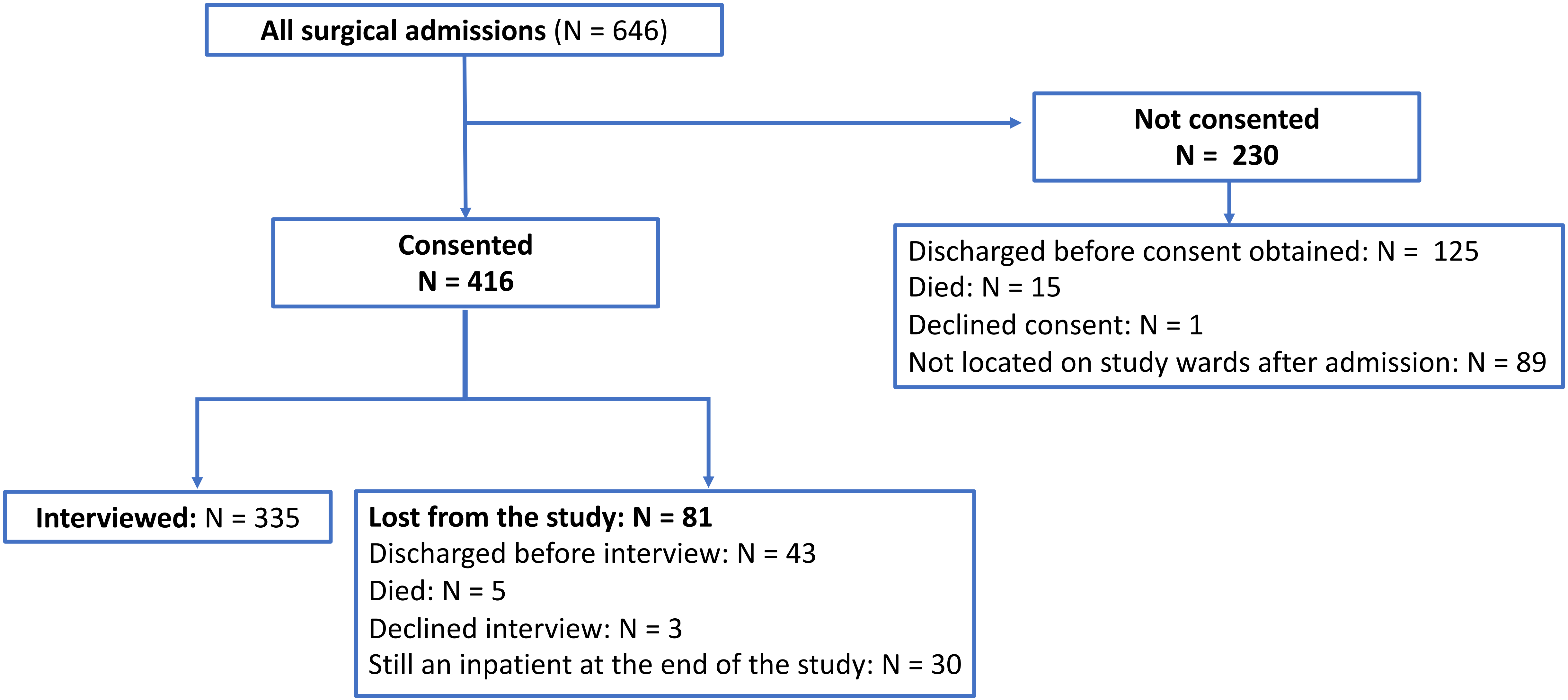

Figure 1: Study recruitment process diagram

Appendix

Additional information on recruitment and training of research assistants

Research Assistants (RAs) were recruited through a competitive process and trained to administer the questionnaire. Training for all RAs was standardised and formally ran over 2 days. This involved; a formal presentation introducing the study, a review of all study processes and associated documents, a role play interview between the RAs using the questionnaire, a walk through the hospital to ensure the RAs gained an insight in to the surgical patients' journey and points at which OOP payments may be made or cost incurred and a review of clinical notes, ward admission books and theatre log books to ensure that all demographic and diagnostic information was accurately captured.

Appendix 1: Study questionnaire

Section 1: Demographics and admission questions

These questions are to be answered in conjunction with the patients notes and screening/recruitment sheet

Code	Question	Response
A1	Participant **unique ID**:	
A2	**Discharge** details	Care episode complete and discharged by Dr ☐<br>Transfer to another healthcare facility ☐<br>Self-discharging ☐ If ticked state reason_________________
A3	Where was the patient **admitted from**?	Connaught<br>Direct to trauma ward / A&E / OPD ☐<br>Referral ☐ specify ________________<br>SOP (specialist outpatients) ☐<br>Through direct contact ☐<br>Transfer from the medical ward ☐<br>Other ☐ specify ______________
A4	Where is the patient being **discharged from**?	Connaught<br>Trauma ward / A&E / OPD ☐<br>Surgical ward ☐<br>Annexe ☐<br>Other ☐ specify __________
A5	**Age** of patient	________________ / Don't know ☐ / Adult (AD) ☐
A6	**Sex**	Male ☐ Female ☐
A7	Usual **residential location** (address including district and from that state if urban or rural)	Address: _____________________________________<br>Urban ☐ Rural ☐
A8	Does the patient meet any exemption criteria for **free treatment**?	Yes ☐ No ☐<br>If yes, state which criteria (tick as applicable)<br>Aged under 5 years ☐<br>Pregnant ☐<br>Lactating ☐<br>Ebola survivor ☐<br>Destitute ☐<br>Disabled ☐
A9	**Type of admission**	Emergency (e.g trauma ward) ☐ Elective ☐
A10	**Primary diagnosis**	____
A11	What **treatment** did the patient receive? *(Note: Procedures are often done in the Trauma ward (minors procedure room) e.g skin traction/POP/suturing)*	**Operation/ procedure** ☐ (specify and go to A14)<br>Specify ____<br>**Non-operative** surgical care ☐ (go to A13)
A12	If patient received **non-operative care** state reason	Clinically appropriate / not recommended by Dr ☐<br>Patient chose not to have an operation / procedure ☐<br>Financial (unable to pay) ☐<br>Other ☐ (please give a brief statement)

For peer review only - http://bmjopen.bmj.com/site/about/guidelines.xhtml

		____
A13	**Length of hospital stay** (admission and discharge date)	**Admission date:** _ _/_ _/_ _ _ _**Discharge date:**_ _/_ _/_ _ _ _

Section 2a: Household structure and typical household income and expenditure
All questions are to be answered by the patient, parent, guardian or household member or head. ***Encourage patient to invite household head / member / breadwinner or the person who deals with household expenditure and or has made the payments for the care received - to help answer the questions.***

"Firstly are a few questions to understand the structure of your household (the people that eat food from the same pot and take instructions from the same head (excluding lodgers / individuals that pay to live in your house) and the average household income"

Code	Question	Response
B1	**Who** is being interviewed? Tick all that are applicable<br><br>If questions are being answered by someone other than the patient (e.g. household member), what is their relationship to the patient?	Patient ☐<br>Parent / Guardian ☐<br>Household head ☐<br>Other ☐ (State relationship to patient)<br>______________________________
B2	What is the **size of your household**, including yourself how many people normally eat food from the same pot and take instructions from the same head (exclude lodgers / individuals that pay to live in your house)	____________________
B3	Does **anyone in your household (household head/members) generate income**?	Yes ☐ (go to B4) No ☐ (go to B7)
B4	**How many people** in your household **generate income** that is used to support the household?	____________________<br>Don't know ☐
B5	What is the **occupation** of the person **who contributes the most** to your household expenses?	____________________<br>Salaried ☐ Non-salaried ☐<br>Don't know ☐
B6	**How much income does your household generate** (in total) to support the household in a typical month?	__________Leones Don't know ☐
B7	What is the **highest level of education of the main breadwinner (i.e the individual identified in B5)?**	No formal education ☐<br>Primary ☐<br>Secondary ☐<br>College ☐<br>University ☐<br>Other ☐ specify ______________
B8	Does your household **have any of the following**?	Electricity / Light Yes ☐ No ☐<br>Mobile phone Yes ☐ No ☐<br>Radio Yes ☐ No ☐<br>Television (TV) Yes ☐ No ☐<br>Computer Yes ☐ No ☐<br>Refrigerator Yes ☐ No ☐<br>Generator Yes ☐ No ☐<br>Bicycle Yes ☐ No ☐<br>Honda/ Motorcycle Yes ☐ No ☐<br>Car or truck Yes ☐ No ☐
B9	What material is used for the **roof of your house**?	Natural roofing (thatch) ☐<br>Basic roofing (tarpoline, metal, zinc) ☐<br>Finished roof (concrete / tiled) ☐<br>Other ☐ specify ______________
B10	What material is used for the **floor of your house**?	Natural floor (mud/earth/wattle) ☐<br>Basic floor (wood/cement) ☐<br>Finished floor (concrete/tile/carpet) ☐<br>Other ☐ specify ______________
B11	What material is used for the **walls of your house**?	Natural walls (mud/earth/wattle) ☐<br>Basic walls (stone/mud bricks/zinc) ☐<br>Finished walls (concrete) ☐<br>Other ☐ specify ______________

Section 2b: Typical household expenditure
"I would now like to ask you some questions about your household expenses starting with how much your household spends on food and consumption in a typical week"

For peer review only - http://bmjopen.bmj.com/site/about/guidelines.xhtml

Code	Question	Response
C1	Does the month of **Ramadhan** effect your household spending/expenses?	Yes ☐ No ☐ **If yes, say: please answer all the questions on expenses based on a week / month outside of the Ramadhan period**
C2	How much does your household spend on **food (including chop money)**?	________________Leones Don't know ☐
C3	How much does your household spend **drinks** (non-alcoholic drinks such as water, tea, coffee, milk and soft drinks?	________________Leones Don't know ☐
C4	**Tobacco and alcoholic beverages** (including beer, wine, spirits, poyo) – had at home / outside	________________Leones Don't know ☐
C5	Food eaten outside the dwelling (for example, at vendors, **cookery**, kiosks or restaurants)	________________Leones Don't know ☐
C6	**Other food** items (e.g kola nut, food not included in chop money)	________________Leones Don't know ☐
C7	Communication fees, including megabites (internet), mobile phone (**credit /top up)** and others?	________________Leones Don't know ☐
C8	**Transportation** (to work place, market, school etc)? (For example, petrol, taxis, motorbike taxis)	________________Leones Don't know ☐

"I would now like to ask you about other expenses your household might have had in the last month or a typical month"

C9	**Utilities, such as water, light, electricity (NPA), waste disposal,** etc.?	________________Leones Don't know ☐
C10	**Fuel (e.g cooking / generator** - gas, coal, kerosene, firewood, petrol, diesel, etc.)?	________________Leones Don't know ☐
C11	**Personal toiletries** and personal care (e.g.soap, toothpaste, toothbrush, toilet roll, cosmetics, beauty salon, getting hair done etc.)?	________________Leones Don't know ☐
C12	**Clothing** and bedding?	________________Leones Don't know ☐
C13	**Loan repayments** (e.g on your house) / other **debt** or **microcredit** for business or other purposes?	________________Leones Don't know ☐
C14	**Entertainment**, including; cinemas/video centres to watch football matches, games, stadium for shows, soccer matches, or hangouts / chilling?	________________Leones Don't know ☐
C15	Payments for **household help**/servants, including cook, maid, driver, security, gardener, etc.?	________________Leones Don't know ☐
C16	How much does your household pay in **remittance** to family living away from home (**money, airtel/Africell money**, food etc)?	________________Leones Don't know ☐
C17	Excluding this hospital stay, how much money does your household usually spend on health care related to **"white man medicine",** including medicines, fees for doctors' appointments and hospital visits?	________________Leones Don't know ☐
C18	Excluding this hospital stay, how much money does your household usually spend on health care related to **"country/black man medicine**"?	________________Leones Don't know ☐
C19	Do you have any **other monthly expenditures**?	Yes ☐ Specify ____________________________ No ☐ (go to C21)
C20	**How much do these other expenditures** amount to?	________________Leones Don't know ☐
"I know these questions may be difficult to answer but try to give me the best estimate of expenses. I would now like you to focus on household expenses **over the last 12 months**. These are expenses that may be more periodic or "big purchases"		
C21	**Rent** (per year)	________________Leones Don't know ☐
C22	**Education fees and school supplies** (tuition, course fees, books)?	________________Leones Don't know ☐
C23	**Electrical goods** (mobile phones, televisions, DVD, radio, refrigerators,)?	________________Leones Don't know ☐
C24	**Big purchases** such as;<br>**Furniture** (tables, chairs, beds, sleeping mats)<br>**Vehicles** (trucks, cars, motorcycles, keke, bicycles) and **upkeep/repairs**?<br>**Tools** (brooms, nails, hammer, paint, shovel, gardening equipment etc)	 ________________Leones Don't know ☐<br>________________Leones Don't know ☐<br>________________Leones Don't know ☐
C25	**Hosting rituals or ceremonies** (funerals, birthdays, wedding, naming ceremony, pray de close, Christmas, the Haj)?	________________Leones Don't know ☐
C26	**Gifts for ceremonies** (funerals, birthdays, wedding, naming ceremony, pray de close, Christmas) if invited?	________________Leones Don't know ☐
C27	**Donations or contributions** to religious organisations (e.g. church/mosque)	________________Leones Don't know ☐
C28	**Deposit / upfront payments for property or land** (excluding monthly loans)?	________________Leones Don't know ☐
C29	Cleaning services or repair service (**house maintenance**)?	________________Leones Don't know ☐
C30	**Livestock** (chicken, goat, sheep etc)?	________________Leones Don't know ☐

For peer review only - http://bmjopen.bmj.com/site/about/guidelines.xhtml

C31	**Taxes** (city rate for home owner, vehicle tax, city council tax, NASSIT (national social security insurance trust)?	________________Leones Don't know ☐
C32	**Health insurance** premiums (including, community health insurance schemes) or pre-paid health plans?	________________Leones Don't know ☐

Section 3: Out-of-pocket payments for care sought prior to admission (from the day they fell ill with this illness)
"I would now like to ask you about all the health costs to your household before coming to hospital from when you fell sick with this illness / problem"

Code	Question	Response
D1	**When did you fall sick with the illness** that meant you had to be admitted to hospital?	______days before admission<br>______weeks before admission<br>______months before admission<br>Don't know ☐ (go to section 3 (C1)
"I would now like you to think about that period of time; **from falling sick to getting to the point of decision to come to Connaught / PCMH.** The following questions will be related to that time. I know that it may be difficult to think that far back but please give the best estimate you can of the costs"		
D2	Did you seek care for this illness elsewhere **before coming to this hospital**?	Yes ☐ (go to D3) No ☐ (go to section 4 (E1))
D3	**Where did you go** to get care for your illness before coming to this hospital? Tick all that are applicable<br><br>For each type of care provider ticked from the list below ask about the **number of visits** and a **cost break down**. If the patient cannot give a breakdown of costs ask for an **estimated total**<br>***Guidance notes;***<br>***Consultation -*** *fees to the provider ("doctors' fees"), fees excluding investigations and tests*<br>***Medications***<br>***Medical supplies*** *- Consumables, gloves, bandages, dressings etc*<br>***Investigations*** *– Includes labs, scans, x-rays and other imaging*<br>***Transportation*** *- transport for getting to and from the facilities, subsequent investigations/ getting treatments / medicines*	

Private clinic ☐ no. of visits ___
How much was spent on;
Consultation _________________Leones Don't know ☐
Medications _________________Leones Don't know ☐
Medical supplies ______________Leones Don't know ☐
Investigations ________________Leones Don't know ☐
Transportation _______________Leones Don't know ☐
Other ______________________Leones Don't know ☐
OR Estimated total ____________Leones Don't know ☐

Charity or religious clinic ☐ no. of visits ___
How much was spent on;
Consultation _________________Leones Don't know ☐
Medications _________________Leones Don't know ☐
Medical supplies ______________Leones Don't know ☐
Investigations ________________Leones Don't know ☐
Transportation _______________Leones Don't know ☐
Other ______________________Leones Don't know ☐
OR Estimated total ____________Leones Don't know ☐

Pharmacy, drug peddler, pepper Dr. ☐ no. of visits ___
How much was spent on;
Consultation _________________Leones Don't know ☐
Medications _________________Leones Don't know ☐
Medical supplies ______________Leones Don't know ☐
Investigations ________________Leones Don't know ☐
Transportation _______________Leones Don't know ☐
Other ______________________Leones Don't know ☐
OR Estimated total ____________Leones Don't know ☐

Traditional Healer / Herbalist ☐ no. of visits ___

Other government hospital ☐ no. of visits ___
How much was spent on;
Consultation _________________Leones Don't know ☐
Medications _________________Leones Don't know ☐
Medical supplies ______________Leones Don't know ☐
Investigations ________________Leones Don't know ☐
Transportation _______________Leones Don't know ☐
Other ______________________Leones Don't know ☐
OR Estimated total ____________Leones Don't know ☐

Private hospital ☐ no. of visits ___
How much was spent on;
Consultation _________________Leones Don't know ☐
Medications _________________Leones Don't know ☐
Medical supplies ______________Leones Don't know ☐
Investigations ________________Leones Don't know ☐
Transportation _______________Leones Don't know ☐
Other ______________________Leones Don't know ☐
OR Estimated total ____________Leones Don't know ☐

Community health clinic (Cheifdom level) ☐
no. of visits ___
How much was spent on;
Consultation _________________Leones Don't know ☐
Medications _________________Leones Don't know ☐
Medical supplies ______________Leones Don't know ☐
Investigations ________________Leones Don't know ☐
Transportation _______________Leones Don't know ☐
Other ______________________Leones Don't know ☐
OR Estimated total ____________Leones Don't know ☐

For peer review only

	How much was spent on;<br>Consultation ____________Leones Don't know ☐<br>Medications ____________Leones Don't know ☐<br>Medical supplies ____________Leones Don't know ☐<br>Investigations ____________Leones Don't know ☐<br>Transportation ____________Leones Don't know ☐<br>Other ____________Leones Don't know ☐<br>**OR** Estimated total __________Leones Don't know ☐<br><br>**Traditional Birth Attendant (TBA) /Community Health Worker came home** ☐ no. of visits ___<br>How much was spent on;<br>Consultation ____________Leones Don't know ☐<br>Medications ____________Leones Don't know ☐<br>Medical supplies ____________Leones Don't know ☐<br>Investigations ____________Leones Don't know ☐<br>Transportation ____________Leones Don't know ☐<br>Other ____________Leones Don't know ☐<br>**OR** Estimated total __________Leones Don't know ☐<br><br>**Home delivery** ☐ no. of visits _____<br>How much was spent on;<br>Consultation ____________Leones Don't know ☐<br>Medications ____________Leones Don't know ☐<br>Medical supplies ____________Leones Don't know ☐<br>Investigations ____________Leones Don't know ☐<br>Transportation ____________Leones Don't know ☐<br>Other ____________Leones Don't know ☐<br>**OR** Estimated total __________Leones Don't know ☐	**Community health post** ☐ no. of visits ___<br>How much was spent on;<br>Consultation ____________Leones Don't know ☐<br>Medications ____________Leones Don't know ☐<br>Medical supplies ____________Leones Don't know ☐<br>Investigations ____________Leones Don't know ☐<br>Transportation ____________Leones Don't know ☐<br>Other ____________Leones Don't know ☐<br>**OR** Estimated total __________Leones Don't know ☐<br><br>**Maternity Child Health Post (MCHP)** ☐ no. of visits ___<br>How much was spent on;<br>Consultation ____________Leones Don't know ☐<br>Medications ____________Leones Don't know ☐<br>Medical supplies ____________Leones Don't know ☐<br>Investigations ____________Leones Don't know ☐<br>Transportation ____________Leones Don't know ☐<br>Other ____________Leones Don't know ☐<br>**OR** Estimated total __________Leones Don't know ☐<br><br>**Other** ☐ specify ______________no. of visits ___<br>How much was spent on;<br>Consultation ____________Leones Don't know ☐<br>Medications ____________Leones Don't know ☐<br>Medical supplies ____________Leones Don't know ☐<br>Investigations ____________Leones Don't know ☐<br>Transportation ____________Leones Don't know ☐<br>Other ____________Leones Don't know ☐<br>**OR** Estimated total __________Leones Don't know ☐

Section 4: Out-of-pocket payments related to hospital admission
"I would now like to ask you about all the costs to your household from travel to the hospital to admission, your whole hospital stay and treatment through to discharge"

Please reiterate that as with all questions any questions related to informal payments / payments directly to staff are completely anonymous and no names are recorded or required. The purpose is to understand how much patients have to pay for all types of costs associated with their hospital admission so the following questions should be answered without undue concern.

If the household did not incur any of the expenses listed please state 0 Leones (this applies throughout the questionnaire)

Code	Question	Response
E1	Once the referral or decision was made to come to Connaught/PCMH, **how did you and all the attendants come / travel**?	Ambulance ☐<br>Public transport - Taxi / KK /Okada ☐<br>Private transport ☐<br>Other ☐ specify ________________
E2	How much did this **transport cost?**	____________Leones Don't know ☐
E3	Did you have to pay any money for **registration**? If yes, ask how much the household paid for registration fees?<br><br>Tick all that are applicable	____________Leones Don't know ☐<br>Who or where was that paid to;<br>- Bank in Connaught ☐<br>____________Leones<br>- Directly to hospital staff ☐<br>____________Leones<br>- Other ☐ specify ____________<br>____________Leones
E4	Did you have to pay any money for **admission** (fees / booklet)? If yes, ask how much the household paid for admission?<br><br>Tick all that are applicable	____________Leones Don't know ☐<br>Who or where was that paid to;<br>- Bank in Connaught ☐<br>____________Leones<br>- Directly to hospital staff ☐<br>____________Leones

For peer review only - http://bmjopen.bmj.com/site/about/guidelines.xhtml

		- Other ☐ specify ________________<br>________________Leones
E5	Did you have to pay any money at or for **triage (e.g for blood sugar)**? If yes, ask how much the household paid?<br><br>Tick <u>all</u> that are applicable	________________Leones Don't know ☐<br>Who or where was that paid to;<br>- Bank in Connaught ☐<br>________________Leones<br>- Directly to hospital staff ☐<br>________________Leones<br>- Other ☐ specify ________________<br>- ________________Leones
E6	Did you have to pay any money for any **helpers** (e.g porters, wheelchair, hospital staff to help you get around etc). If yes, ask how much the household paid?	________________Leones Don't know ☐
E7	Did you have to pay any **doctors' fees / consultation fees**? If yes, ask how much the household paid in doctors' fees / consultation fees?<br><br>Tick <u>all</u> that are applicable	________________Leones Don't know ☐<br>Who or where was that paid to;<br>- Bank in Connaught ☐<br>________________Leones<br>- Directly to healthcare workers ☐<br>________________Leones<br>- Other ☐ specify ________________<br>________________Leones
E8	Did you have any **laboratory (lab) tests**? If yes, ask how much money the household spent on these tests?<br><br>Tick <u>all</u> that are applicable	________________Leones Don't know ☐<br>Who or where was that paid to;<br>- Bank in Connaught ☐<br>________________Leones<br>- External lab ☐<br>________________Leones<br>- Internal lab ☐<br>________________Leones<br>- Directly to healthcare workers ☐<br>________________Leones<br>- Other ☐ specify ________________<br>________________Leones
E9	Did you have any **x-rays, scans or imaging**? If yes, ask how much money your household spent on scans, imaging and x-rays?<br><br>Tick <u>all</u> that are applicable	________________Leones Don't know ☐<br>Who or where was that paid to;<br>- Bank in Connaught ☐<br>________________Leones<br>- External facility ☐<br>________________Leones<br>- Internal facility ☐<br>________________Leones<br>- Directly to healthcare workers ☐<br>________________Leones<br>- Other ☐ specify ________________<br>________________Leones
E10	Did the patient have an **operation or procedure** (e.g skin traction/POP/suturing) in hospital?	Yes ☐ No ☐ (go to E15)
E11	How much did you pay for this **operation or procedure**?<br><br>Tick <u>all</u> that are applicable	________________Leones. Don't know ☐<br>Who or where was that paid to;<br>- Bank in Connaught ☐<br>________________Leones<br>- Directly to healthcare workers ☐<br>________________Leones<br>- Other ☐ specify ________________<br>________________Leones
E12	Did you have to pay for **medications or medical supplies** (e.g. cannulas, IV lines, gauze, bandages, dressings, gloves, catheters and other consumables) **for theatre / your operation/procedure**? If yes, how much money did your household spend on these medicines and medical supplies?	________________Leones. Don't know ☐<br>Who or where was that paid to;<br>- Bank in Connaught ☐<br>________________Leones<br>- External pharmacy ☐<br>________________Leones

For peer review only - http://bmjopen.bmj.com/site/about/guidelines.xhtml

	Tick all that are applicable	- Internal pharmacy / cost recovery ☐ ______________Leones<br>- Directly to healthcare workers ☐ ______________Leones<br>- Other ☐ specify ________________ ______________Leones
E13	Did you have any **extra charges once in the operating room /area**? Please specify what this was for.	Yes ☐ (specify) No ☐ (go to E15)<br>**Specify what this was for?**<br>____________________________
E14	How much money did your household spend on these **extra-charges in the operating room?**<br><br>Tick all that are applicable	________________Leones. Don't know ☐<br>Who or where was that paid to;<br>- Bank in Connaught ☐ ______________Leones<br>- External pharmacy ☐ ______________Leones<br>- Internal pharmacy / cost recovery ☐ ______________Leones<br>- Directly to healthcare workers ☐ ______________Leones<br>- Other ☐ specify ________________ ______________Leones
E15	Did your household have to arrange and get **blood for transfusion**?	Yes ☐ No ☐ (go to E18)
E17	How much money did your household spend on **blood transfusion related costs** (including blood bank fees, supplies, paying a donor etc.)<br><br>Tick all that are applicable	________________Leones Don't know ☐<br>Who or where was that paid to;<br>- Bank in Connaught ☐ ______________Leones<br>- External facility ☐ ______________Leones<br>- Internal facility ☐ ______________Leones<br>- Directly to healthcare workers ☐ ______________Leones<br>- Other ☐ specify ________________ ______________Leones
E18	Did you have to pay for any **medications in A&E/OPD or on the ward** (excluding prescriptions specifically for theatre)? If yes, ask how much the household paid for this?<br><br>Tick all that are applicable	________________Leones Don't know ☐<br>Who or where was that paid to;<br>- Bank in Connaught ☐ ______________Leones<br>- External pharmacy ☐ ______________Leones<br>- Internal pharmacy / cost recovery ☐ ______________Leones<br>- Directly to healthcare workers ☐ ______________Leones<br>- Other ☐ specify ________________ ______________Leones
E19	Did you have to pay for any **medical supplies in A&E/OPD and the Ward** (not including medications, e.g. cannulas, IV lines, gauze, bandages, dressings, gloves, catheters and other consumables) (excluding the prescription for theatre))? If yes, ask how much was paid?<br><br>Tick all that are applicable	________________Leones. Don't know ☐<br>Who or where was that paid to;<br>- Bank in Connaught ☐ ______________Leones<br>- External pharmacy / facility ☐ ______________Leones<br>- Internal pharmacy / facility / cost recovery ☐ ______________Leones<br>- Directly to healthcare workers ☐ ______________Leones<br>- Other ☐ specify ________________ ______________Leones
E20	Did you have to pay any **bed fees** (excluding admission fees / book? If yes, ask how much money the household paid in bed fees?	______________Leones Don't know ☐<br>Who or where was that paid to;<br>- Bank in Connaught ☐

For peer review only

	Tick all that are applicable	______________Leones<br>- Directly to healthcare workers ☐<br>______________Leones<br>- Other ☐ specify ________________<br>______________Leones
E21	Did your household pay any money for **nursing care** (**requested** by nurses for care such as dressing changes etc, not including medications or medical supplies)? If yes, ask how much was paid?	______________Leones Don't know ☐<br>Who or where was that paid to;<br>- Bank in Connaught ☐<br>______________Leones<br>- Directly to healthcare workers ☐<br>______________Leones<br>- Other ☐ specify ________________<br>______________Leones
E22	Did your household give any **tips (ahjo)** i.e. voluntary and not requested money / gifts to healthcare workers / staff? If yes ask how much this amounted to?	________________Leones Don't know ☐
E23	How many **family members / attendants / persons stayed in hospital** during your hospital stay (excluding visitors)?	____________________people
E24	**Where did they stay** and **how much** did your household pay towards this?	In hospital ☐<br>Outside accommodation ☐<br>Other ☐ specify ______________________<br>Total cost ____________Leones Don't know ☐
E25	During your hospital stay **where did you get food from**?<br><br>Tick all that are applicable	Provided by the hospital ☐<br>Bought by the patient / household ☐<br>Brought for the patient by someone else ☐<br>Other ☐ specify ____________________________
E26	How much did you / your household **spend on food** during your hospital stay? If they can't give a break down then ask for a total.	On patient ______________Leones Don't know ☐<br>For attendants ___________Leones Don't know ☐<br>Total _______________Leones Don't know ☐
E27	Did your household spend any money on transport for all the **"running" (transport to get food, hospital supplies, investigations and medications externally, excluding transport for visitors)**? If yes ask how much and state what this was for?<br>Tick all that are applicable	________________Leones Don't know ☐<br>**What was this for?**<br>Food for the patients / attendants ☐<br>Going for tests / investigations outside ☐<br>Getting medicines / medical supplies ☐<br>Other ☐ specify ________________
E28	Did your household have to pay any money at the time of **discharge**? If yes, ask how much was paid?	________________Leones. Don't know ☐<br>Who or where was that paid to;<br>- Bank in Connaught ☐<br>______________Leones<br>- Directly to healthcare workers ☐<br>______________Leones<br>- Other ☐ specify ______________<br>______________Leones
E29	Did your household have **any other illness related / hospital costs (e.g at SOP / other departments visited)**? If yes, specify what these costs where for.	Yes ☐ (specify and go to E30) No ☐ (go to F1)<br>**Specify**____________________________________
E30	How much did these **other costs** amount to?	________________Leones. Don't know ☐<br>Who or where was that paid to;<br>- Bank in Connaught ☐<br>______________Leones<br>- Directly to healthcare workers ☐<br>______________Leones<br>- Other ☐ specify ______________<br>______________Leones

Section 5: Indirect non-medical expenditure and how costs are met
"I will now ask you some questions about the broader implications of the costs and how you have met these costs?"

Code	Question	Response
F1	Do you have **health insurance/medical care** to cover any medical costs?	Yes ☐ No ☐ (go to F5)<br>Don't know ☐
F2	What **type of health insurance** do you have?	☐ Private insurance<br>☐ Work-related insurance

For peer review only - http://bmjopen.bmj.com/site/about/guidelines.xhtml

		☐ Other (specify)________________________
F3	What is your **monthly contribution towards your insurance** (if applicable)?	________________Leones Don't know ☐
F4	Does **this insurance cover all (total) medical costs?**	Yes ☐ No ☐ If no, how much was paid in addition ____________Leones Don't know ☐
F5	**How did your household pay** for all the above costs? Tick all that are applicable	
	**Use savings**? Yes ☐ No ☐ Don't know ☐	How much? _______________Leones Don't know ☐
	**Borrow money**? Yes ☐ No ☐ Don't know ☐	How much?_______________Leones<br>Does this need to be paid back? Yes ☐ No ☐ Don't know ☐
	**Arrange family contributions?** Yes ☐ No ☐ Don't know ☐	How much?_______________Leones<br>Does this need to be paid back? Yes ☐ No ☐ Don't know ☐
	**Charity money from family/friends outside the country/church/social clubs?** Yes ☐ No ☐ Don't know ☐	How much?_______________Leones<br>Does this need to be paid back? Yes ☐ No ☐ Don't know ☐
	**Pledge / pawn any possessions** (including livestock, assets including electrical goods, generator, watches, jewellery etc.)?<br>Yes ☐ No ☐ Don't know ☐	How much money did this raise?_________________Leones<br>Don't know ☐
	**Sell any possessions or land?** Yes ☐ No ☐ Don't know ☐	How much money did this raise?_________________Leones<br>Don't know ☐
	**Any other way of meeting the hospital costs?**<br>Yes ☐ No ☐ Don't know ☐	Specify how __________________________________<br>How much money did this raise?_________________Leones<br>Don't know ☐
F6	Did your household have to **stop sending any children to school, or pay reduced school fees** in order to pay for this hospitalisation?	Yes ☐ No ☐ Don't know ☐
F7	Did your household **loose any wages** due to this hospital stay? If yes, ask how much was lost?	Yes ☐ No ☐ Don't know ☐<br>How much was lost ________________Leones<br>Don't know ☐
F8	Did you or anyone in your household **lose their job or change their role at work or home?**	Yes ☐ No ☐ Don't know ☐<br>If yes please specify<br>Lost job ☐<br>Took up employment / 2nd job ☐<br>Change of duties in household to meet costs ☐<br>Other ☐ specify ________________________________

Any other comments?

____
____
____
____

Appendix table 2: Household expenditure showing imputed and non-imputed data sets.
Comparison of non-imputed and imputed data on household expenditure using Multiple Imputation Chained Equations to compute missing data-points using predictive mean matching.

Household expense	Non-imputed data mean (SD) (Le and $US)	Imputed data pooled mean (Le and $ US)
**Individual Consumption Expenditure by Households Including all variables collected**	**Le 39,665,597 (53,679,740) $ 4,425 (5,989)**	**Le 47,944,384 $5,349**
**Individual Consumption Expenditure by Households Excluding variables with > 20% missing data i.e. clothing, mobile phone credit and transport**	**Le 28,134,505 (31,539,987) $ 3,139 (3,519)**	**Le 31,988,507 $ 3,569**
Food and non-alcoholic beverages	Le 18,616,404	Le 20,867,118

For peer review only - http://bmjopen.bmj.com/site/about/guidelines.xhtml

	(22,364,391)<br>$ 2,077 (2,495)	$ 2,328
Alcoholic Beverages, Tobacco and Narcotics	Le 252,991<br>(1,047,612)<br>$ 28 (117)	Le 314,095<br>$ 35
Rental	Le 884,123<br>(4,670,009)<br>$ 99 (521)	Le 876,940<br>$ 98
Household maintenance	Le 108,092<br>(298,466)<br>$ 12 (33)	Le 130,857<br><br>$ 15
Electricity, gas and other fuels	Le 720,748<br>(1,068,694)<br>$ 80 (119)	Le 747,701<br>$ 83
Furnishings, household equipment and routine household maintenance	Le 632,577<br>(1,065,761)<br>$ 71 (119)	Le 699,188<br>$ 78
Healthcare (traditional and western medicine)	Le 561,770<br>(1,490,207)<br>$ 63 (166)	Le 740,205<br>$ 83
Recreation and cultural services	Le 821,684<br>(1,894,677)<br>$ 92 (211)	Le 1,121,965<br>$ 125
Education	Le 1,338,183<br>(2,295,578)<br>$ 149 (256)	Le 1,614,982<br>$ 180
Personal care / toiletries	Le 609,012<br>(629,656)<br>$ 68 (70)	Le 627,269<br>$ 70
Health insurance	Le 8,333<br>(138,661)<br>$ 1 (15)	Le 13,370<br>$ 1
Remittance	Le 974,408<br>(1,655,020)<br>$ 109 (185)	Le 1,015,363<br>$ 113
Donations	Le 185,233<br>(475,436)<br>$ 21 (53)	Le 203,246<br>$ 23
Livestock	Le 41,400<br>(213,804)<br>$ 5 (24)	Le 45,254<br>$5
Taxes	Le 59,500<br>(261,497)<br>$ 7 (29)	Le 73,723<br>$ 8

Appendix table 3: Household expenditure data. Variables on household expenditure shown here, for broad comparison, with the Economic and Financial survey Sierra Leone 2014 data[21]. Categories were harmonised where possible, however given differences in questions asked between surveys, an exact match of categories was not possible to achieve. Costs from the 2014 Economic and Financial Survey were not adjusted for inflation which needs to be considered when reviewing this data.

**Household consumption expenditure (in Leones (Le) and USD ($))**		
**Household Expense**	**Sierra Leone Economic and Financial Survey data 2014**	**Study data**
**Individual Consumption Expenditure by Households (Total Expenditure)**	**Le 15,414,816**<br>**$ 1,739**	**Le 31,988,507**<br>**$ 3,569**

For peer review only - http://bmjopen.bmj.com/site/about/guidelines.xhtml

**Food and non-alcoholic beverages**	**Le 6,838,365**<br>**$ 771**	**Le 20,867,118**<br>**$ 2,328**
Food	Le 6,644,019<br>$ 74	Le 17,925,090<br>$ 2,000
Non-alcoholic beverages	Le 194,346<br>$ 22	Le 2,942,028<br>$ 328
**Alcoholic Beverages, Tobacco and Narcotics**	**Le 450,612**<br>**$ 51**	**Le 314,095**<br>**$ 35**
**Housing, water, electricity, gas and other fuels**	**Le 1,058,449**<br>**$119**	**Le 875,672**<br>**$ 98**
**Rental**	**Le 253,948**<br>**$ 29**	**Le 876,940**<br>**$ 98**
**Maintenance and repair of the dwelling**	**Le 32,650**<br>**$ 4**	**Le 876,940**<br>**$ 98**
**Electricity, gas and other fuels**	**Le 595,832**<br>**$ 67**	**Le 747,701**<br>**$ 83**
**Furnishings, household equipment and routine household maintenance**	**Le 413,364**<br>**$ 47**	**Le 699,188**<br>**$ 78**
Furnishings	Le 88,058<br>$ 10	Le 196,217<br>$ 22
Household appliances	Le 41,821<br>$ 5	Le 314,772<br>$ 35
Tools and equipment for house and garden	Le 45,403<br>$ 5	Le 57,344<br>$ 6
Goods and services for routine household maintenance	Le 105,438<br>$ 12	Le 130,857<br>$ 15
Purchase of vehicles	Le 108,710<br>$ 12	Le 160,759<br>$ 18
Educations	Le 794,478<br>$ 90	Le 1,614,982<br>$ 180
Insurance (HI)	Le 38,890<br>$ 4	Le 13,370<br>$ 1

For peer review only

Appendix table 4: Imputed and non-imputed data for out-of-pocket costs for comparison.

Costs	Imputed<br>Mean cost<br>($US (% of subtotal))	Non-imputed<br>Mean cost ($US) (SD)
**Prehospital costs**		
**Direct pre-hospital medical OOP costs (total)**	**21 (88% of 24)**	**14 (65)**
- Consultation	2 (10% of 21)	1 (6)
- Medications	12 (57% of 21)	9 (46)
- Medical supplies	2 (10% of 21)	2 (8)
- Investigations	4 (19% of 21)	4 (25)
- Other miscellaneous	2 (10% of 21)	1 (10)
**Direct (pre-hospital) non-medical OOP costs (total)**	**3 (13% of 24)**	**3 (9)**
- Transport	3 (100% of 3)	3 (9)
Total pre-hospital costs	***24 (10% of 243)***	***25 (75)***
**In hospital costs**		
**Direct medical OOP costs (total)**	**138 (63% of 219)**	**109 (121)**
- Administrative	20 (14% of 138)	16 (24)
- Medications	26 (19% of 138)	25 (61)
- Medical supplies	14 (10% of 138)	11 (33)
- Investigations	15 (11% of 138)	14 (23)
- Blood transfusion	9 (7% of 138)	9 (22)
- Total operation costs	49 (36% of 138)	51 (75)
- Unofficial costs	6 (4% of 138)	5 (9)
- Other / miscellaneous	1 (1% of 138)	1 (8)
**Direct non-medical costs (total)**	**34 (16% of 219)**	**34 (34)**
- Transport to hospital	7 (21% of 34)	7 (17)
- Food	20 (59% of 34)	21 (20)
- Accommodation	0 (0% of 34)	0 (0)
- Other*	7 (21% of 34)	6 (10)
**Indirect costs**		
- Lost wages	46 (100% of 46)	35 (116)
**TOTAL OOP COSTS**	**243**	**176 (165)**

*other relates to travel and other associated costs incurred as a result for needed investigations from and or medication / supplies from an external facility. SPSS calculates only the mean using imputed variables, hence no standard deviation is displayed.

For peer review only

Appendix table 5: Route of payment for OOP costs; percentage of the total OOP costs by cost categories paid to bank / cashier, directly to staff or externally for different services accessed once tertiary level care was reached

Costs	% paid to Hospital bank / cashier	% paid directly to or via staff	% paid externally	% unknown	Total
**TOTAL in-hospital costs**	**32.64%**	**48.16%**	**16.50%**	**2.70%**	**100%**
**Administration**	**52.03%**	**42.53%**	-	5.44%	100%
- Registration fees	90.98%	8.71%	-	0.30%	100%
- Admission fees	66.78%	32.39%	-	0.83%	100%
- Triage fees	8.05%	91.95%	-	0.00%	100%
- Bed fees	19.47%	46.13%	-	34.40%	100%
- Discharge fees	41.19%	57.28%	-	1.53%	100%
**Investigations**	**19.39%**	**44.29%**	**33.73%**	**2.59%**	**100%**
- Laboratory	25.68%	40.29%	30.13%	3.90%	100%
- Imaging	14.43%	47.44%	36.57%	1.56%	100%
**Total operation costs**	**55.40%**	**32.89%**	**9.35%**	**2.35%**	**100%**
- Operation	80.10%	18.91%	-	1.00%	100%
- Medical supplies for the operation	13.42%	56.59%	25.31%	4.68%	100%
- Other / miscellaneous	69.77%	30.23%	-	0.00%	100%
**Blood transfusion**	**16.24%**	**67.68%**	**13.85%**	**2.23%**	**100%**
**Total medications and medical supplies for ward care**	**4.82%**	**61.00%**	**31.73%**	**2.45%**	**100%**
- Medications	2.19%	62.86%	31.91%	3.03%	100%
- Medical supplies	10.81%	56.74%	31.31%	1.14%	100%
**Informal payment**	**2.44%**	**97.56%**	**-**	**0.00%**	**100%**
- Doctors' fees	8.72%	91.28%	-	0.00%	100%
- Nursing care	0%	100%	-	0.00%	100%
- Porters	0%	100%	-	0.00%	100%
- Tips	0%	100%	-	0.00%	100%
**Other / miscellaneous costs**	**0%**	**98.37%**	**0**	**1.63%**	**100%**

For peer review only - http://bmjopen.bmj.com/site/about/guidelines.xhtml

Appendix table 6: Linear regression analysis showing odds of increasing in-hospital costs of care for each variable using a generalized linear model with imputed variables using a Tweedie 1.9 function.

Variable		Odds Ratio	95% CI	p-value
Sex	Female	ref		
	Male	1.05	(0.86-1.29)	0.63
Age	Age	1.02	(1.01-1.02)	0.00
Length of stay	Length of stay	1.02	(1.02 -1.03)	0.00
Type of admission	Elective admission	ref		
	Emergency admission	0.96	(0.75-1.24)	0.77
Category of operation	Non-operative	ref		
	Burns	1.33	(0.25-7.00)	0.74
	ENT	0.64	(0.36-1.166)	0.14
	General surgery	1.67	(1.29-2.17)	0.00
	General paediatric surgery	0.84	(0.57-1.24)	0.38
	Trauma and orthopaedic	1.30	(0.98- 1.74)	0.07
	Urology	2.08	(1.22-3.53)	0.01
Area of residence	Rural	ref		
	Urban	0.98	(0.76-1.25)	0.85

For peer review only

STROBE Statement—Checklist of items that should be included in reports of ***cross-sectional studies***

	Item No	Recommendation	Page number
**Title and abstract**	1	(*a*) Indicate the study's design with a commonly used term in the title or the abstract	1-3
		(*b*) Provide in the abstract an informative and balanced summary of what was done and what was found	1-3
**Introduction**			**4**
Background/rationale	2	Explain the scientific background and rationale for the investigation being reported	4
Objectives	3	State specific objectives, including any prespecified hypotheses	4
**Methods**			**5-7**
Study design	4	Present key elements of study design early in the paper	5
Setting	5	Describe the setting, locations, and relevant dates, including periods of recruitment, exposure, follow-up, and data collection	5
Participants	6	(*a*) Give the eligibility criteria, and the sources and methods of selection of participants	5
Variables	7	Clearly define all outcomes, exposures, predictors, potential confounders, and effect modifiers. Give diagnostic criteria, if applicable	5-7
Data sources/ measurement	8*	For each variable of interest, give sources of data and details of methods of assessment (measurement). Describe comparability of assessment methods if there is more than one group	*5-7*
Bias	9	Describe any efforts to address potential sources of bias	10-11
Study size	10	Explain how the study size was arrived at	7
Quantitative variables	11	Explain how quantitative variables were handled in the analyses. If applicable, describe which groupings were chosen and why	7
Statistical methods	12	(*a*) Describe all statistical methods, including those used to control for confounding	7
		(*b*) Describe any methods used to examine subgroups and interactions	7
		(*c*) Explain how missing data were addressed	7
		(*d*) If applicable, describe analytical methods taking account of sampling strategy	n/a
		(*e*) Describe any sensitivity analyses	7
**Results**			**8-9**
Participants	13*	(a) Report numbers of individuals at each stage of study—eg numbers potentially eligible, examined for eligibility, confirmed eligible, included in the study, completing follow-up, and analysed	8-9 Figure 1
		(b) Give reasons for non-participation at each stage	Figure 1
		(c) Consider use of a flow diagram	Figure 1
Descriptive data	14*	(a) Give characteristics of study participants (eg demographic, clinical, social) and information on exposures and potential confounders	8 Table 1
		(b) Indicate number of participants with missing data for each variable of interest	Table 1
Outcome data	15*	Report numbers of outcome events or summary measures	8-9 Table 1 Table 2

For peer review only

			Table 3<br>Table 4
Main results	16	(*a*) Give unadjusted estimates and, if applicable, confounder-adjusted estimates and their precision (eg, 95% confidence interval). Make clear which confounders were adjusted for and why they were included	n/a
		(*b*) Report category boundaries when continuous variables were categorized	8-9<br>Tables
		(*c*) If relevant, consider translating estimates of relative risk into absolute risk for a meaningful time period	n/a
Other analyses	17	Report other analyses done—eg analyses of subgroups and interactions, and sensitivity analyses	Appendix 1, 2, 3
**Discussion**			**9 - 10**
Key results	18	Summarise key results with reference to study objectives	9-10
Limitations	19	Discuss limitations of the study, taking into account sources of potential bias or imprecision. Discuss both direction and magnitude of any potential bias	10-11
Interpretation	20	Give a cautious overall interpretation of results considering objectives, limitations, multiplicity of analyses, results from similar studies, and other relevant evidence	9-10
Generalisability	21	Discuss the generalisability (external validity) of the study results	10
**Other information**			
Funding	22	Give the source of funding and the role of the funders for the present study and, if applicable, for the original study on which the present article is based	1

*Give information separately for exposed and unexposed groups.

Note: An Explanation and Elaboration article discusses each checklist item and gives methodological background and published examples of transparent reporting. The STROBE checklist is best used in conjunction with this article (freely available on the Web sites of PLoS Medicine at http://www.plosmedicine.org/, Annals of Internal Medicine at http://www.annals.org/, and Epidemiology at http://www.epidem.com/). Information on the STROBE Initiative is available at www.strobe-statement.org.

For peer review only - http://bmjopen.bmj.com/site/about/guidelines.xhtml
